# Cross-talk of filter gate and lower helices drives polymodal regulation and disease in TREK channels

**DOI:** 10.1038/s41467-026-77935-w

**Published:** 2026-09-22

**Authors:** Berke Türkaydin, Valerio Rizzi, Chaimae Benkerdagh, Simon Ghysbrecht, Simone Aureli, Thomas Baukrowitz, Marcus Schewe, Francesco Luigi Gervasio, Han Sun

**Affiliations:** 1 Leibniz-Forschungsinstitut für Molekulare Pharmakologie (FMP), Berlin, Germany; 2 Insitute of Chemistry, Technical University of Berlin, Berlin, Germany; 3 School of Pharmaceutical Sciences, University of Geneva, Rue Michel Servet 1, Genève, Switzerland; 4 Institute of Pharmaceutical Sciences of Western Switzerland (ISPSO), University of Geneva, Genève, Switzerland; 5 Swiss Institute of Bioinformatics, University of Geneva, Genève, Switzerland; 6 Institute of Physiology, Kiel University, Kiel, Germany; 7 Department of Chemistry, University College London, London, United Kingdom

**Keywords:** Ion transport, Potassium channels, Protein analysis, Computational chemistry

## Abstract

The TREK two-pore domain potassium channels regulate membrane excitability and respond to diverse physiological stimuli. Single-site mutations of TREK are associated with neurodevelopmental disorders and can cause pathological channel hyperactivation. Here, using the OneOPES enhanced-sampling molecular dynamics framework, we characterize the energetics of conformational transition along a predefined pathway connecting the up and down states of TREK-2, together with initial conformational rearrangements of the selectivity filter under physiological stimuli and disease-mimicking conditions. The simulations reveal a shared energetic axis coupling the lower helices, proximal C-terminus, and selectivity filter to govern channel gating. Predicted conformational effects of a disease-mimicking mutation were validated electrophysiologically using a conformation-sensitive TREK-2 inhibitor. Together with our previous unbiased MD simulations, these results establish an energetic framework for TREK-2 regulation and dysfunction, complementing prior structural and mechanistic studies while highlighting the potential and methodological challenges of advanced enhanced-sampling approaches for resolving complex conformational landscapes of ion channels.

## Abstract (web summary)

Here the authors perform advanced simulations and experiments to reveal how coordinated structural changes regulate TREK-2 potassium channel activity and how disease-associated mutations disrupt this mechanism, providing atomistic insights into neurological disorders.

## Introduction

The TREK subfamily of two-pore domain potassium (K_2P_) channels is a key regulator of neuronal excitability, contributing to background leak currents that stabilize the resting membrane potential below the threshold for action potential firing^1–3^. These channels respond to a wide spectrum of stimuli, including mechanical stretch, temperature, lipids, pH, and phosphorylation, demonstrating their ability to sense and integrate multiple physiological signals to modulate their activity^2,4–9^. Mechanical stretch acts as a major activating stimulus, linking TREK channel activity to changes in membrane tension that occur during neuronal firing, osmotic stress, and mechanical deformation^6,10^. Under increased membrane tension, TREK channels showed increased channel activity, leading to membrane hyperpolarization and reduced neuronal excitability. In contrast, phosphorylation at the protein kinase C (PKC) site within the proximal C-terminus (pCt) domain provides a chemical pathway for inhibition, shifting the channel toward the less-conductive down-state conformation and suppressing background leak currents^11–17^.

Based on this regulatory profile, TREK channels are implicated in processes such as neuroprotection, anesthesia, pain perception, and depression, and are recognized as promising pharmacological targets^12,18–20^. Recent genetic and electrophysiological studies have further highlighted that disruption of this regulatory balance can lead to pathological channel behavior. Gain-of-function (GoF) mutations within the TRAAK (*KCNK4*) channel have been associated with severe neurodevelopmental disorders marked by neuronal hyperexcitability^21^. Two distinct de novo missense variants (A244P and A172E) located near the M4 and M3 helices were identified in patients with FHEIG syndrome, a condition characterized by facial dysmorphism, hypertrichosis, epilepsy, intellectual disability, and gingival overgrowth. [Note that in the numbering scheme of the TRAAK crystallographic construct (PDB ID: *4WFE*)^22^, these residues are referred to as A270 and A198, respectively (Supplementary Table 1)]. Functional analyses revealed that these mutations greatly enhance basal potassium currents and impair normal regulatory responses to mechanical or chemical stimuli, producing constitutively active channels^23^. These findings demonstrate that even single-residue mutations can disrupt gating control of TREK K_2P_ channels, leading to persistent activation and disease phenotypes.

**Fig. 1:**
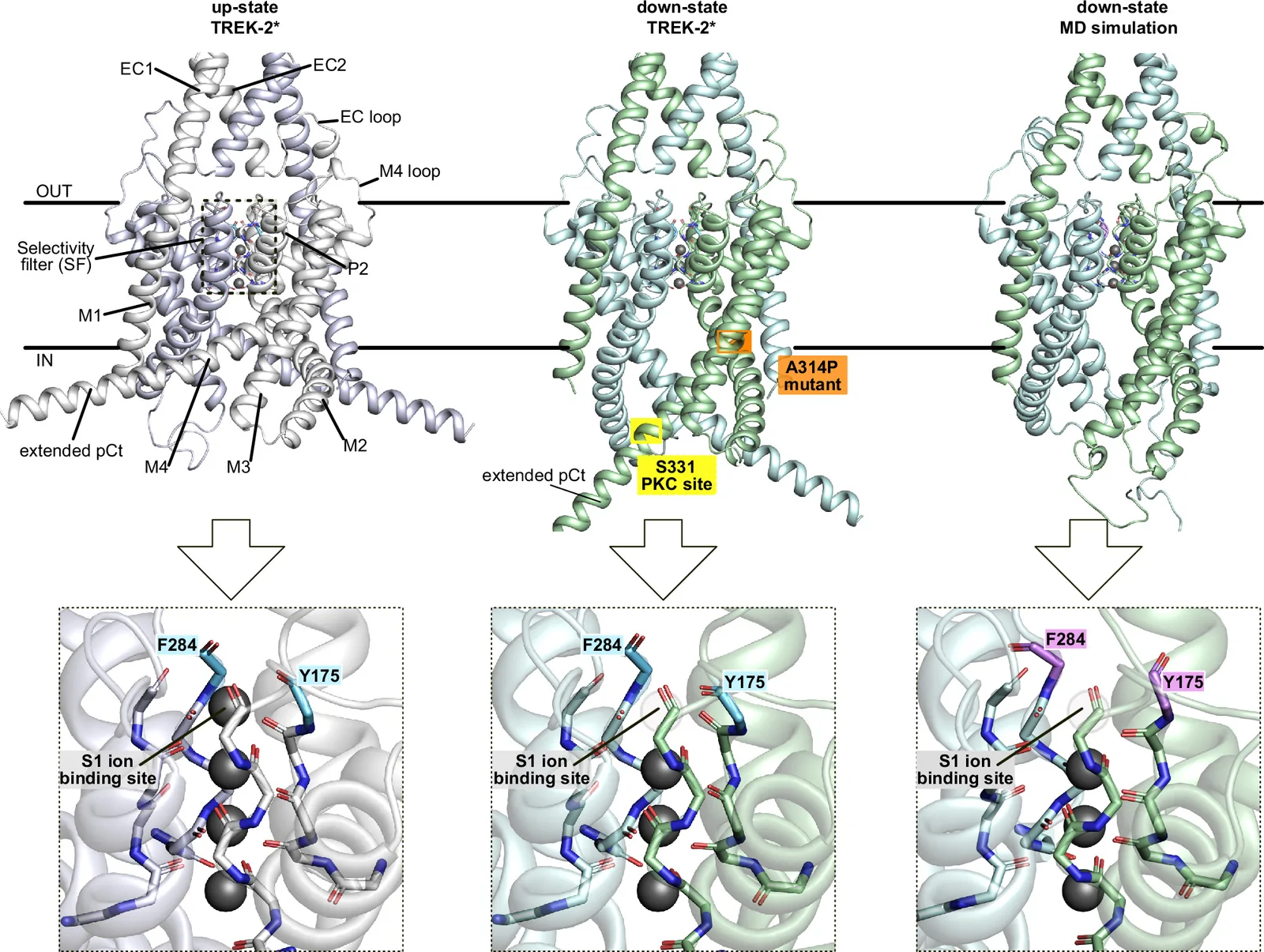
Conformational states and constructs of TREK-2 K_2P_ channel used in the present MD study. Crystal structures of TREK-2 in the up-state (PDB ID: *4BW5*)^24^ and down-state (PDB ID: *4XDJ*)^24^ with the 19-residue pCt modeled in an α-helical conformation (designated TREK-2*; see “Methods”: MD-based system preparation), along with one representative final snapshot from our previous unbiased MD simulations^17^. The PKC phosphorylation site S331 within the pCt region and the investigated A314P mutant site are highlighted by yellow and orange boxes, respectively. Key conformational rearrangements of the SF residues F284 and Y175 suggested by our previous unbiased MD simulations are highlighted in cyan and purple, respectively^17^.

Based on X-ray crystallographic studies, TREK and TRAAK channels adopt two major conformational states, referred to as the up- and down-states (Fig. 1)^24–27^. These states are primarily distinguished by the orientation of their transmembrane helix M4 relative to the membrane and by the positioning of the cytoplasmic C-terminus (Ct) domain. In particular, the pCt, which lies directly adjacent to the M4 helix, has been identified as a key regulatory domain that mediates the integration of multiple intracellular signals. Notably, the up- and down-state crystal structures of TREK/TRAAK channels exhibit nearly identical SF conformations, which argues against a canonical gate-switching mechanism at the filter. In the down-state, however, lateral fenestrations open and expose the region beneath the SF to the membrane, where additional electron density has been attributed to lipid acyl-chains. This observation led to the proposal of a lipid-block mechanism to explain the reduced conductance of the down-state^22,24^. In contrast, some electrophysiological and computational work, including our own, supported a model in which the SF itself serves as the primary gate in K_2P_ channels^7,28,29^. In addition, our recent study combining large-scale molecular dynamics (MD) simulations with extensive electrophysiological measurements suggested an allosteric mechanism by which the dynamics of M4 helix and pCt regulate the SF gate^17^. In down-state TREK-2 simulations, we observed pronounced backbone rearrangements near the S1 ion-binding site of the SF, which impaired ion conduction (Fig. 1). However, although these previous unbiased MD simulations captured a few spontaneous transitions between the up- and down-states, the slow timescale and large-amplitude motion prevented sufficient sampling for rigorous free-energy analysis. Consequently, unbiased simulations alone could not quantify the energetic coupling between motions in the M4/pCt and SF, thereby limiting our understanding of how different stimuli reshape the underlying free-energy landscape.

Over the past several decades, a number of enhanced-sampling approaches have been developed to accelerate sampling of rare conformational transitions in biomolecular systems^30–37^. However, their efficiency depends critically on how well a small number of collective variables (CVs) capture the relevant slow degrees of freedom. For large and flexible systems such as TREK channels, whose gating involves subtle long-range couplings between the cytosolic and transmembrane domains, defining an optimal low-dimensional set of CVs is particularly challenging. Standard metadynamics and similar enhanced sampling simulation methods often struggle to achieve proper convergence^31,38^. To overcome these challenges, we employ in the current study OneOPES, a recently developed extension of the On-the-fly Probability Enhanced Sampling (OPES) framework that combines adaptive biasing with replica-exchange sampling to explore high-dimensional free-energy landscapes^39^. In OneOPES, a primary replica applies an OPES Explore bias along a main CV, targeting accurate convergence for equilibrium properties. Multiple auxiliary replicas running in parallel apply weaker, more exploratory biases along additional CVs that might involve slow motion not sufficiently accounted for by the main CV. Some replicas further incorporate an OPES MultiThermal component, which effectively heats and cools the system, lowering free-energy barriers in a manner analogous to parallel-tempering. This approach accelerates transitions along degrees of freedom not explicitly included in the CVs, mitigating the effects of suboptimal CV selection. Exchanges between replicas enable efficient sampling of rare conformations, while rigorous reweighting ensures accurate reconstruction of unbiased free-energy surfaces (FES). These features have enabled OneOPES to achieve converged free energy landscapes for protein folding and large-scale conformational changes, even in complex biomolecular systems such as GPCRs and ion channels^40–42^. In this study, we apply OneOPES simulations to TREK-2 (*KCN10*) to elucidate how phosphorylation, membrane stretch, and pathogenic mutations influence the energetic landscape that is associated with the channel gating process. In particular, we combine a path-based CV with several auxiliary CVs designed to capture key conformational dynamics of both M4/pCt and SF regions, which were identified from our earlier unbiased MD simulations. Our predictions of how GoF mutations in TREK-2 homolog to the pathogenic FEIGH syndrome mutations in TRAAK alter the energetic landscape are further qualitatively supported by state-dependent norfluoxetine (NFx) inhibition experiments via inside-out patch-clamp measurements. Together, these findings provide an improved energetic framework for understanding how diverse stimuli and disease-associated mutations modulate TREK channel activity through shared energetic coupling mechanisms.

## Results

### The global conformational transition of TREK-2 is driven by the coordinated motion of M4-pCt

**Fig. 2:**
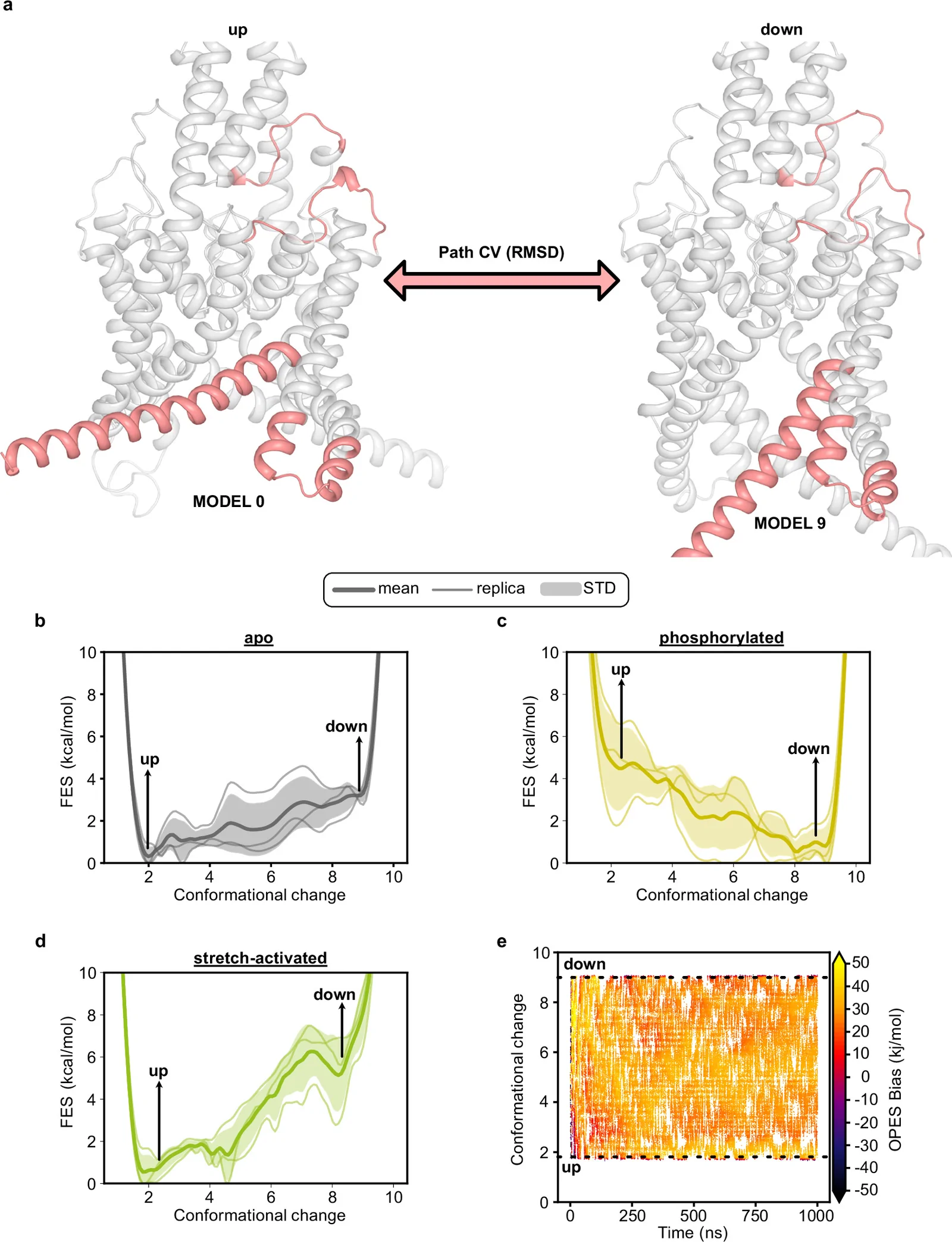
Path CV facilitates complete transition between two metastable states of TREK-2*. **a** Representation of the preparation of path CV used in OneOPES simulations. Up-state crystal structure (PDB ID: *4BW5*)^24^ was used as MODEL 0, and down-state crystal structure (PDB ID: *4XDJ*)^24^ was used as MODEL 9. Highly flexible regions that are affecting the RMSD changes were colored with salmon color, which were selected based on the crystal structures and observations based on our previous unbiased simulations^17^. **b** 1D free energy surface (FES) projected onto the path CV for apo, **c** phosphorylated and (**d**) membrane-stretch simulations. The solid line represents the mean of three independent simulations. The shaded region corresponds to the population standard deviation across the three kernel-reweighted free-energy profiles at each path-CV value (*n* = 3). This reflects run-to-run variability and does not include statistical uncertainty from binning. **e** Sampling of the path CV as a function of time in replica 0 of an apo TREK-2 OneOPES simulation. Data points are colored according to the accumulated OPES Explore bias potential applied along the path CV at each frame. The broad bias range observed during the early phase of the trajectory (~|50| kJ/mol) reflects the initial exploratory regime of OPES, during which the on-the-fly kernel density estimation (KDE) of the probability distribution is still rapidly evolving. The bias stabilizes as the simulation progresses (Supplementary Fig. 3), and the final FES is obtained by reweighting after discarding the first 100 ns of each replica.

We first selected three systems for investigations and comparisons: apo TREK-2, phosphorylated TREK-2, and a system subjected to mechanical stretch applied to the lipid bilayer. Following our previous study, we extended each TREK-2 subunit with the 19-residue pCt in an α-helical starting conformation, hereafter denoted TREK-2* (see “Methods”: MD-based system preparation for the structural justification)^17^. For each system, we performed three independent 1 μs OneOPES simulation runs, each consisting of eight replicas (Supplementary Table 2). To characterize the overall conformational behavior of these systems, we analyzed the one-dimensional free-energy surfaces (1D FES) obtained from the OneOPES simulations, projected onto the main path CV. This reaction coordinate is able to capture the large-scale structural transition between the crystallographically resolved up- and down-state conformations of the channel (for a detailed description of the CVs, see “Methods”: OneOPES simulations) (Fig. 2a). As with other path-based enhanced sampling approaches, the CV is designed to describe the essential dynamics connecting these predefined reference states and are therefore less effective at sampling conformations that lie far from the interpolated transition pathway, as also reflected in Supplementary Fig. 1.

The averaged 1D FES of the apo TREK-2* system displayed a relatively flat profile with the up-state stabilized by ~3 kcal/mol relative to the down state (Fig. 2b). This implies that the up-state is the dominant conformation in the absence of external stimuli, while the down-state remains thermally accessible. In the phosphorylated TREK-2* system, the FES shifted to a thermodynamically favored down-state (Fig. 2c), with an energy difference of about 4.7 kcal/mol. This is in line with the experimentally observed inhibitory effect of phosphorylation^17^. By contrast, mechanical stretch, simulated by applying a negative lateral pressure of −50 bar in the bilayer plane while maintaining +1 bar in the bilayer normal direction, at 300 K (see “Methods”: MD-based system preparation), markedly altered the energy profile (Fig. 2d). In this system, the 1D FES collapses into a single dominant minimum corresponding to the up-state, while the down-state is strongly disfavored. The free-energy difference between the two conformations exceeded 6 kcal/mol, suggesting a strong thermodynamic bias towards the up-state conformation under mechanical activation. These results are fully consistent with previous experimental and computational studies that identified mechanical stretch as a key regulator of TREK channel activation^10,43^. Convergence analysis of the OneOPES bias potential together with the time evolution of the auxiliary CVs, further supports the robustness of sampling along the predefined pathway across all replicas (Fig. 2e and Supplementary Fig. 2, 3). Figure 2e shows that replica 0 repeatedly traverses the full range of the path CV, demonstrating efficient sampling of the up- and down-state basins, as well as the intermediate conformational region. The color scale represents the accumulated OPES Explore bias applied along the path CV at each frame. Large positive and negative bias excursions are observed during the early phase of the trajectory and reflect the rapidly evolving probability-density estimate characteristic of the initial exploratory phase of OneOPES. As the simulation progresses, the bias stabilizes (Supplementary Fig. 3). The final FES is obtained by reweighting after discarding the first 100 ns of each replica, thereby preventing early-stage bias fluctuations from contributing to the converged free-energy profiles.

**Fig. 3:**
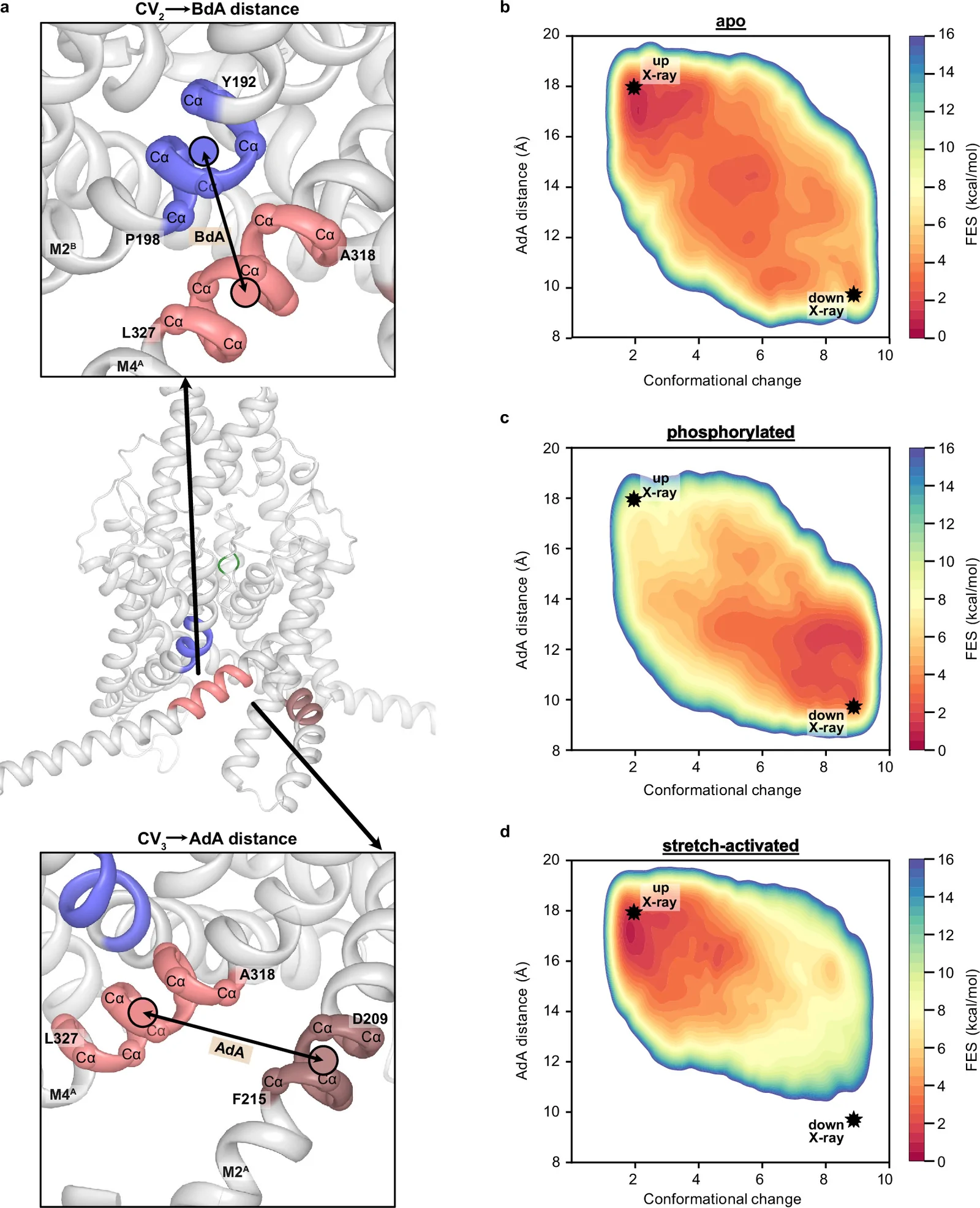
Structural rearrangements of the pCt provides energetic link to overall conformational dynamics. **a** Schematic representation of the two auxiliary CVs used to characterize the pCt movement. AdA denotes the center-of-mass distance between the M2 and M4 Cα atoms within the same subunit (subunit A M2 to subunit A M4, intra-chain), and BdA denotes the corresponding distance between opposite subunits (subunit B M4 to subunit A M2, inter-chain). **b** Two-dimensional free energy surfaces (2D FES) projected along the path CV and the auxiliary CV describing M4-M2 distance for apo, **c** phosphorylated and (**d**) membrane-stretch simulations. The average of three independent replica for each system was calculated for 2D FES. Black stars indicate the positions of the crystallographic TREK-2 up- and down-state structures mapped onto these coordinates, based on their measured path CV values and AdA distances.

To examine in greater details how the global up-down transitions are coupled to the structural rearrangements of the M4 helices, we next analyzed the two-dimensional free-energy surfaces (2D FES) spanned by the path CV and the two auxiliary CVs describing relative M4–M2 displacements (Fig. 3a). In our previous unbiased MD simulations, the pCt-M4 region was shown to undergo a pronounced vertical and lateral movement that redefines its interactions with the adjacent M2 helices^17^. Accordingly, the auxiliary CVs were specifically designed to quantify the relative displacement of the M4 helices with respect to the M2 helices. One auxiliary CV (AdA, denoting the M2–M4 distance from subunit A to subunit A, i.e., intra-chain) tracks the distance between the M4 and M2 helix within the same subunit, which decreases as the channel moves towards the down-state. The second CV (BdA, denoting the M2 of subunit B to the M4 of subunit A, i.e., inter-chain) monitors the distance between the M4 and M2 helix of opposite subunits, which instead decreases towards the up-state.

Across all systems, the 2D FES (Fig. 3) shows a strong correspondence between the M4–M2 displacement coordinates and the path CV: low path CV values (up-state) are associated with high AdA and low BdA, whereas high path CV values (down-state) display the opposite configuration (Fig. 3b–d and Supplementary Fig. 4). This co-variation can be considered as a geometric consequence of the path CV construction, since the up-to-down transition is dominated by M4 motion. Accordingly, the AdA and BdA auxiliary CVs decompose the global progress coordinate *s* into the specific intra- and inter-subunit M4–M2 displacements that drive the transition. Their role within the OneOPES setup is to provide additional driving forces along these slow, subunit-coupled motions. Consistent with the 1D FES, in the apo system, the 2D FES is relatively shallow and broadly dispersed across both CV axes, reflecting a more permissive and dynamically heterogenous motion of the M4 helix under equilibrium conditions. By contrast, phosphorylated and membrane-stretched systems exhibit markedly more localized free energy basins, with populations concentrated around a single, well-defined minimum. These compact free-energy landscapes indicate a substantial restriction of M4-M2 rearrangements: phosphorylation constrains the helix toward the down-state geometry, characterized by stronger intra-subunit M4-M2 interactions, whereas membrane stretch confines the system to the up-state ensemble, in which intra-subunit M2-M4 interactions are strengthened. Together, these results show that the path CV, decomposed by the M4-related auxiliary CVs, captures the global structural rearrangements of TREK-2 across different regulatory conditions and reveals phosphorylation and membrane stretch as opposing modulators along a common energetic axis. Independent support for the physical relevance of the up- and down-state minima from OneOPES comes from our previous unbiased simulations, in which the up- and down-state ensembles, when projected onto the path CV, populate regions that closely match the respective basins resolved in the FES (Supplementary Fig. 5a, b).

### Energetic coupling between the pCt and SF defines the thermodynamic basis of TREK-2* gating

**Fig. 4:**
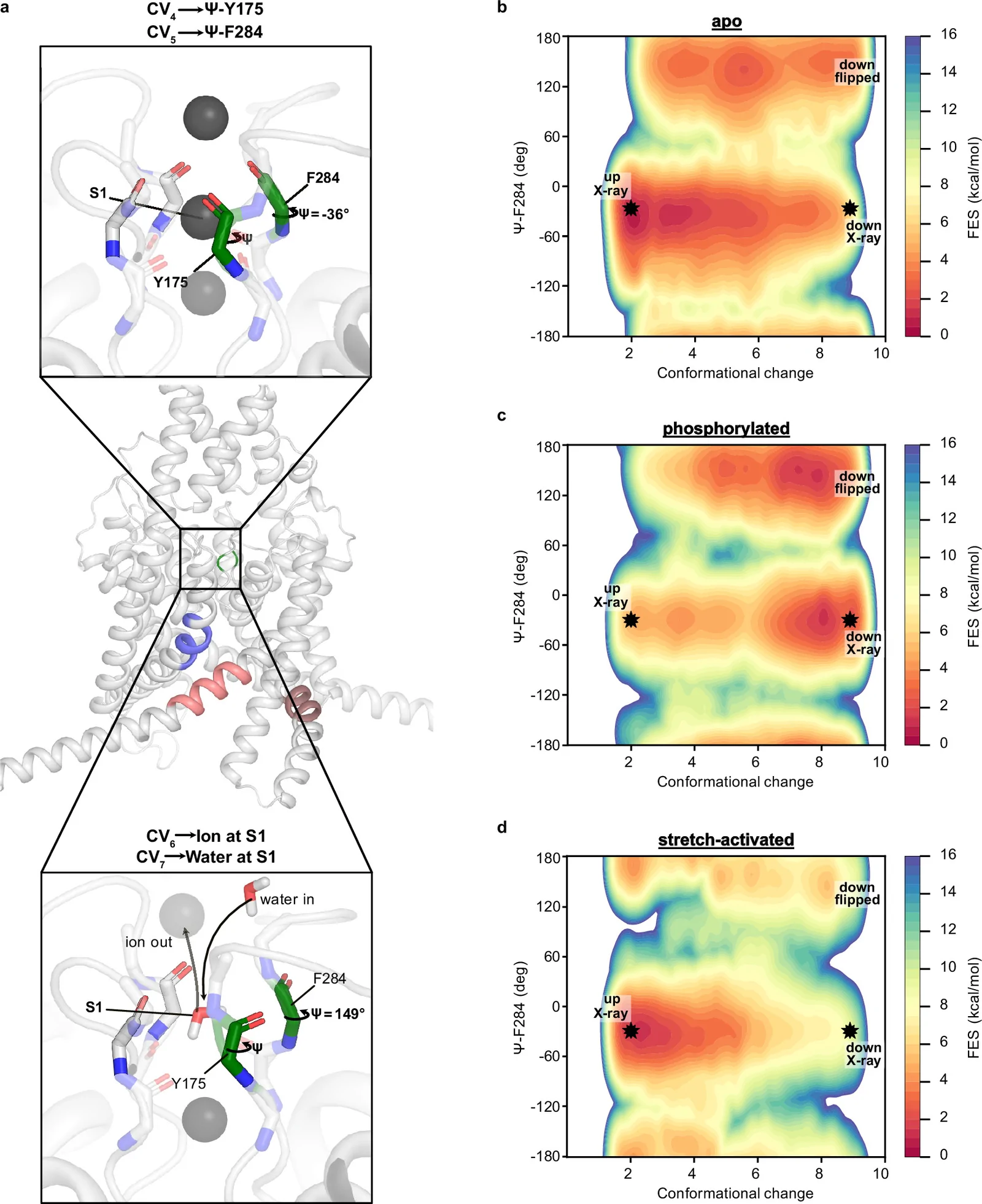
Auxiliary CVs describing SF dynamics suggest the energetic coupling between pCt/M4 movement and gating-associated SF changes in the TREK-2* channel. **a** Schematic representation of the two auxiliary CVs used to describe the SF stability in canonical and carbonyl-flipped conformations. The canonical conformation is defined by the ψ dihedral of F284 adopting its ion-coordinating crystallographic geometry (ψ ≈ −36°), whereas the initial SF rearrangement is characterized by a flipped orientation of the F284 backbone carbonyl away from the S1 ion-binding site (ψ ≈ +149°). Two additional auxiliary CVs describing K^+^ and water occupancy at the S1 ion-binding site were further included to facilitate sampling of SF transitions and the associated rearrangements of SF residues. **b** 2D FES projected along the path CV and the auxiliary CV describing SF stability for apo, **c** phosphorylated and (**d**) membrane-stretch simulations. The average of three independent replicas for each system were calculated for final 2D FES. Black stars indicate the positions of the crystallographic TREK-2 up- and down-state structures mapped onto these coordinates, based on their measured path CV values and AdA distances. Comparison of panels (**b**–**d**) highlights two features that quantify the coupling between M4–pCt motion and SF gating: the relative free-energy depth of the X-ray (–30°) and flipped (+145°) ψ-F284 conformational basins, and the barrier height between them, both of which vary substantially across the apo, phosphorylated, and membrane-stretch conditions.

After establishing the energetic correspondence between the path CV and dynamics of the M4 helices, we next examined whether the conformational transitions at the M4-pCt are causally linked to the conformational behavior of the SF. As discussed before, the SF represents the narrowest region of the pore and has been proposed to serve as the primary gate controlling ion conduction in TREK channels. Our previous work has shown that subtle dihedral rotations within the SF backbone at the S1 K^+^ binding site (Fig. 4a) can drive TREK channels into a non-conductive state during ion permeation simulations^17^. To capture this transition in energetic terms, we projected the OneOPES FES along the path CV and two auxiliary CVs corresponding to the ψ dihedral angles of residues F284 and Y175 within the SF (Fig. 4a). In the apo simulations, the dominant free-energy basin within the up-state region of the FES corresponds to a conductive SF configuration, where the ψ dihedral angle of F284 remained in their ion-coordinating crystallographic state (Fig. 4b). It should be noted that the functional assignment of the conductance property of the SF is based solely on our previous ion permeation simulations^17^ and is not a direct outcome of the present study. A metastable flipped-SF conformation of F284 was also observed within the up-state region, about 3 kcal/mol higher in free energy. Notably, the barrier separating X-ray-like and flipped SF in the up-state region is large (~10–12 kcal/mol), consistent with the absence of SF flipping in our previous unbiased simulations of the up-state. As the channel transitions toward the down-state, the X-ray-like conductive SF conformation becomes progressively destabilized, while the flipped SF emerges as the thermodynamically favored state. Concurrently, the free-energy barrier separating the two SF conformers decreases to ~6 kcal/mol, such that flipping becomes considerably more accessible. Together, these results support the view that, under basal conditions, TREK-2* dynamically exchanges between conductive X-ray-like and F284 carbonyl-flipped states. We acknowledge that part of the observed co-variation between the path CV and the SF dihedral observables again arises from the geometric consequence of the path CV construction itself, as the up- and down-state crystal structures used to define the path may slightly differ in their SF environment, even though both crystallographic states adopt the same conductive SF conformation.

In addition to F284, we also monitored the ψ dihedral angle of Y175, located on the opposite side of the S1 site. Although Y175 represents an important structural component of the SF, its sampling behavior in our simulations deviated from that observed for F284 Supplementary Fig. 6a). We observed that both SF conformers were thermodynamically accessible within the up-state region of the free-energy landscape, a feature not sampled in our previous unbiased simulations (Supplementary Fig. 5d). This difference likely arises from the limited set of auxiliary CVs used to describe Y175 dynamics. In our previous unbiased simulations, interaction-network analysis suggested that dynamics of Y175 was mediated by additional contacts between the M4 loop and the extracellular (EC) loop in the down state. Since these interactions were not explicitly encoded as CVs in the present OneOPES simulations, the underlying structural determinants governing Y175 dynamics were likely not sampled adequately, limiting the reliability of the resulting thermodynamic description of its backbone conformations.

Upon phosphorylation, the up-state became destabilized, and the phosphorylated channel predominantly occupied the down-state, where SF flipping of F284 was more energetically favored (Fig. 4c). This result provides further energetic evidence that the dynamics of the SF and M4 helices are thermodynamically coupled to one another. Conversely, the stretch-activated system displayed the opposite energetic profile. Under mechanical stretch, the energy landscape collapsed onto a single deep minimum corresponding to the up-state with a stable SF conformation for F284 (Fig. 4d). The barrier separating the stable and F284 backbone-flipped configurations exceeded 14 kcal/mol, making conformational transition substantially less probable than in the apo state. Similar to the apo-state, both the X-ray and backbone-flipped conformation of Y175 remained thermodynamically accessible within the up-state region of the free-energy landscape in the phosphorylated and membrane-stretched simulations (Supplementary Fig. 6b, c).

To place these condition-dependent shifts on a quantitative footing, rather than relying solely on visual inspection of the FES, we integrated the reweighted populations within fixed up- and down-state basins, defined identically for all systems on the two-dimensional FES spanned by the path CV and either the M4–M2 (AdA) distance or the ψ-F284 dihedral (Details see “Methods”: OneOPES simulations). From these populations, we calculated the free-energy difference between the two states, ΔG(down–up), for each independent run (Supplementary Fig. 7a), with the same ordering of conformational equilibria obtained for ψ-F284 (Supplementary Fig. 7b). This analysis shows that the phosphorylated system is the only condition in which the down-state is thermodynamically favored, whereas membrane stretch and A314P both shift the equilibrium strongly toward the up-state. The inter-condition free energy differences ΔΔG(down–up) (apo vs phosphorylated: 31.8 ± 3.2 kJ mol⁻¹; apo vs stretch: −23.0 ± 6.1 kJ mol⁻¹) substantially exceed the run-to-run uncertainty, thereby supporting a quantitative distinction between conditions that extends beyond the qualitative visual comparison of the free-energy landscapes (Supplementary Table 3).

### Disease-mimicking mutation reshapes the TREK-2* energy landscape toward a conductive state

**Fig. 5:**
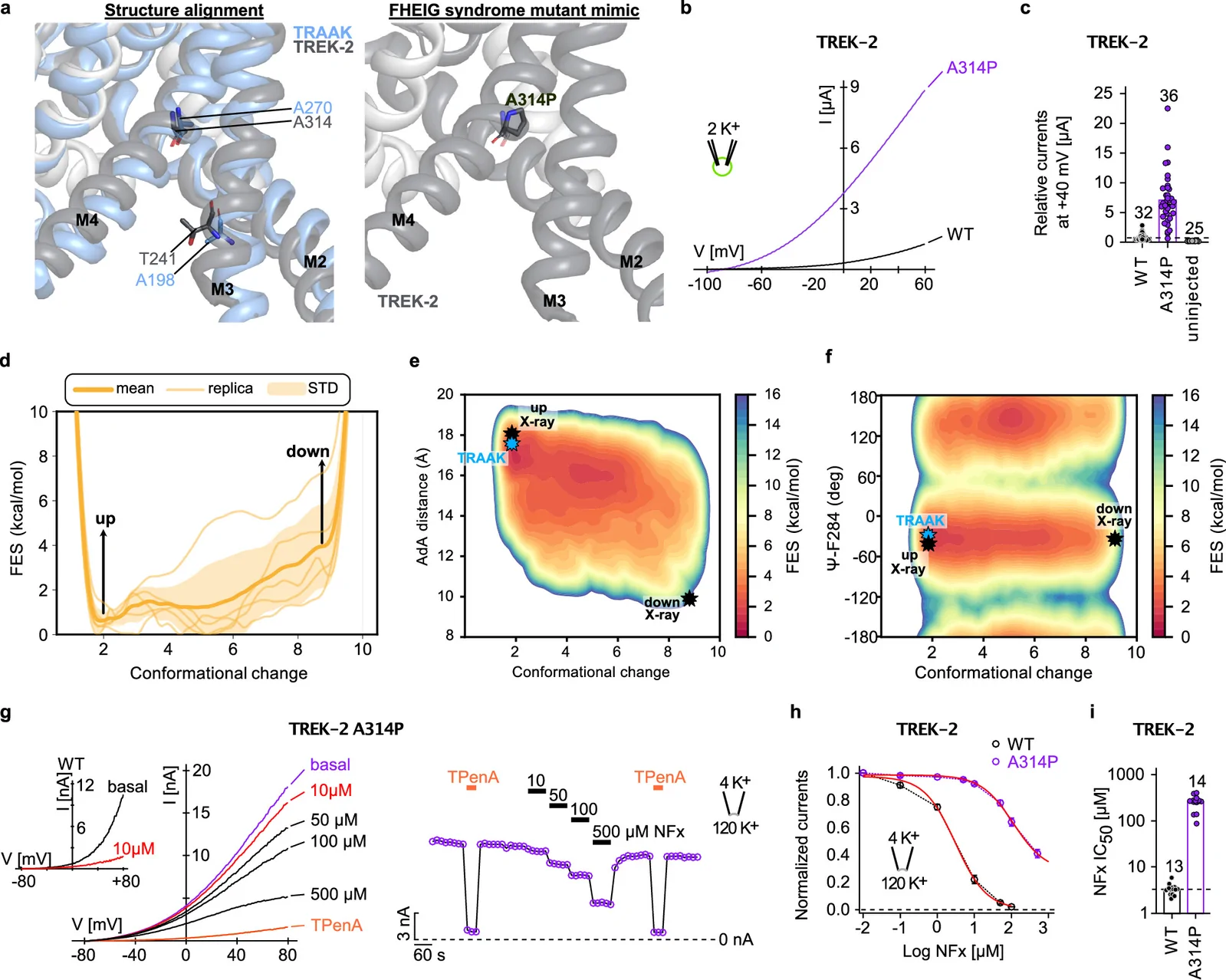
Disease-mimicking mutation stabilized TREK-2* into an up-state conformation. **a** Alignment of two K_2P_ channels, TRAAK (PDB ID: *4WFE*)^22^ and TREK-2 (PDB ID: *4BW5*)^24^ with GoF mutation residues highlighted. **b** Example measurements and (**c**) relative currents measured and analyzed from oocytes expressing TREK-2 WT, A314P mutant channels and uninjected oocytes, respectively in TEVC with extracellular low K^+^ [2 mM] at pH 7.4 at +40 mV. Analyzed electrophysiological data is shown as mean ± s.e.m. from n (number of independent experiments/ replicates) of oocytes expressing WT (*n* = 32), A314P (*n* = 36) and uninjected oocytes (*n* = 25). **d** 1D free energy surface (FES) projected on to the path CV for disease-mutant (A314P) TREK-2* simulations. The solid line represents the mean of five independent simulations. The shaded region corresponds to the standard deviation computed across the five kernel-reweighted free energy profiles at each point. This reflects run-to-run variability and does not include statistical uncertainty from binning. **e** 2D FES projected along the path CV and the auxiliary CV describing M4-M2 distance in the opposite subunit (BdA distance). **f** 2D FES projected along the path CV and the auxiliary CV describing SF stability for disease-mutant TREK-2* simulations. An X-ray structure of previously determined TRAAK A270P mutant (PDB ID: *7LJ4*)^44^ was projected onto the 2D FES as blue star. Black stars indicate the positions of the crystallographic WT TREK-2 up- and down-state structures mapped onto these coordinates, based on their measured path CV values and AdA distances. **g** Example measurements of WT TREK-2 and A314P mutant channels in an asymmetric K^+^ gradient at pH 7.4 in a voltage range from −80 to +80 mV showing the dose-dependent inhibition with the indicated concentrations NFx (left) and the time course of block analyzed at +40 mV (right). **h** Dose-response curves of NFx inhibition for WT and mutant TREK-2 channels analyzed from recordings as in (**g**). Analyzed data is shown as mean ± s.e.m. from n (number of independent experiments/ replicates). **i** NFx IC_50_ values from dose-response curves as in (**h**) for WT (*n* = 13) and A314P mutant TREK-2 K_2P_ channels (*n* = 14). For all electrophysiological experiments at least 3 different batches of oocytes have been used.

To investigate how a disease-associated mutation reshapes the conformational landscape of TREK-2* and thereby leads to channel dysfunction, we performed an additional series of free-energy simulations with OneOPES. As a proof of principle, we selected a pathogenic GoF mutation in TRAAK (A270P) identified in patients with FHEIG syndrome (Fig. 5a) and introduced the homologous mutation (A314P) into the M4 helix of TREK-2*^21^. Currents recorded from oocytes expressing the mutant TREK-2 channel were increased by 9-fold compared to WT (Fig. 5b, c). In parallel, we performed five independent OneOPES simulation runs, each compromising 8 replicates of 1 μs, to evaluate the energetic and structural consequences of the mutation.

Both 1D and 2D FES projected along the path CV and the auxiliary CVs describing M4 dynamics revealed a distinct energetic profile compared to the WT system (Fig. 5d, e and Supplementary Fig. 4d). In contrast to the shallow, more broadly distributed landscape of the apo channel, the mutant exhibited a more localized energetic basin centered on the up-state conformation. Similar to the membrane-stretched system, the 2D FES shows the M4 helix drawn toward the M2 helix of the opposing subunit (low BdA value), accompanied by marked reduction in conformational freedom. However, compared with the membrane-stretched condition, the TREK-2* A314P simulations exhibit broader fluctuations within the up-state basin, indicating increased conformational flexibility of the M4-pCt region (Figs. 3d, 5e), which is further reflected in the larger run-to-run variability observed across independent simulations (Supplementary Fig. 7a). Direct structural comparison provides further insight into the up-state stabilization in the mutant. A representative structure extracted from the A314P up-state minimum shows that the proline substitution introduces a kink in M4 ( ~ 66°; Supplementary Fig. 8 and Supplementary Table 4), which laterally displaces the pCt–M4 interface toward the opposing M2 helix and brings L320 (M4) into close contact with P198 (M2) of the neighboring subunit (3.7–4.1 Å). This geometry is very similar to the experimentally determined TRAAK A270P mutant structure^44^ and contrasts with the TRAAK WT down-state, in which the corresponding contact is longer (4.6–7.1 Å). Notably, this structural effect is opposite to the mechanism we previously proposed for phosphorylation-induced inhibition, in which phosphorylation at S331 disrupts the inter-subunit hydrophobic interactions between S331 and F202 at M2.

To experimentally probe the down- and up-state equilibrium of TREK-2, we used the state-dependent inhibitor norfluoxetine (NFx), which binds within the side-fenestrations that are accessible only in the down-state and thereby inhibits channel activity^24,45,46^. NFx was applied at increasing concentrations to the cytoplasmic side of excised membrane patches expressing WT or mutant (A314P) TREK-2. The mutation shifted the IC₅₀ for NFx inhibition from 3.4 ± 0.3 µM (WT) to 256.7 ± 23.4 µM (A314P), consistent with a pronounced shift of the conformational equilibrium toward the NFx-insensitive up-state (Fig. 5g–i).

We next examined whether this stabilization of the up-state affected the energetics of the SF. Projections of the FESs along the path CV and the auxiliary CV describing the ψ dihedral angle of F284 revealed, on average, a comparable population distribution between the X-ray-like and backbone-flipped conformations in the A314P mutant and WT channel (Fig. 5f and Supplementary Fig. 7b). However, we observe substantially larger variation in the relative populations across independent simulation replicas in the mutant system, indicating reduced convergence of the free-energy estimates (Supplementary Fig. 7b). This variability limits our ability to draw conclusions regarding the precise energetic impact of the mutation of the equilibrium between the two SF conformations.

## Discussion

TREK channels are polymodal K^+^ channels regulated by a diverse range of external stimuli^2,3,6,7^, and their dysfunction has been implicated in multiple neurological disorders^13,47–49^. In our previous work, we showed that the crystallographic up- and down-state conformations of TREK-2 channel display markedly different ion conductivities in the simulations^17^. These differences were associated with persistent conformational rearrangements at the extracellular side of the SF, particularly involving residues that stabilize the S1 ion binding site. Furthermore, network analysis of the MD trajectories, based on pairwise residue interactions that differed between two states, suggested two potential allosteric coupling pathways linking the pCt/M4 region to the SF: (i) M4  →  M4 loop/EC loop → SF; (ii) M4/M2  →  P2  →  SF. However, as only a few spontaneous transitions between the up- and down-states were observed in these unbiased simulations, the sampling was insufficient to quantitatively characterize the conformational equilibrium or determine how external stimuli reshape the underlying free energy landscape.

**Fig. 6:**
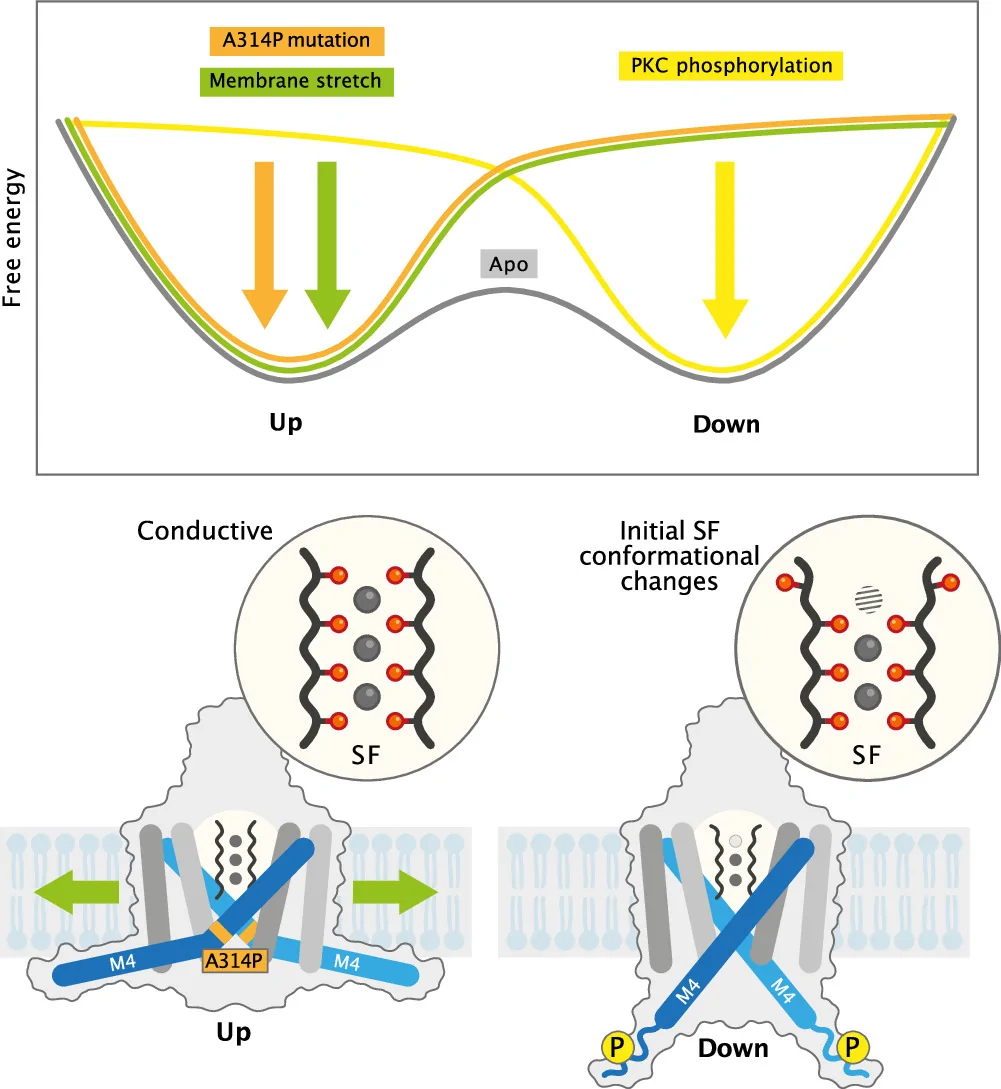
Energetic modulation of TREK-2* gating by phosphorylation, membrane stretch, and disease-associated mutation. Schematic representation of the free-energy landscapes describing TREK-2* conformational equilibria. In the apo state, the channel exhibits a bimodal energy profile with comparable up- and down-state minima. Posttranslational phosphorylation at the PKC site shifts the equilibrium toward the non-conductive down-state, whereas membrane stretch and the A314P mutation both favor stabilization of the conductive up-state. Lower panels illustrate the corresponding structural and functional outcomes: upward displacement of the M4 helix and a stable, ion-conductive SF under activating conditions, versus a downward M4 conformation associated with initial SF conformational changes linked to gating in the inhibited, phosphorylated state.

In the present work, we revisited this problem using the enhanced sampling framework OneOPES to calculate the FES associated with conformational changes in both the pCt/M4 region and the upper part of the SF. We show that, although these regulatory inputs act at distinct structural sites−phosphorylation at the pCt, mechanogating via membrane-pCt/M4 coupling, and the disease-associated mutation within the M4 helix—they converge on a shared energetic network that couples the dynamics in M4 helix, pCt, and the upper region of the SF (Fig. 6). In the apo system, free-energy landscapes were relatively shallow, allowing dynamic exchange between up- and down-state as well as a range of intermediate conformations. Within the up-state basin, the previously assigned conductive crystallographic SF configuration^17^ represents the dominant thermodynamic minimum, whereas a secondary carbonyl-flipped conformer of F284 is separated by a substantial free-energy barrier. As a result, this flipped state was not sampled in our previous unbiased simulations of up-state on the μs timescale, but became resolved in the present OneOPES simulations. Phosphorylation at the pCt shifted the equilibrium towards the down-state and increased the population of flipped SF configurations. This finding aligns with our previous computational observations showing that phosphorylation promotes conformational changes at the SF and inhibits ion conduction^17^. In contrast, application of membrane stretch stabilizes the up-state, and the channel predominantly sampled conductive SF configurations throughout most of the simulations.

In this study, we obtained a strong co-variation between the global M4-pCt displacement and the stability of F284 backbone at the S1 site. In contrast, the energetic description of the Y175 deviates from our previous unbiased simulations, which we attribute to the absence of additional CVs capturing conformational changes in the M4 loop and extracellular loop that may contribute to Y175 rearrangements, as suggested by our earlier unbiased MD simulations. Furthermore, unlike our unbiased simulations performed using the Computational Electrophysiology framework^17^, all free-energy results presented here correspond to the zero-voltage ensemble. While this difference may in addition, affect the sampling of Y175 conformational transitions, we acknowledge that the absence of transmembrane voltage could, in principle, alter the electrostatic environment of the whole channel and thereby influence channel properties such as K⁺ occupancy within the SF, hydration of the inner cavity, and the dynamics of the highly charged pCt region. However, incorporating an explicit membrane voltage into a replica-exchange enhanced-sampling framework is methodologically challenging, as it violates the equilibrium conditions required for rigorous bias reweighting. For this reason, the OneOPES simulations were performed here in the absence of an applied voltage. Extending these approaches to systematically characterize voltage-dependent modulation of the conformational observables discussed here, for example, using voltage-coupled enhanced-sampling schemes, represents an important direction for future methodological and mechanistic studies.

A further consideration concerns the lipid composition of our simulation systems, which consists exclusively of POPC. This simplification was intentionally adopted to isolate the intrinsic protein-driven energetics of the M4–pCt–SF coupling from the additional complexity of lipid-specific modulation. It has been previously shown that TREK channels are modulated by anionic lipids, most notably PI(4,5)P₂ and other phosphoinositides, as well as by polyunsaturated fatty acids such as arachidonic acid^13,50^. A simple POPC bilayer cannot capture lipid-specific effects on the conformational equilibrium. The free-energy differences reported here should be therefore interpreted as the intrinsic response of the protein to phosphorylation, membrane stretch, and mutation within a simplified model membrane environment. A further consideration concerns the ionic strength of our simulation systems, which was set to 600 mM KCl to maintain direct comparability with our previous unbiased MD simulations of TREK-2^17^, where elevated K⁺ concentrations were employed to accelerate ion conduction under transmembrane voltage. As the pCt contains multiple charged residues, including the phosphoserine modeled here in its doubly deprotonated state ( − 2 charge), elevated ionic strength could in principle influence the computed free-energy landscape through electrostatic screening effects. Nevertheless, the predicted conformational equilibria qualitatively agree with available experimental observations regarding the effects of phosphorylation, membrane tension, and state-dependent inhibitor binding in the mutant systems. Future work could systematically investigate the influence of ionic strength on the computed free-energy surfaces.

A practical aspect of the OneOPES results worth commenting on is the run-to-run variability of the reconstructed free-energy profiles, visible as the standard-deviation envelopes in Fig. 2b–d and Fig. 5d. Some level of variability is expected for replica-exchange enhanced sampling of high-dimensional systems: each independent run explores a slightly different sequence of replica exchanges and accumulates a slightly different bias history, and any low-dimensional CV set, however carefully chosen, leaves residual hidden degrees of freedom that contribute additional dispersion. Reporting variance across three (or, for the A314P system, five) independent runs is therefore a more conservative estimate of reproducibility than block analysis of a single trajectory, but it also produces a larger apparent standard deviation.

Atomistic mechanism of mechano-gating in TREK channels has been a central topic in previous computational studies employing both all-atom and coarse-grained simulations^10,43,51^. Coarse-grained simulations have primarily focused on achieving sufficient sampling of the up-to-down transition in the M4 helix of TRAAK channel by applying surface tension, in line with the “force from lipid” principle of mechanosensitivity^51^. In parallel, several all-atom MD studies on TREK-2 reported SF conformational rearrangements and associated changes in ion occupancy within the filter. However, these unbiased MD simulations, limited by their accessible timescales, did not establish an energetic link between the SF dynamics and conformational transitions in the M4 helix. We noted that the specific SF rearrangements reported in these studies differ. While the MD study by J. T. Brennecke et. al proposed conformational changes at the S3 site^43^, P. Aryal and we independently observed up- and down-state-dependent rearrangements at the S0 and S1 sites^10,17^. We do not view these results as contradictory, as our previous MD study on non-canonical voltage gating suggested that conformational changes at the S1 and S3 sites in TREK channels are coupled to each other during the gating, while we considered the conformational changes at the extracellular side represents the initial SF gating event^52^. Nonetheless, our OneOPES simulation results were unable to resolve the full SF gating procedure. A posteriori analysis of the reweighted ensembles showed that S2-S4 ion occupancies and the Y175-Y175 distance do not vary substantially across the different simulation conditions (Supplementary Fig. 9). This most likely reflects the fact that these additional SF descriptors were not explicitly biased during the simulations, whereas biasing only the local rearrangements at the S1 binding site is insufficient to drive the full range of SF dynamics at the current timescales.

A disease-mimicking mutation (A314P) in TREK-2* offered an additional perspective on the underlying energetic landscape. Substitution of the conserved A314 residue with proline within the M4 helix, analogous to a GOF mutation previously reported in TRAAK channels^21^, resulted in a pronounced energetic basin corresponding to the up-state, while the down-state was energetically less accessible across all replicas. Consistent with the experimentally determined structure of the corresponding TRAAK A270P mutant, in which the equivalent proline substitution alters the local geometry of the M4 helix^44^, the A314P substitution shifts the energetic balance toward an expanded, up-state geometry that resembles the mechanically stretched condition (Supplementary Fig. 7a and Supplementary Table 3). These findings further suggest that our computational framework could be extended to other de novo TRAAK mutations associated with FHEIG phenotypes. Many of these mutations cluster within the intracellular M2-M3-M4 interaction network, which is expected to influence the up-to-down transition^44^. However, due to the high computational cost and the substantial variability observed across individual simulation runs of the A314P mutant, which complicates both convergence and interpretation, we are currently limited in systematically assessing how these different mutations influence the up-to-down transition of M4 and the associated conformational changes in the SF. More systematic investigations, involving substantially longer sampling times and/or the inclusion of additional CVs will therefore be required in future studies.

In this work, together with our previous unbiased MD simulations, the free-energy landscapes obtained from OneOPES provide a more coherent energetic framework for understanding how chemical, mechanical, and mutational perturbations modulate TREK-2 function through shared structural pathways and define the channel’s gating landscape. These findings are consistent with a very recent study from some of us, in which electrophysiology measurements suggested that diverse physiological stimuli activate TREK/TRAAK channels through a common mechanistic pathway^53^. Importantly, the unbiased MD simulations are essential for identifying the complex interaction network coupling the pCt/M4 region to the SF and for defining the key structural descriptors that best characterize the essential conformational transition. In contrast, the state-of-the-art enhanced sampling approach applied here is crucial for quantifying the free-energy changes associated with these transitions and for determining how they are reshaped by different physiological perturbations. Together, these results highlight the complementary strengths of unbiased and enhanced sampling MD approaches in dissecting regulatory mechanisms in complex biological systems and underscore the importance of their integration. Our study, therefore, not only provides further theoretical support for the coupling between the SF gate and the lower bundle helices in TREK channels, but also establishes a framework for quantitatively linking conformational dynamics to energetics in polymodally regulated ion channels.

## Methods

### Ethical statement

The investigation conforms to the guide for the Care and Use of laboratory Animals (NIH Publication 85-23) and were approved by the local ethics commission of the Ministerium für Landwirtschaft, ländliche Räume, Europa und Verbraucherschutz (IX 555–106759/2023 (33-6/23 V)) and permitted by the department for animal experimentation to Dr. M. Schewe.

### MD-based system preparation

The apo-, phosphorylated, stretch and disease-mutant structures of TREK-2 were generated starting from up- (PDB ID: *4BW5*)^24^ and down-state (PDB ID: *4XDJ*)^24^ conformations, respectively. The missing pCt region in each subunit was manually extended using the PyMOL builder tool^54^. A total of 19 residues (333–351: TKEEVGEIKAHAAEWKANV), corresponding to the pCt extension of the TREK-2* construct used in our previous study^17,55^, were modeled in an α-helical conformation. The choice of this initial starting conformation is supported by AlphaFold3^56^ predictions of the full-length TREK-2 structure (Supplementary Fig. 10), in which the pCt region is predicted to adopt a helical structure with pLDDT scores ranging from 70 to 90. Additionally, missing loop segments (residues 149–154 and 229–235) were modeled using SWISS-MODEL^57^.

We performed MD simulations with AMBER99sb^58^ force field. The insertion of the TREK-2* channel into a POPC was performed with the GROMACS internal embedding function. The concentration of KCl was 600 mM in simulations, in consistent with our previous unbiased MD simulations under transmembrane voltages^17^, where higher ionic concentration compared to physiological condition was applied to accelerate ion conduction rate. Improved ion parameters^59^ and lipid parameters^60^ were employed in the simulations. The SPC/E water model^61^ was used in simulations.

All MD simulations were performed with the GROMACS software (2022.5)^62^ patched with PLUMED 2.8.5^63^. Short-ranged electrostatics and van der Waals interactions were truncated at 1.0 nm. Long-range electrostatics were calculated with the Particle-Mesh Ewald summation^64^. Temperature and pressure coupling were treated with the V-rescale^65^ scheme and the Parrinello-Rahman barostate^66^, respectively. The temperature was set to 300 K and the pressure to 1 bar. Fluctuations of the periodic cell were only allowed in the z-direction, normal to the membrane surface, keeping the density of the membrane unchanged. Verlet cut-off scheme was used for neighbor searching and pair interactions^67^. All bonds were constrained with the Linear Constraint Solver (LINCS) algorithm^68^.

After the channel was embedded into POPC with ions, the system was energy minimized and equilibrated. The energy minimization was done with GROMACS “steepest descent” algorithm with a maximum energy value of 100 kJ mol^−1^ to make sure to minimize the energy of the system as much as possible. After the minimization, the system was equilibrated for 10 ns with a position restraint by a force constant of 1000 kJ mol^−1^ nm^−2^ in x, y, and z-direction on the backbone atoms of the protein followed by 20 ns without position restraints on the backbone atoms of the protein (Supplementary Fig. 11). During the complete equilibration, the isothermal–isobaric (NPT) ensemble was used.

The simulations were initiated with a four-ion configuration (S1–S4) in the SF for all setups, consistent with the ion configuration in the X-ray structure of the TREK channel in the up-state (PDB ID: *4BW5*)^24^.

For the in-silico preparation of the phosphorylated serine residue in the TREK-2* in the up-state structure, the hydrogen of the hydroxyl group was removed, and a phosphate group was added to the oxygen using the builder option of PyMOL. The hydrogen atom in the phosphate group was removed to maintain the phosphoserine at a −2 charge. Additional force field parameters for phosphorylated serine were adopted from previous studies^68^.

To investigate the stretch-induced changes in TREK-2*, the bilayer-plane (xy-plane) pressure was set to −50 bar (lateral tension), while the pressure along the bilayer normal (z) direction was kept at +1 bar and the temperature at 300 K. Across a ~ 4 nm-thick POPC bilayer, the applied lateral pressure corresponds to an effective surface tension of γ = d × (P_N_ – P_L_) ≈ 4 nm × 51 bar ≈20 mN m⁻¹, consistent with values used in previous all-atom MD studies of mechanosensitive K2P channels^10,43^. For comparison, excised-patch experiments carry a basal tension of ~0.5–4 mN m⁻¹ from lipid–glass adhesion and reach lytic values of ~12 mN m⁻¹ under pressurization, with TRAAK and TREK channels activating across this range up to saturation near 12 mN m⁻¹^6,22^. The simulated tension is supra-saturating, but is the conventional in-silico activation condition used for this family, required to drive the conformational transition within μs timescales accessible to atomistic simulation. To monitor the response of the bilayer to the applied tension, the volume of the POPC bilayer and the center-of-mass (COM) distance between the head groups of the two leaflets were tracked over the course of the simulations (Supplementary Figs. 12, 13). The remainder of the protocol was identical to that of the apo system.

For the in-silico preparation of the disease-related mutant in the TREK-2* in up-state structure, the mutation of the Ala314 to Pro was performed using the mutagenesis tool of PyMOL^54^. Same protocol was followed for the rest of the preparation of the simulation system similar to the apo system.

### OneOPES simulations

We adopted a CV-based enhanced sampling method to investigate the energetic conformational landscape of apo-, phosphorylated, membrane stretch, and disease-mutant TREK-2* structures. We employed “On-the-fly probability enhanced sampling” (OPES) algorithm in its “Explore” flavor^69^. To perform complete transitions between up- and down-state structures of TREK-2, we employed a combination of CVs. The main CV was a path CV (pCV)^70^ describing the transition of Cα atoms between the crystallographic up- and down-state structures of TREK-2. Intermediate structures for the path were generated using the PyMOL morph function, yielding 10 protein conformations (MODELS) equally spaced in RMSD between the up- (MODEL = 0) and down-state (MODEL = 9). Flexible regions were excluded from the alignment procedure (Fig. 2a) and used for conformational path calculation. While this CV is sufficient to drive the overall motion of the channel, we used additional auxiliary CVs to govern sampling of slow, localized gating motions, as well as ions and water occupancies in the selectivity filter:

- **CV**_**2**_: the distance between M2 and M4 of the same subunit. To describe the CV we selected center of mass of all Cα atoms of residues from D144 to F150 for M2 as first point and center of mass of all Cα atoms of residues from A253 to L262 for M4 as second point.
- **CV**_**3**_: the distance between M2 and M4 of the opposite subunits. To describe the CV we selected center of mass of all Cα atoms of residues from Y127 to P133 for M2 as first point and center of mass of all Cα atoms of residues from A253 to L262 for M4 as second point.
- **CV**_**4**_: the Ψ dihedral angle of Y175 residue located near S1 ion binding site.
- **CV**_**5**_: the Ψ dihedral angle of F284 residue located near S1 ion binding site.
- **CV**_**6**_: the presence of potassium ions at the S1 ion binding site. To describe the CV we used coordination numbers (continuous contacts) between potassium ions and center of S1 ion binding site.
- **CV**_**7**_**:** the presence of water molecules at the S1 ion binding site. To describe the CV we used coordination numbers (continuous contacts) between water molecules and center of S1 ion binding site.

To ensure thorough sampling and reliable convergence of the FES, we adopted the OneOPES replica-exchange setup comprising eight trajectories (Supplementary Table 2). Replica 0 acted as the convergence monitor, while the remaining seven replicas (1–7) were used to enhance exploration. All simulations employed OPES Explore on a defined set of collective variables, with the exploratory replicas progressively heated—up to 335 K—using the OPES Expanded (MultiT) scheme to facilitate sampling across hidden degrees of freedom^71^. Box dimensions and salt concentration for each system kept same (Supplementary Table 5).

In all eight replicas, an OPES_METAD_EXPLORE bias was applied to the path progress coordinate s using a kernel width σ = 0.05 and an estimated barrier of 100 kJ mol⁻¹, with kernels deposited every 50,000 MD steps. This common bias drives the global up-to-down transition consistently across all replicas and ensures that path-CV sampling is shared throughout the replica ladder. Following the OneOPES design, replicas 1–6 additionally biased selected auxiliary collective variables describing slow orthogonal motions, using independent OPES_METAD_EXPLORE instances with a reduced barrier of 5 kJ mol⁻¹ and kernels deposited every 100,000 MD steps. The eighth replica additionally implemented a multi-thermal expansion (OPES_EXPANDED with ECV_MULTITHERMAL) of the potential energy, sampling temperatures between 300 K and 335 K in 40 discrete steps. This replica accelerates the crossing of enthalpic barriers without modifying the thermostat temperature of the thermodynamic ensemble of interest, while reweighting recovers the unbiased canonical distribution at 300 K.

To prevent unphysical excursions in CV space, harmonic wall potentials were applied throughout the simulations. An upper wall on the path distance-from-path coordinate z (at z = 0.2; force constant 2500 kJ mol⁻¹) restrained orthogonal deviations from the reference path. Lower and upper walls on s (at 1.8 and 9.0; 5000 kJ mol⁻¹) restricted sampling to the physical range between the endpoint states. Additional walls bounded the SF ψ dihedrals of Y175 and F284 (between −1.25 and +0.5 rad; 2000 kJ mol⁻¹), the M2–M4 distances (within their physically meaningful range; 2000 kJ mol⁻¹), and the S1 ion/water coordination numbers (lower wall on K⁺ occupancy at 0.15, upper wall on water occupancy at 2.0; 2000 kJ mol⁻¹), preventing the channel from sampling configurations far outside the conformational basins of interest.

A representative structure was extracted from the A314P up-state free-energy minimum. The M4 kink angle was defined as the angle between the vector from the Cα center of mass of the five residues preceding the substituted position to its Cα, and the vector from that Cα to the Cα center of mass of the five following residues. The M4–M2 interface distance was the minimum side-chain heavy-atom distance between L320 (M4) and P198 (M2) of the opposing subunit. The same definitions were applied to the TRAAK A270P (PDB *7LJ4*) and down-state TRAAK (PDB *4WFF*) structures using the equivalent residues (A270, L276, P155), after superposition on the conserved pore domain.

All trajectories were analyzed with GROMACS tools together with PLUMED driver option^63^ and Python using MDAnalysis^72^ together with NumPy^73^ matplotlib^74^ and SciPy^75^. Distances, dihedral angles, coordination numbers and biases were calculated with PLUMED driver.

To calculate 1D and 2D free energy surfaces (FES), OPES biases were reweighted using weighted kernel density estimation. First 100 ns of each independent replica were skipped during FES calculations and considered as the equilibration of the system.

Molecular visualizations were rendered using PyMOL^54^. Figures including Fig. 3a, Fig. 4a and Fig. 6 were generated using Affinity Designer.

### Basin-integrated free-energy differences

To quantify the condition-dependent thermodynamic bias between the up- and down-states beyond visual comparison of the surfaces, we computed the free-energy difference between the two states, ΔG(down–up), by direct integration of the reweighted populations within fixed basins. For each system, this was done on the two-dimensional reweighted free-energy surfaces F(s, q), where s is the path CV and q is either the intra-subunit M4–M2 distance (AdA), describing the global up–down conformational change, or the ψ-F284 dihedral, describing selectivity-filter stability. The surfaces were obtained from the kernel-density reweighting of the OneOPES ensemble on a regular grid, with the free energy referenced to its global minimum.

Up- and down-state basins were defined as fixed rectangular regions in (s, q), anchored to the projected positions of the crystallographic up- and down-state structures and held identical across all four systems, so that the comparison reflects genuine differences between the landscapes rather than differences in basin definition. Along the AdA axis, the up-state basin was taken as 1.85 <s < 3.5 with 16.0 <AdA <18.5, and the down-state basin as 7.5 <s < 8.9 with 8.0 <AdA <12.0. Along the ψ-F284 axis, the up-state basin was taken as 1.85 <s < 3.5 with −60° <ψ-F284 <−10° (stable, conductive SF), and the down-state basin as 7.5 <s < 8.9 with 120° <ψ-F284 < 180° (flipped SF). The intervening transition region was excluded from both basins to avoid including the state populations with intermediate configurations.

The free energy of each basin was obtained by Boltzmann integration of the reweighted density over the basin, F_basin = −k_B_T ln Σ_{i∈basin} exp( − F_i/k_B_T), with k_B_T = 2.494 kJ mol⁻¹ at T = 300 K, and the state free-energy difference computed as ΔG(down–up) = F_down − F_up. With this convention, a positive ΔG indicates that the up-state is thermodynamically favored, and a negative ΔG that the down-state is favored.

ΔG(down–up) was evaluated independently for each of the three (five, for the A314P system) OneOPES runs and is reported as the mean ± standard error of the mean across the independent runs. Differences between conditions, ΔΔG = ΔGᵢ − ΔGⱼ, are reported with uncertainties propagated from the per-condition standard errors as σ(ΔΔG) = √(SEᵢ² + SEⱼ²), and the corresponding separation expressed as |ΔΔG | /σ(ΔΔG). Given the small number of independent enhanced-sampling runs, we did not apply formal significance tests. Instead, the magnitude of each inter-condition difference is assessed relative to its propagated run-to-run uncertainty. All energies are reported in kJ mol⁻¹.

### Molecular biology and construct preparation

In this study, the coding sequence of human TREK-2 (K_2P_10.1, *KCNK10*, GenBank accession number: NM_021161.5) was used. For TREK-2 channel expressed in *Xenopus* oocytes, the coding sequence was subcloned into the oocyte expression vector pFAW. The A314P channel mutant was obtained by site-directed mutagenesis following the Quikchange method with custom oligonucleotides and verified by sequencing. Plasmid DNA was linearized with MluI and cRNA synthesized in vitro using the T7 HighYield^®^ T7 ARCA mRNA Synthesis Kit (Jena Bioscience, Germany).

### Animals and oocyte preparation

For this study, three female *Xenopus laevis* claw frogs were used to isolate oocytes. The investigation conforms to the guide for the Care and Use of Laboratory Animals (NIH Publication 85-23) and was approved by the local ethics commission of the Ministerium für Landwirtschaft, ländliche Räume, Europa und Verbraucherschutz (IX 555–106759/2023 (33-6/23 V)) and permitted by the department for animal experimentation to Dr. M. Schewe.

Ovarian lobes were obtained from frogs anesthetized with tricaine. Lobes were treated with type II collagenase (Sigma-Aldrich/Merck, Germany) in OR2 solution containing (in mM): 82.5 NaCl, 2 KCl, 1 MgCl_2_, 5 HEPES (pH 7.4 adjusted with (NaOH/HCl) for 2 h. Isolated oocytes were stored at 16.9 °C in a solution containing (in mM): 54 NaCl, 30 KCl, 2.4 NaHCO_3_, 0.82 MgSO_4_ × 7 H_2_O, 0.41 CaCl_2_, 0.33 Ca(NO_3_)_2_ × 4 H_2_O and 7.5 TRIS (pH 7.4 adjusted with NaOH/HCl) and were manually defolliculated prior injection with cRNA.

### Two-electrode voltage-clamp (TEVC) recordings of Xenopus oocytes

Oocytes were injected with 2 ng of RNA for WT and mutant channels, respectively and incubated for 2 days at 16.9 °C. Standard TEVC measurements were performed at room temperature (20–22 °C) with a TEC-10CX TEVC system coupled to a HEKA EPC-9 patch clamp amplifier, and PatchMaster software (v2x92). Microelectrodes were fabricated from glass pipettes, back-filled with 3 M KCl, and had a resistance of 0.5–1.5 MΩ. Recordings were performed in ND96 buffer at pH 7.4 (96 mM NaCl, 2 mM KCl, 1 mM MgCl_2_, 1.8 mM CaCl_2_, 5 mM HEPES). Currents were recorded using 800 ms ramp protocols from −100 to +60 mV and a holding potential of −80 mV. All recorded ramps were analyzed at +40 mV.

### Inside-out patch-clamp recordings of Xenopus oocytes

Oocytes were injected with 2 ng of RNA for WT and mutant channels, respectively and incubated for 2–3 days at 16.9 °C in a solution containing (mM): 54 NaCl, 30 KCl, 2.4 NaHCO_3_, 0.82 MgSO_4_ × 7 H_2_O, 0.41 CaCl_2_, 0.33 Ca(NO_3_)_2_ × 4 H_2_O and 7.5 TRIS (pH 7.4 adjusted with NaOH/HCl). Inside-out patch-clamp recordings under voltage-clamp conditions were performed at room temperature (20–22 °C). Patch pipettes were made from thick-walled borosilicate glass GB 200TF-8P (Science Products, Germany), had resistances of 0.2–0.5 MΩ (tip diameter of 10– 25 µm) and filled with a pipette solution (in mM): 4 KCl, 116 NMDG, 10 HEPES and 3.6 CaCl_2_ (pH 7.4 adjusted with KOH/HCl). Intracellular bath solutions and compounds were applied to the cytoplasmic side of excised patches via a gravity flow multi-barrel pipette system. Intracellular solution had the following composition (in mM): 120 KCl, 10 HEPES, 2 EGTA and 1 pyrophosphate (pH adjusted with KOH/HCl). Currents were recorded with an EPC10 amplifier (HEKA electronics, Germany) and sampled at 10 kHz or higher and filtered with 3 kHz (−3 dB) or higher as appropriate for sampling rate. Voltage ramp pulses (−80 mV to +80 mV) were applied from a holding potential of −80 mV for 800 ms with an inter-pulse interval of 9 s and were analyzed at a given voltage of +40 mV. The relative steady-state levels of inhibition for NFx were fitted with the following Hill equation:

1 $$\,\frac{I}{{I}_{0}}=a+\frac{1-a}{1+{\left(\frac{x}{{{IC}}_{50}}\right)}^{h}}$$

where I and I_0_ is the current with and without NFx/TPenA, a is the fraction of initial or residual current, x is the NFx/TPenA concentration, IC_50_ is the concentration at half maximal inhibition, and h is the Hill coefficient. Norfluoxetine (NFx) was purchased from Cayman Chemical (Biozol Diagnostics, Eching, Germany) and tetra-pentyl-ammonium (TPenA) from Sigma-Aldrich (Merck KGaA, Darmstadt, Germany). Compounds were stored as stock solution (50–100 mM) at −80 °C and were diluted in intracellular bath solution to final concentrations prior to each measurement.

Image processing and figure design were done using Affinity Designer, Igor Pro 9 (64 bit) (WaveMetrics, Inc., USA), and Canvas X Draw (Version 20 Build 544) (ACD Systems, Canada) for the Figs. 5b, g, h and 6.

### Reporting summary

Further information on research design is available in the Nature Portfolio Reporting Summary linked to this article.

## Supplementary information

Supplementary Information

Reporting Summary

Transparent Peer Review file

## Data Availability

The MD simulation parameters, initial structures used for the simulations, and the Source Data file have been deposited in Zenodo under the link: (https://zenodo.org/records/21624644) with the (https://doi.org/10.5281/zenodo.18622313). Additional data supporting the findings of this manuscript are available from the corresponding authors upon request.

## Code availability

Molecular dynamics simulation data were generated using the GROMACS software (2022.5)^62^ patched with PLUMED 2.8.5^76^. All trajectories were analyzed with GROMACS tools together with PLUMED driver option and Python using MDAnalysis together with NumPy matplotlib and SciPy.
