# Supplementary material for: Cross-talk of filter gate and lower helices drives polymodal regulation and disease in TREK channels
Source: Nat Commun. 2026 Sep 22;17:11029. doi: 10.1038/s41467-026-77935-w (PMC13687069; doi:10.1038/s41467-026-77935-w)
Supplement: Supplementary file 1 [file 41467_2026_77935_MOESM1_ESM.pdf]

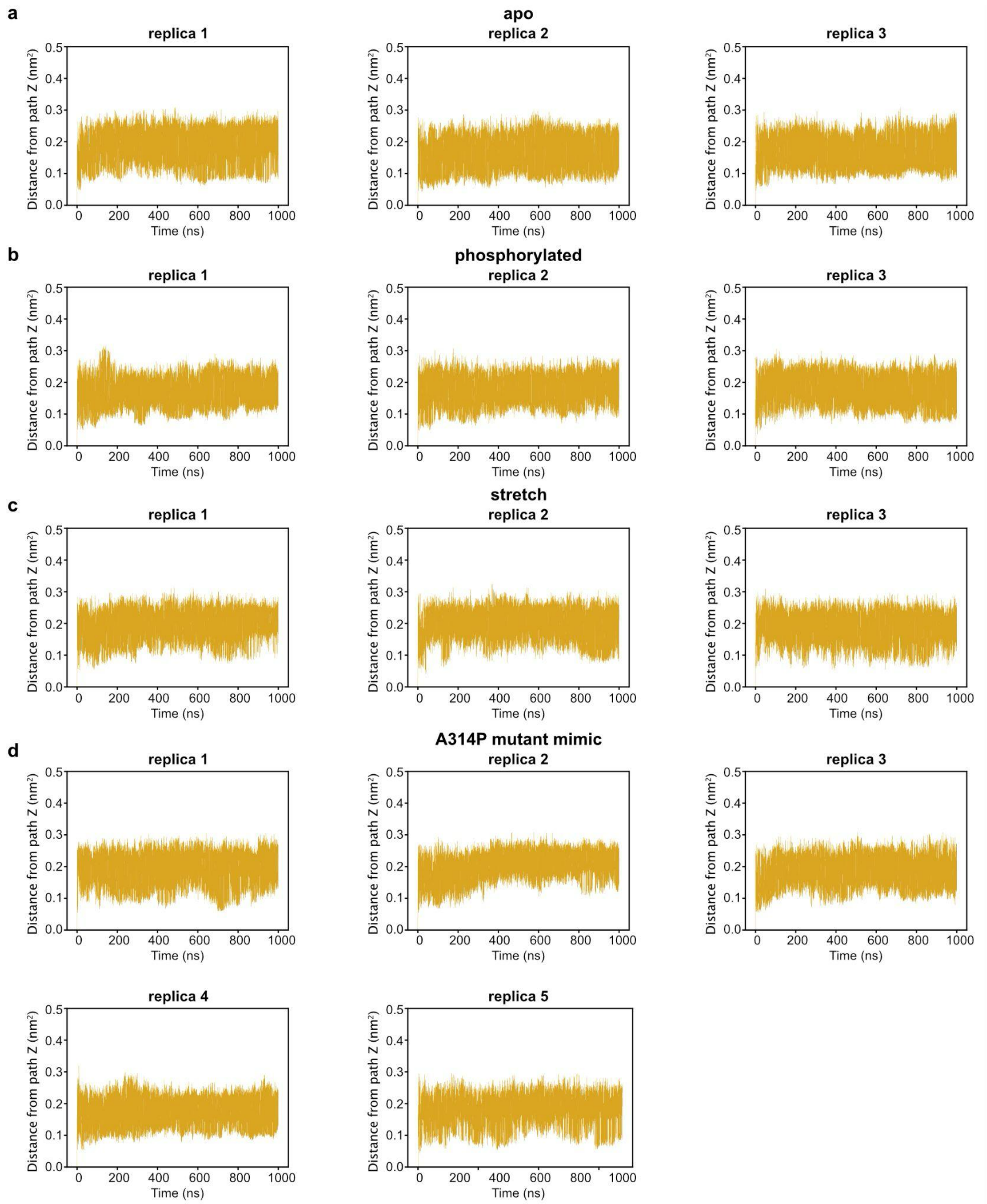

Supplementary Fig. 1. Time evolution of the distance-from-path variable Z. Timewise plots for all replicas across the four systems: **(a)** apo, **(b)** phosphorylated**, (c)** membrane-stretch, and **(d)** A314P mutant. The variable Z represents the RMSD distance from the predefined path collective variable S.

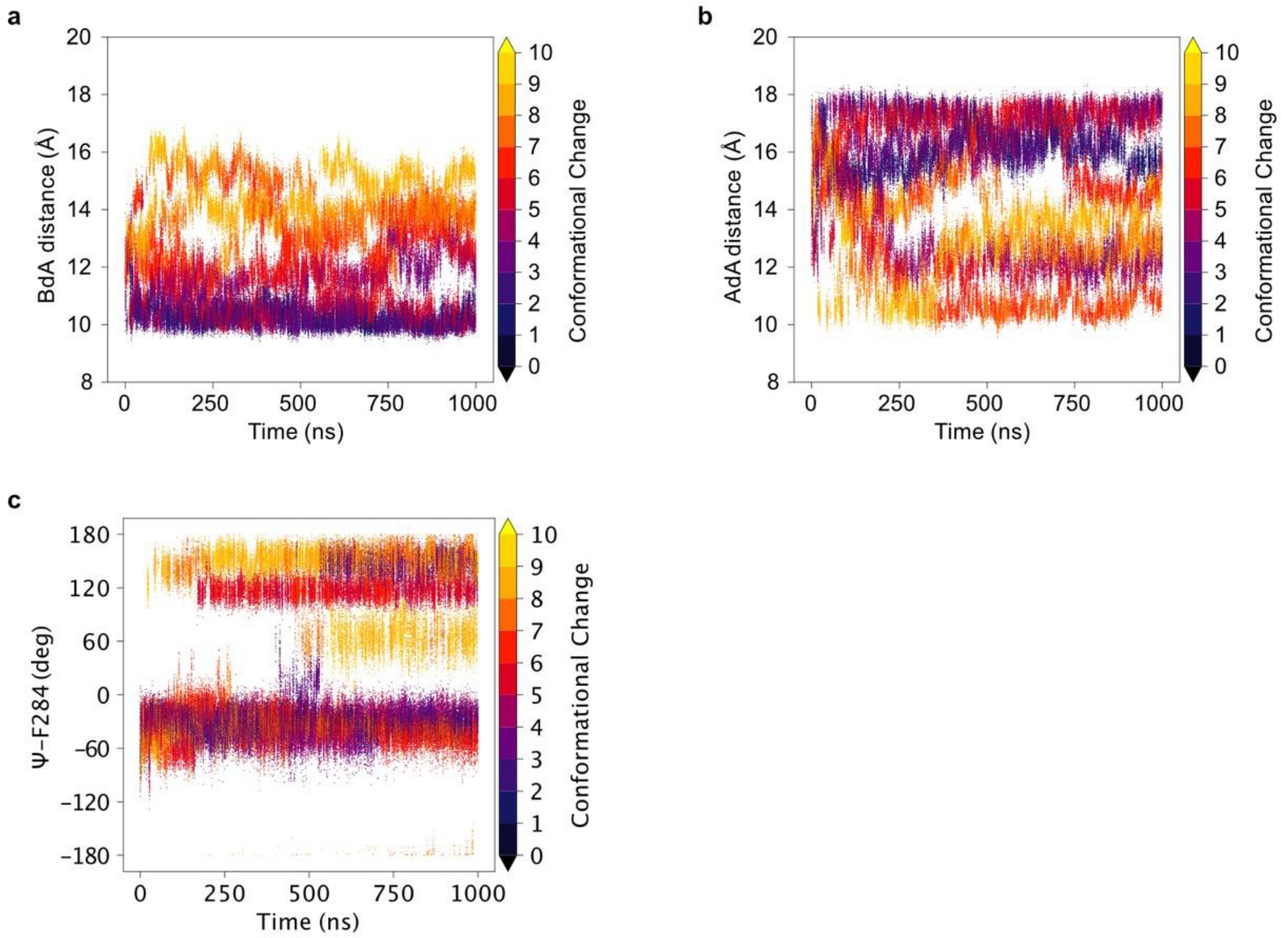

Supplementary Fig. 2. CV sampling in an apo TREK-2 OneOPES simulations. (a,b) Sampling of the M2-M4 distances as a function of time in replica 0, with data points colored according to the path CV. **(c)** Sampling of the carbonyl rearrangement of SF residue F284 as a function of time in replica 0, with data points colored according to the path CV.

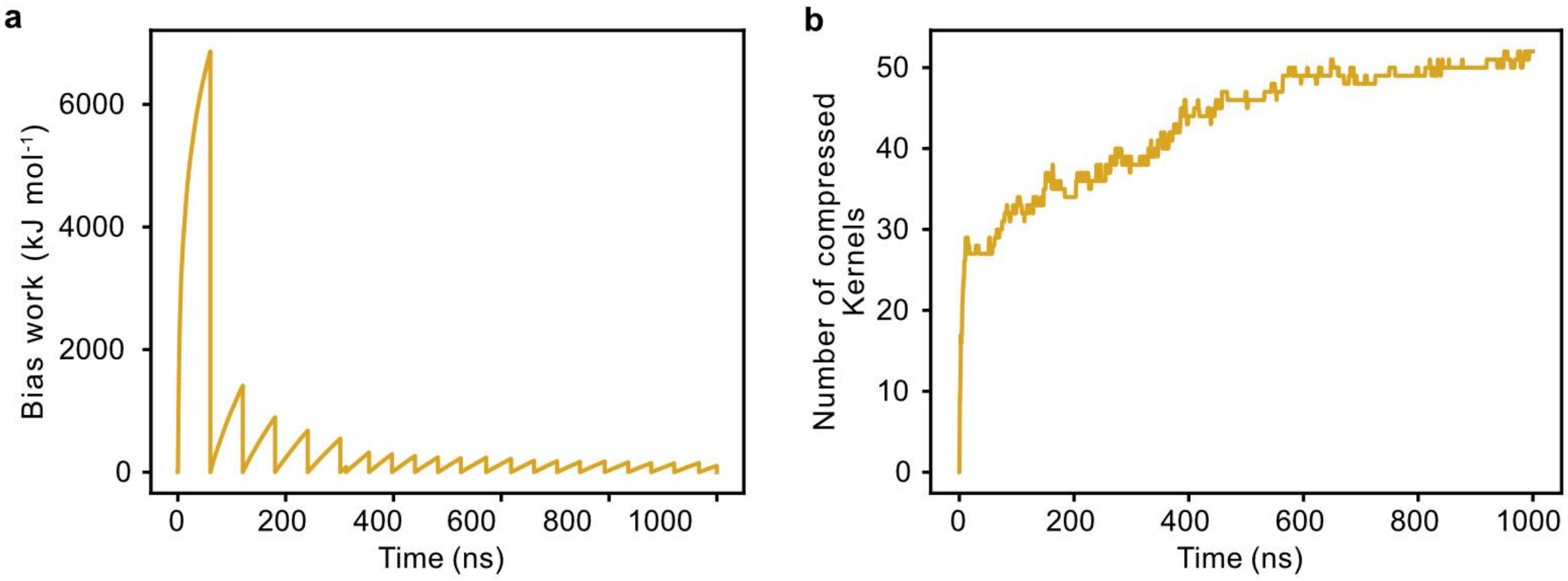

Supplementary Fig. 3. Convergence of the OPES Explore bias in the apo TREK-2* OneOPES simulations. (a) Work performed by the OPES Explore bias applied to the path progress and **(b)** the number of compressed Gaussian kernels used to construct the on-the-fly probability-density estimate, both shown as a function of simulation time for a representative replica. During the initial exploratory regime, the probability density evolves rapidly, leading to substantial bias work and frequent kernel deposition. As sampling progresses, the bias work gradually decreases and the number of deposited kernels approaches a plateau, indicating that the adaptive bias has reached a quasi-stationary regime and that the free-energy estimate is converging.

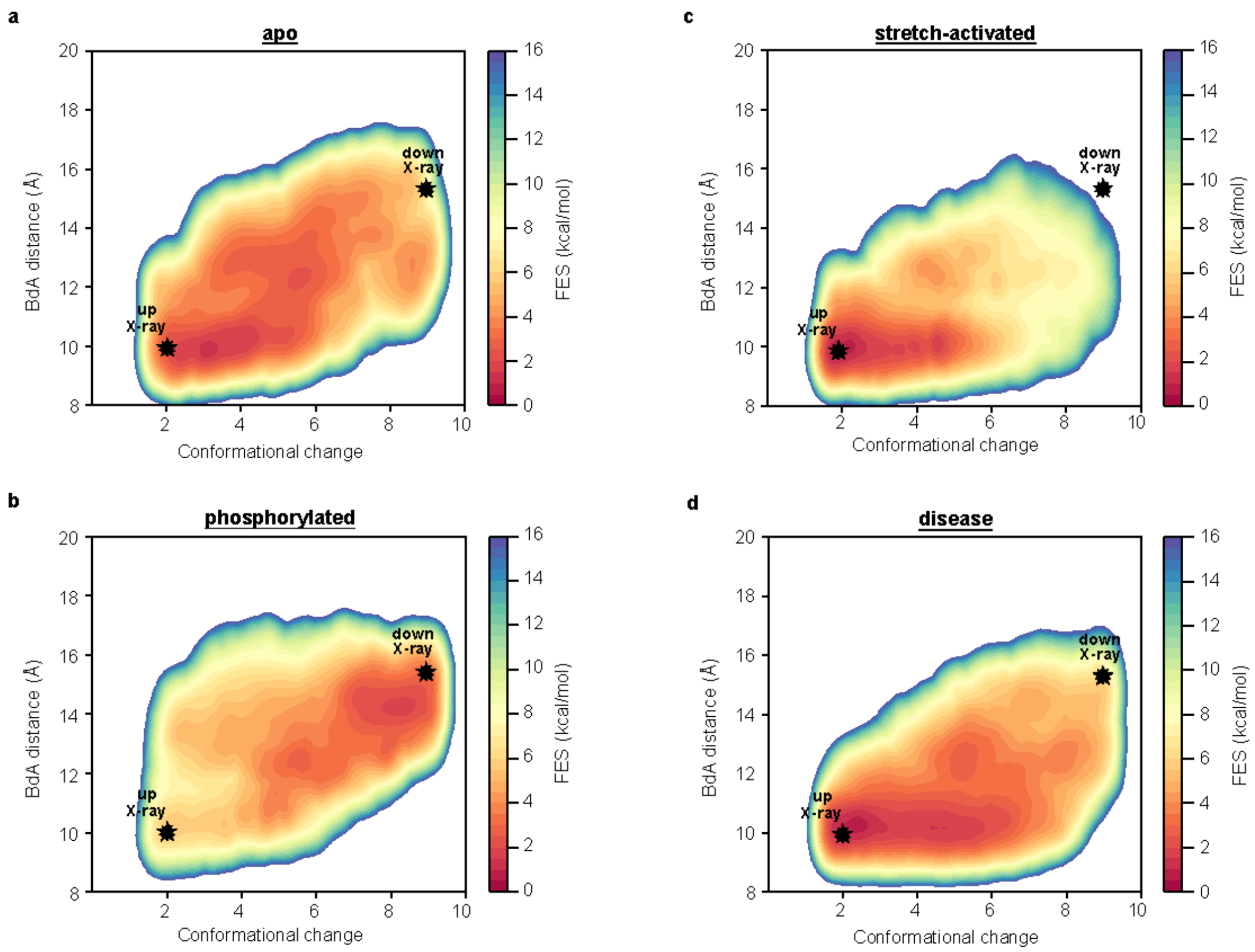

Supplementary Fig. 4. Analyses of the M4-pCt movement in TREK-2 OneOPES simulations. (a) Two-dimensional free energy surfaces projected along the path CV and the auxiliary CV describing M4-M2 distance for apo, **(b)** phosphorylated, **(c)** membrane stretch and **(d)** disease simulations. The average of three independent replica for apo, phosphorylated and membrane stretch and five independent replicas for disease system were calculated for two-dimensional free energy surfaces. Black stars indicate the positions of the crystallographic TREK-2 up- and down-state structures mapped onto these coordinates, based on their measured path CV values and BdA distances.

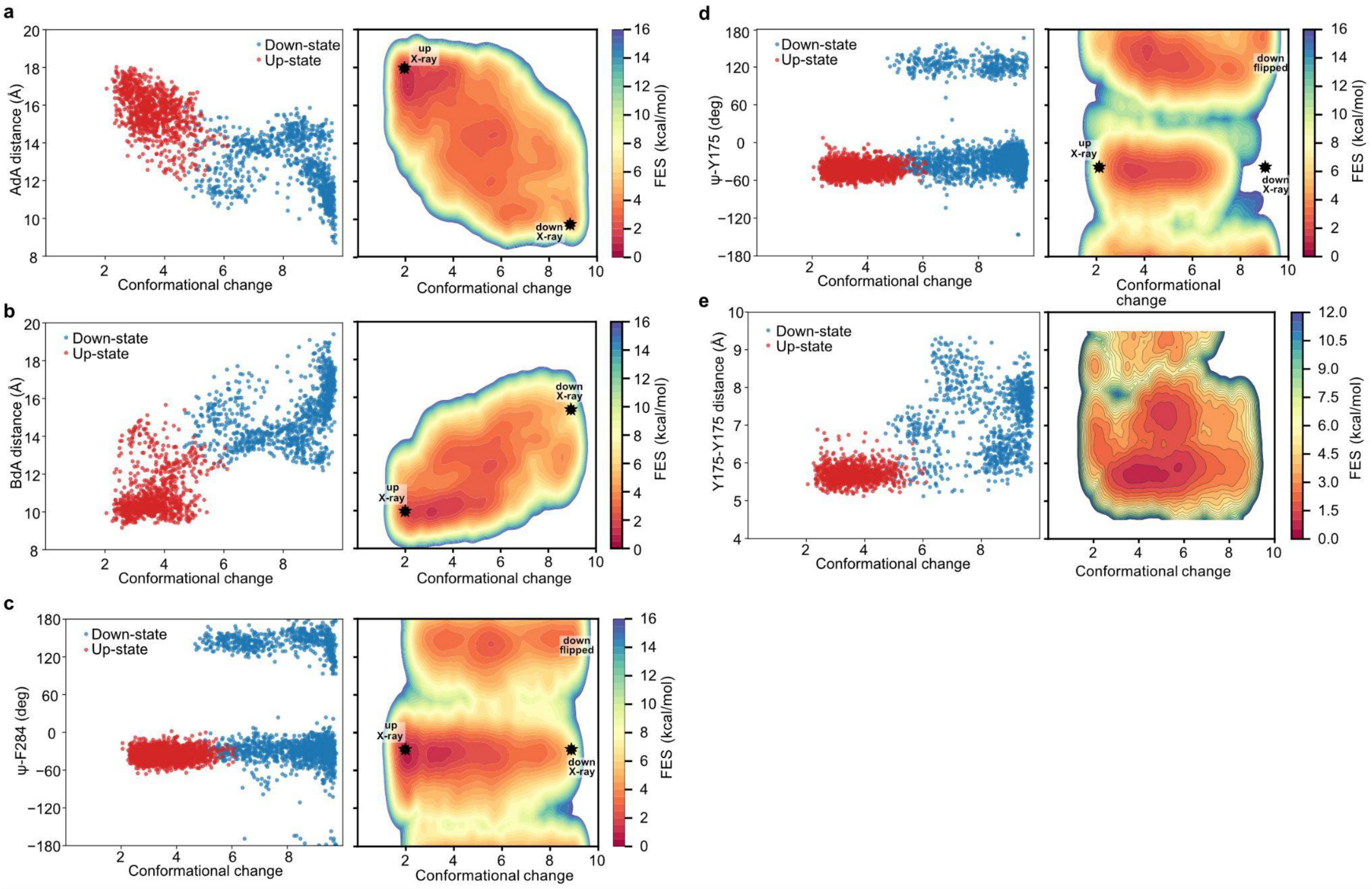

Supplementary Fig. 5. Projection of unbiased up- and down-state TREK-2* simulations onto the OneOPES collective-variable space, shown alongside the corresponding apo OneOPES free-energy surfaces. In each panel, the left plot shows conformations sampled in our previous unbiased MD simulations[1], five independent 1 µs replicas started from the up-state (red) and five from the down-state (blue), projected onto the collective variables used in the OneOPES setup and plotted against the path CV (s, "conformational change" on the x-axis), which describes the progression from the up-state (low s) to the down-state (high s). The right plot shows the two-dimensional free-energy surface (FES) reconstructed by reweighting the apo TREK-2* OneOPES ensemble (average of three independent replicas) projected along the path CV and the same variable, allowing a direct comparison between the regions spontaneously sampled in the unbiased ensemble and the energetic minima recovered by enhanced sampling. **(a)** Intra-chain M2–M4 distance (AdA, subunit A M2 to subunit A M4). **(b)** Inter-chain M4–M2 distance (BdA, subunit B M4 to subunit A M2). **(c)** Backbone ψ dihedral angle of F284 and **(d)** of Y175, both reporting on the SF conformation at the S1 K^+ binding site. **(e)** Inter-subunit Y175–Y175 backbone carbonyl distance, computed between the centres of mass of the backbone carbonyl atoms (C and O) of Y175 in the two opposing subunits. Black stars indicate the positions of the crystallographic TREK-2 up- and down-state structures mapped onto these coordinates; the "down flipped" label marks the flipped-carbonyl SF conformation accessible in the down-state region. In every panel, the unbiased up- and down-state ensembles partition into distinct regions of the path CV that coincide with the up- and down-state minima of the apo OneOPES FES. Free energies are given in kcal/mol.

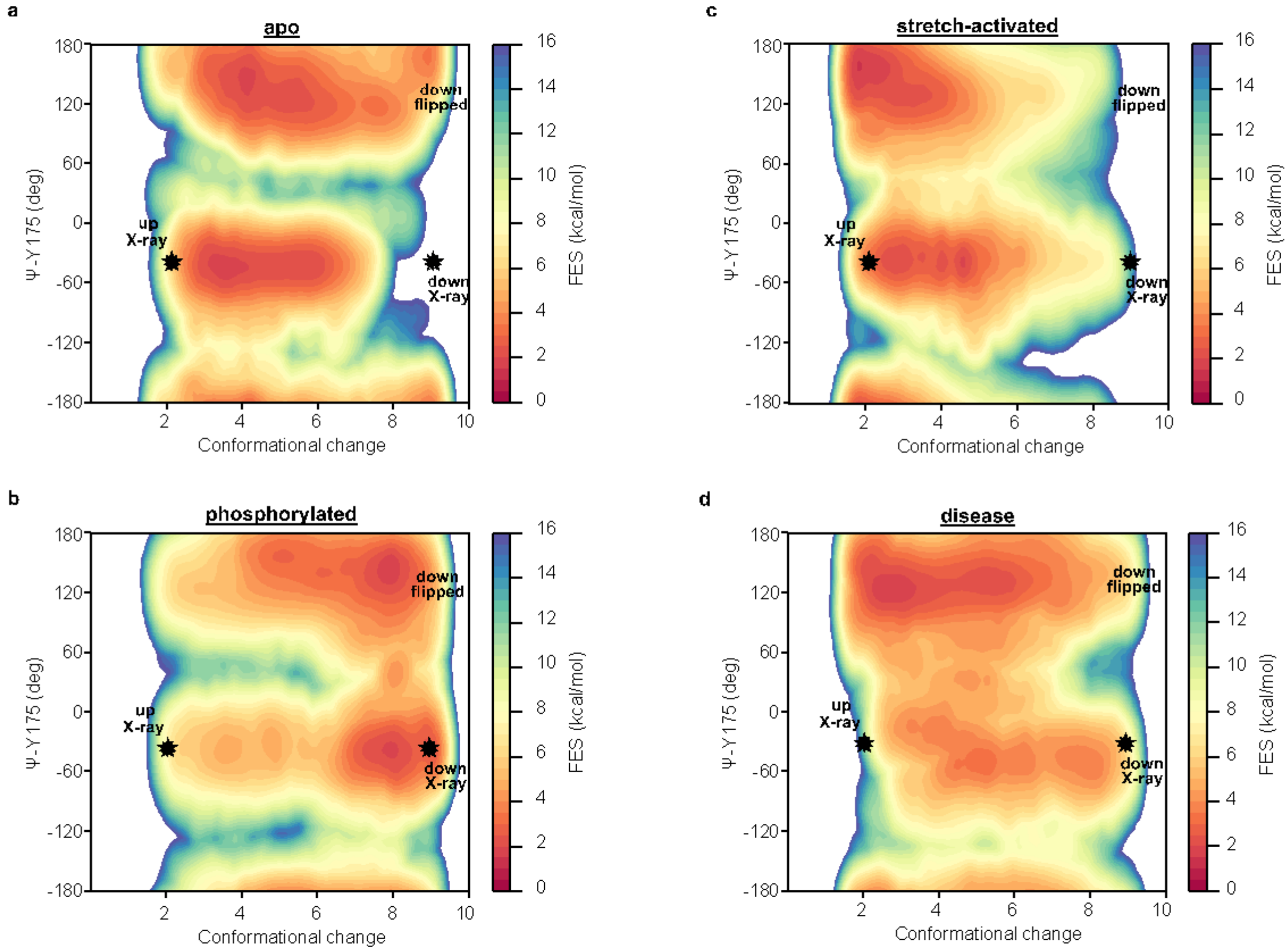

Supplementary Fig. 6. Analyses of the backbone conformation of Y175 in TREK-2 OneOPES simulations. (a) Two-dimensional free energy surfaces projected along the path CV and the auxiliary CV describing carbonyl rearrangement of Y175 SF residue for apo, **(b)** phosphorylated, **(c)** membrane stretch and **(d)** A314P mutant simulations. The average of three independent replica for apo, phosphorylated and membrane stretch and five independent replicas for A314P system were calculated for two-dimensional free energy surfaces.

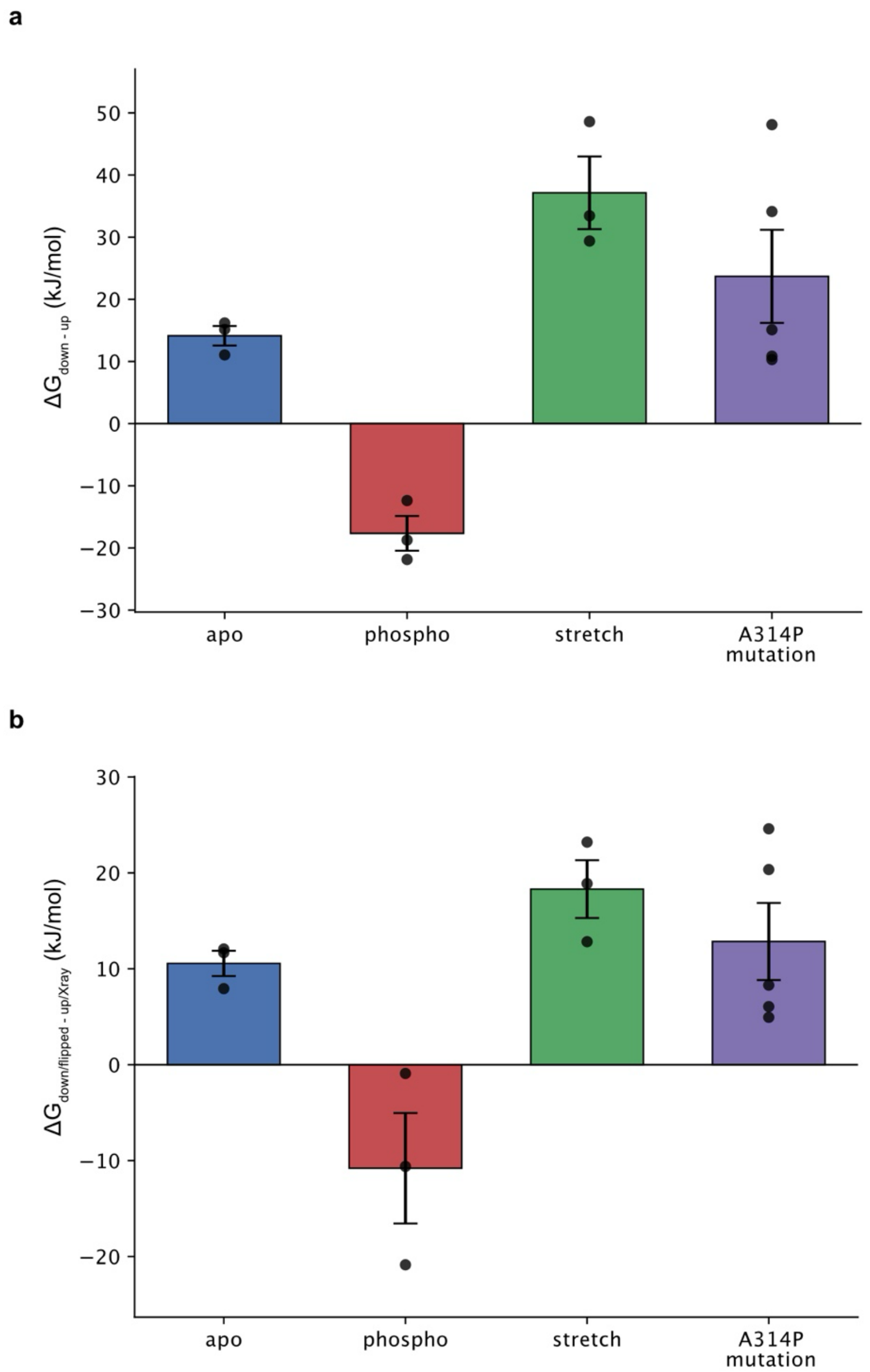

Supplementary Fig. 7. Quantitative comparison of up- and down-state free energies across conditions. Free-energy difference between the down- and up-state basins, for the apo, phosphorylated, stretch-activated and A314P (disease-mimicking) systems, obtained by Boltzmann integration of the reweighted populations within fixed basins defined on the two-dimensional free-energy surfaces. Positive values indicate that the up-state is thermodynamically favored; negative values indicate that the down-state is favored. Full basin definitions and the integration procedure are given in Methods (Basin-integrated free-energy differences). **(a)** ΔΔG calculations were performed by selecting basins from the path CV / M4–M2 (AdA) 2D FES, describing the global up–down conformational change of the M4/pCt module: the up-state basin combines low path-CV values with high AdA, and the down-state basin combines high path CV values with low AdA. **(b)** ΔΔG calculations were performed by selecting basins from the path CV / ψ-F284 2D FES: the up-state basin combines low path-CV values with the X-ray-like ψ-F284 conformation (−60° to −10°), and the down-state basin combines high path-CV values with the flipped ψ-F284 conformation (120° to 180°). Bars are the mean over independent OneOPES runs (n = 3 for apo, phosphorylated and stretch; n = 5 for A314P), error bars are the standard error of the mean, and black circles show the individual per-run values. Identical basin definitions were applied to all systems.

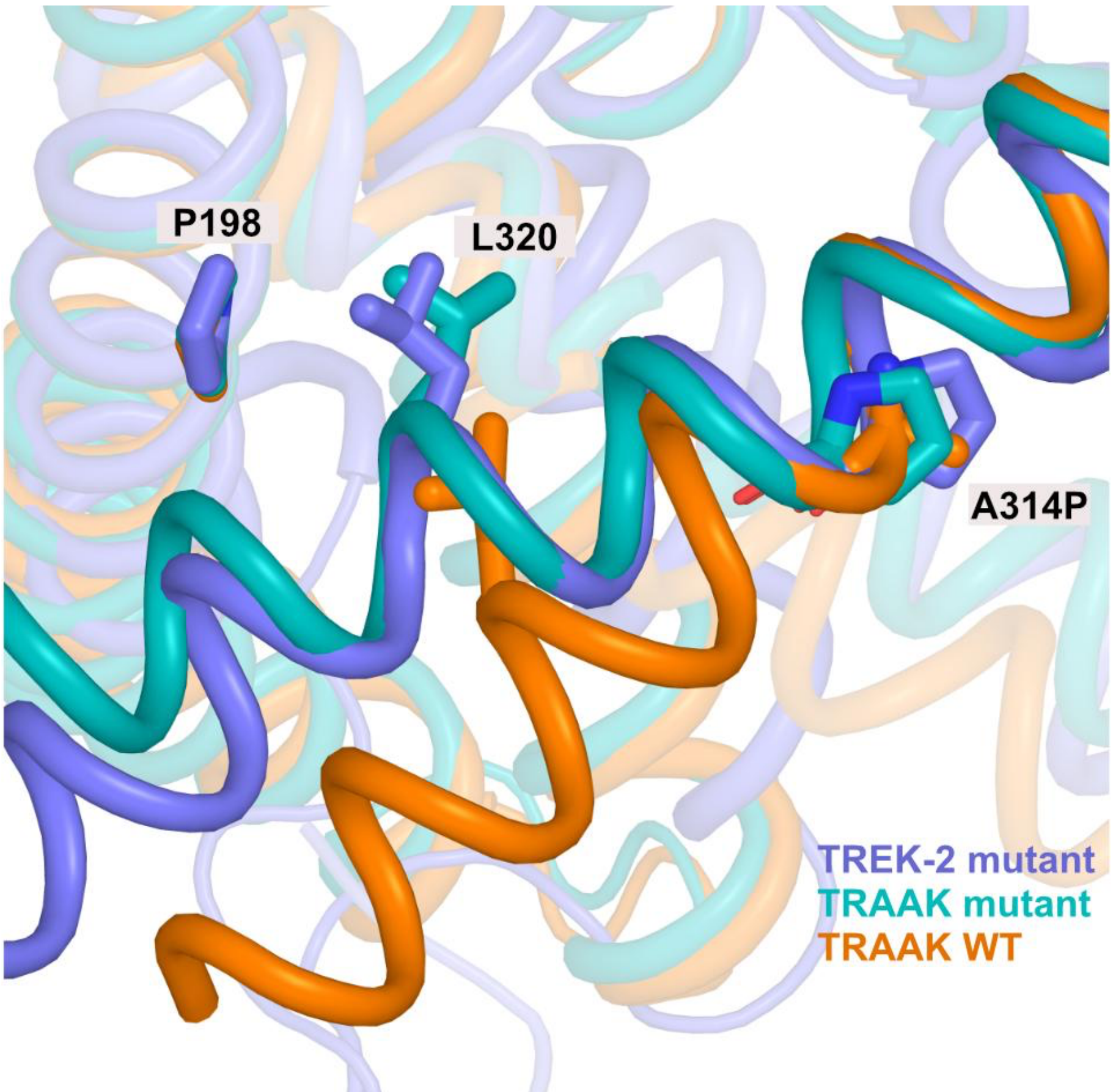

Supplementary Fig. 8. Structural comparison of the A314P-induced M4 kink in TREK-2. Overlay of a representative structure from the A314P TREK-2* up-state free-energy minimum (purple) with the experimentally determined TRAAK A270P structure (teal; PDB: 7LJ4[2]) and the down-state TRAAK structure (orange; PDB: 4WFF[3]). Structures were superimposed on the conserved pore domain so that the position of M4 relative to the rest of the channel is directly comparable; the view is centered on the M4 helix and the intracellular pCt–M4 interface. The substituted residue (A314P in TREK-2*; A270P in TRAAK), the M4 leucine (L320 in TREK-2*; L276 in TRAAK) and the M2 proline of the opposing subunit (P198 in TREK-2*; P155 in TRAAK) are shown as sticks. In both the TREK-2* A314P model and the TRAAK A270P structure, M4 adopts the kinked, upward-displaced up-state conformation and the two structures overlay closely, whereas in the TRAAK WT structure M4 is in the downward (down-state) position. The L320–P198 (L276–P155) side chains are in close contact in the two mutant structures, indicating that the kinked M4 conformation positions the pCt–M4 interface adjacent to the opposing M2 helix.

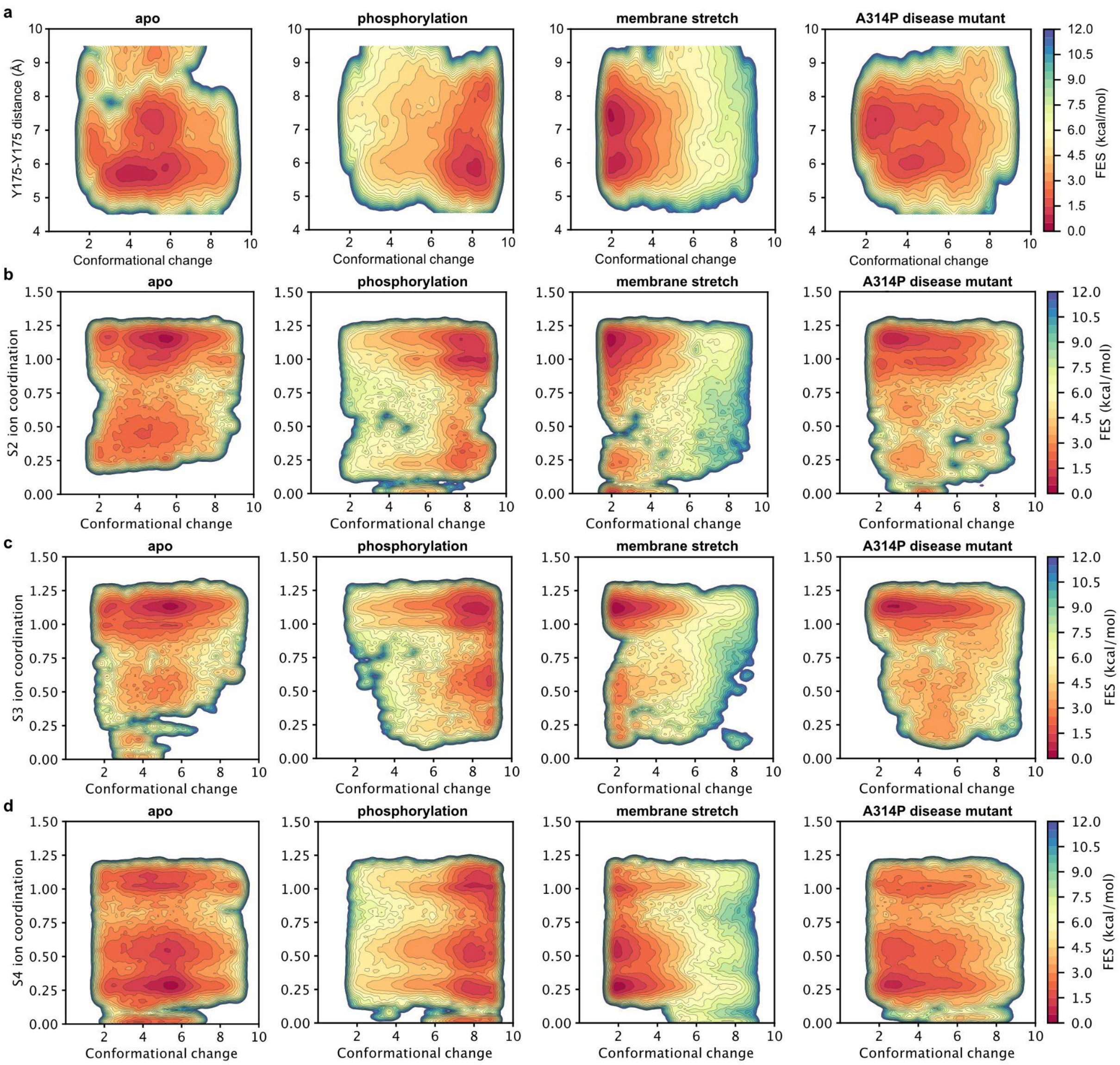

Supplementary Fig. 9. Multi-parameter characterization of the selectivity filter from the OneOPES simulations. Two-dimensional free-energy surfaces projected along the path CV ("conformational change"; up-state ≈ 2, down-state ≈ 8) and four additional selectivity-filter descriptors, reweighted from the OneOPES ensembles. **(a)** Inter-subunit Y175–Y175 Cα–Cα distance (Å), reporting on lateral breathing of the filter. **(b–d)** K^+ coordination number at the (**b**) S2, (**c**) S3 and (**d**) S4 binding sites, each defined as the coordination number between K^+ and the center of mass of the Cα atoms of the four residues lining the corresponding site. In each row, columns correspond, from left to right, to the apo, phosphorylated, membrane-stretch and A314P systems; each surface is the average of three independent OneOPES runs (five for A314P). The colour scale denotes the free energy in kcal mol^{-1}.

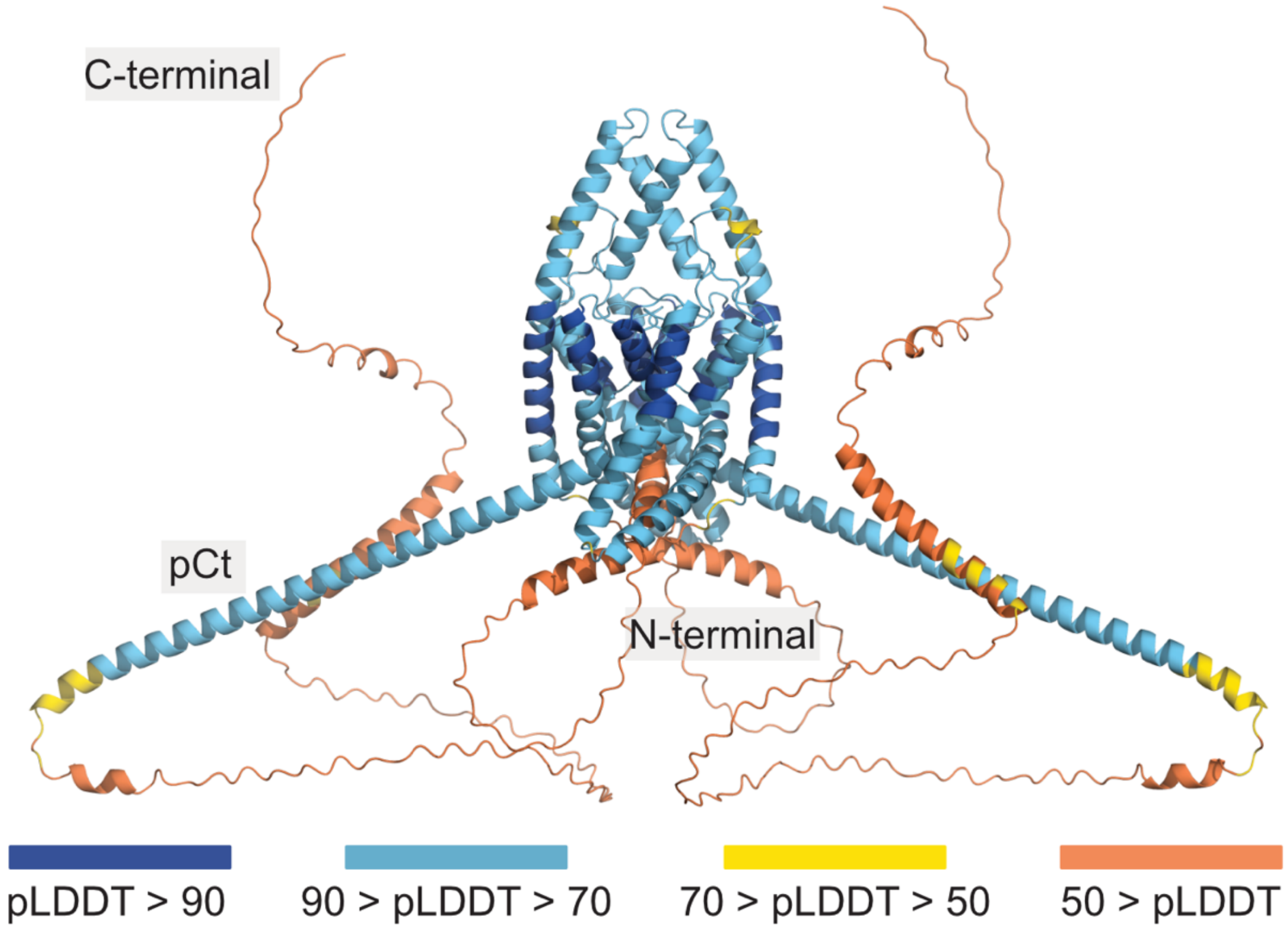

Supplementary Fig. 10. Predicted full-length structure of human TREK-2 channel using AlphaFold3[4].

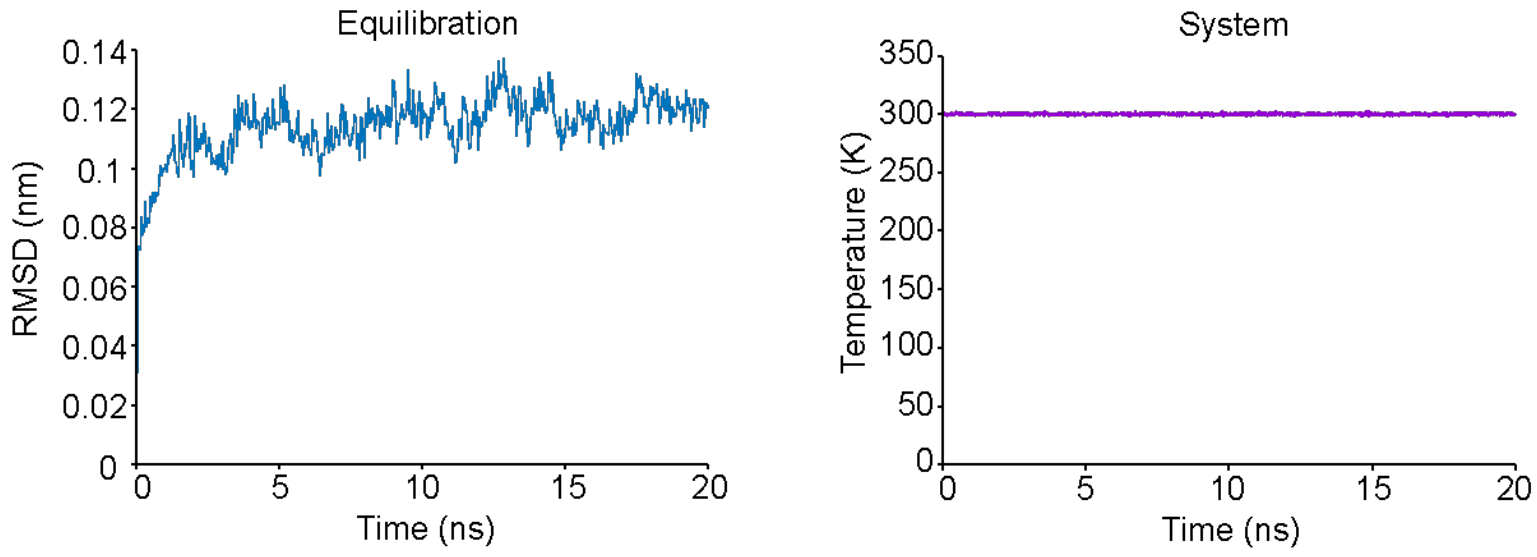

Supplementary Fig. 11. RMSD analysis of the MD data. The time course of root-mean-square deviation (RMSD) during the 20 ns equilibration and the temperature of the system during equilibration for structure of apo TREK-2.

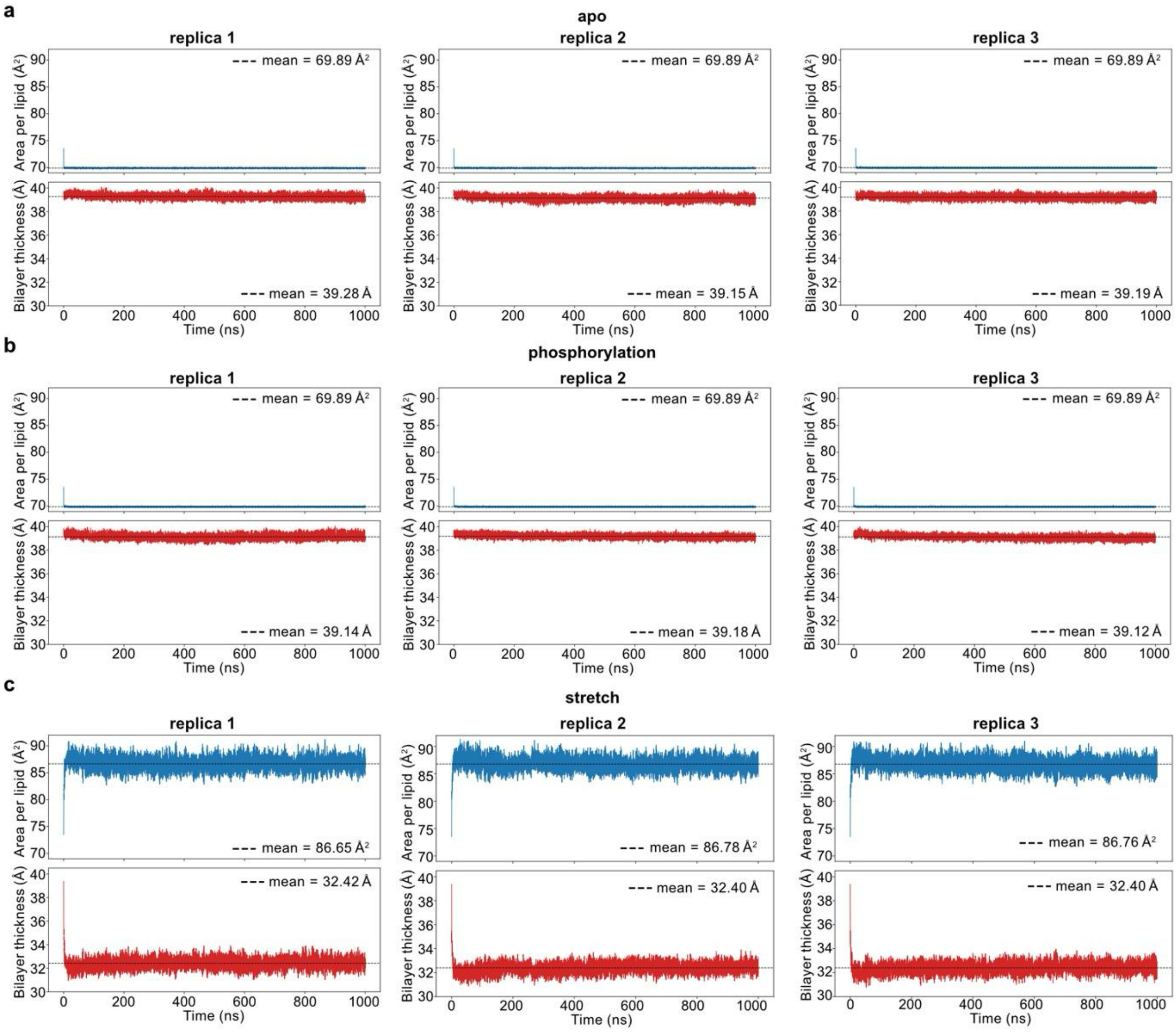

S
upplementary Fig. 12. Bilayer integrity assessment for the apo, phosphorylated, and membrane-stretched TREK-2* OneOPES simulations. Area per lipid (APL, upper) and phosphate–phosphate (P–P) bilayer thickness (lower) computed for the **(a)** apo**, (b)** phosphorylated, and **(c)** membrane-stretched systems. For each system, three independent replicas are shown. APL was calculated by dividing the instantaneous bilayer (xy) area by the number of POPC molecules per leaflet, and P–P thickness was computed as the center-of-mass distance along the membrane normal between the phosphorus atoms of the two leaflets, both sampled every 100 ps. The apo and phosphorylated systems were simulated under standard semi-isotropic pressure coupling (+1 bar in the xy-plane, +1 bar along z); the membrane-stretched system was simulated with a lateral pressure of −50 bar in the xy-plane while the normal pressure was maintained at +1 bar. Under applied tension, the APL increases and the P–P thickness decreases relative to the unstretched apo and phosphorylated references, in agreement with the expected response of POPC bilayers to lateral stretch, and both observables stabilize around new mean values that remain constant across the remainder of the trajectories. No membrane rupture or pore formation was observed in any replica.

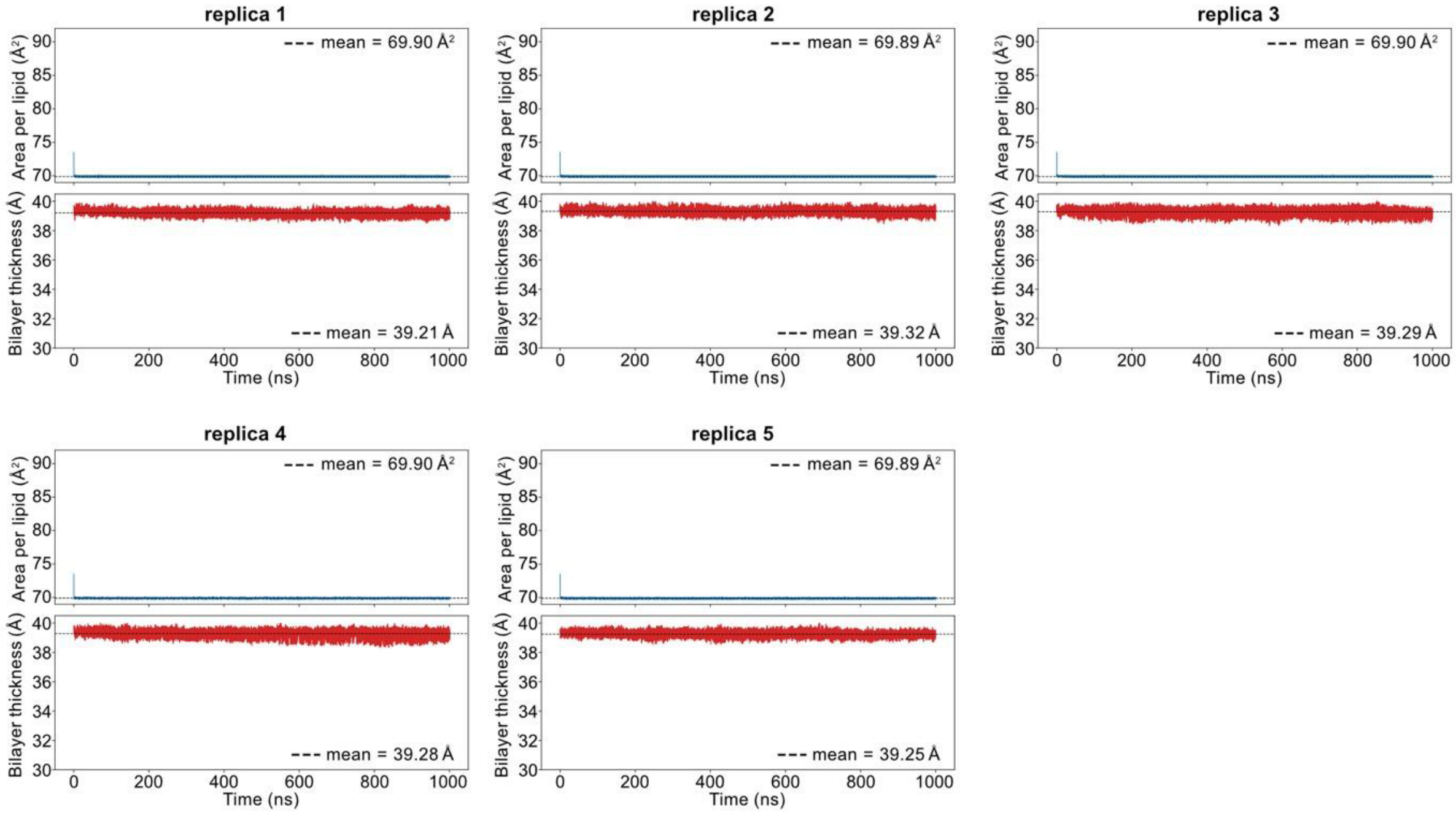

Supplementary Fig. 13. Bilayer integrity assessment for the A314P disease-mimicking TREK-2* OneOPES simulations. Area per lipid (APL, upper) and phosphate–phosphate (P–P) bilayer thickness (lower) computed for the five independent replicas of the A314P TREK-2* system. APL and P–P thickness were calculated as described in Supplementary Figure 12, with semi-isotropic pressure coupling at +1 bar in both the xy-plane and along z. Both observables remain stable around their respective means throughout the production phase across all five replicas, and the values are comparable to those obtained for the apo system (Supplementary Figure 12a), supporting that the bilayer is structurally intact under the conditions used to evaluate the disease-mimicking mutation.

Supplementary Table 1. Correspondence between TRAAK and TREK-2 residue numbering systems for the FHEIG mutations

Mutation	TRAAK UniProt (Q9NYG8)	TRAAK PDB 4WFE	TREK-2 PDB 4BW5	Source	Used in this study
FHEIG mutation 1	A244P	A270P	A314P	Bauer et al. 2018	Yes (modelled in TREK-2)
FHEIG mutation 2	A172E	A198E	T241	Bauer et al. 2018	No

Supplementary Table 2. CVs and parameters used in the TREK-2 OneOPES simulations. The rows labeled "OPES Explore" and "OPES MultiCV" indicate how the CVs were distributed across the different replicas. The "OPES MultiT" row reports the maximum temperature reached during the simulations, relative to the thermostat temperature (300 K).

Replicas	0	1	2	3	4	5	6	7
OPES Explore	p1.s	p1.s	p1.s	p1.s	p1.s	p1.s	p1.s	p1.s
OPES $MultiCV_1$	–	m2bm4a	m2bm4a	m2bm4a	m2bm4a	m2bm4a	m2bm4a	m2bm4a
OPES $MultiCV_2$	–	–	m4am2a	m4am2a	m4am2a	m4am2a	m4am2a	m4am2a
OPES $MultiCV_3$	–	–	–	psiTa	psiTa	psiTa	psiTa	psiTa
OPES $MultiCV_4$	–	–	–	–	psiFb	psiFb	psiFb	psiFb
OPES $MultiCV_5$	–	–	–	–	–	V1, V2	V1, V2	V1, V2
OPES $MultiCV_6$	–	–	–	–	–	–	–	–
OPES $MultiCV_7$	–	–	–	–	–	–	–	–
OPES MultiT	–	301K	303K	306K	310K	317K	325K	335K

Supplementary Table 3. Pairwise free-energy comparisons (ΔΔG) between apo and the other conditions. Differences in the basin-integrated free-energy difference ΔG(down–up) between apo and each other condition, evaluated on the two-dimensional reweighted free-energy surfaces along the M4/pCt (AdA) and selectivity-filter (ψ-F284) coordinates. ΔΔG is defined as ΔG(apo) − ΔG(other); a positive value indicates that the up-state is more strongly favored in apo than in the comparison condition, and a negative value the opposite. Uncertainties are propagated from the per-condition standard errors as $\sigma(\Delta\Delta G) = \sqrt{[SE(apo)^2 + SE(other)^2]}$, and the separation is reported as $|\Delta\Delta G|/\sigma$. Per-condition ΔG(down–up) values are means over independent OneOPES runs (n = 3 for apo, phosphorylated and stretch-activated; n = 5 for A314P). The same ordering of conditions is recovered on both coordinates. All values in kJ mol^{-1}.

comparison	ΔΔG	SE	σ
**M4/pCt (AdA) projection**			
apo − phospho	31.80	3.20	9.9
apo − stretch	−23.00	6.05	3.8
apo − disease	−9.56	7.65	1.2
**SF (ψ-F284) projection**			
apo − phospho	21.35	5.91	3.6
apo − stretch	−7.75	3.29	2.4
apo − disease	−2.29	4.23	0.5

Supplementary Table 4. M4 kink angle and inter-subunit M4–M2 contact distance for the A314P TREK-2* and reference WT TRAAK structures. Values are reported for a representative structure selected from the A314P TREK-2* up-state free-energy minimum, the experimentally determined TRAAK A270P structure (PDB: 7LJ4), and the down-state TRAAK structure (PDB: 4WFF). The M4 kink angle quantifies the local bend of M4 at the substituted position. The M4-M2 distance corresponds to the minimum side-chain heavy-atom distance between the M4 leucine (L320 in TREK-2*; L276 in TRAAK) and the M2 proline of the opposing subunit (P198 in TREK-2*; P155 in TRAAK). Both proline-mutated structures (TREK-2* A314P, TRAAK A270P) show a larger M4 kink and a shorter inter-subunit M4-M2 contact than the TRAAK WT down-state.

Structure	Kink angle chain A (°)	Kink angle chain B (°)	M4–M2 distance pair 1 (Å)	M4–M2 distance pair 2 (Å)
A314P (model)	67.3	65.4	4.1	3.7
TRAAK A270P (PDB 7LJ4)	62.9	63.7	4.2	3.6
TRAAK WT (PDB 4WFF)	58.7	55.7	7.1	4.6

Supplementary Table 5. Simulation details of each simulation setup

System	*Total N of atoms*	*Total N of H_2O*	*Salt concentration*	Total N of lipid composition (POPC 52 atoms)	*Box dimensions (Å)*
TREK-2 apo OneOPES	121627	32253	612 mM	295	104.1x104.1x140.9
TREK-2 phosphorylated OneOPES	121629	32253	612 mM	295	104.1x104.1x140.9
TREK-2 stretch OneOPES	121627	32253	612 mM	295	104.1x104.1x140.9
TREK-2 disease mutant OneOPES	121635	32253	612 mM	295	104.1x104.1x140.9

References

1. Türkaydin, B. *et al.* Atomistic mechanism of coupling between cytosolic sensor domain and selectivity filter in TREK K2P channels. *Nat Commun* **15**, 4628 (2024).
2. Rietmeijer, R. A., Sorum, B., Li, B. & Brohawn, S. G. Physical basis for distinct basal and mechanically gated activity of the human K+ channel TRAAK. *Neuron* **109**, 2902-2913.e4 (2021).
3. Brohawn, S. G., Campbell, E. B. & MacKinnon, R. Physical mechanism for gating and mechanosensitivity of the human TRAAK K+ channel. *Nature* **516**, 126–130 (2014).
4. Abramson, J. *et al.* Accurate structure prediction of biomolecular interactions with AlphaFold 3. *Nature* **630**, 493–500 (2024).
