# Supplementary material for: Cross-talk of filter gate and lower helices drives polymodal regulation and disease in TREK channels
Source: Nat Commun. 2026 Sep 22;17:11029. doi: 10.1038/s41467-026-77935-w (PMC13687069; doi:10.1038/s41467-026-77935-w)
Supplement: Supplementary file 3 [file 41467_2026_77935_MOESM3_ESM.pdf]

nature portfolio

Peer Review File

Cross-talk of filter gate and lower helices drives polymodal regulation and disease in TREK channels

Corresponding Author: Professor Han Sun

This file contains all reviewer reports in order by version, followed by all author rebuttals in order by version.

Version 0:

Reviewer comments:

Reviewer #1

(Remarks to the Author)
Energetic cross-talk of filter gate and lower helices drives polymodal regulation and disease in TREK K2P channels

The manuscript addresses an important biological question — how phosphorylation, mechanical stretch, and a disease-associated mutation reshape the conformational landscape of TREK-2 — and the OneOPES enhanced sampling framework is in principle well-suited to this problem. The electrophysiological validation of the A314P mutant using NFx inhibition is a strength. However, after careful reading of the main text and supplementary material, I have identified a series of problems that range from factual inaccuracies and missing controls, to a systematic pattern of overclaiming that I will detail below. In my view, the manuscript requires major revision, with additional simulations and analyses, before it can be considered for publication. I will organize my remarks by category.

I. Factual errors and presentation problems
At line 84, the text refers to "Fig. 1b," which does not exist. Fig. 1 contains only panel (a). Either the reference is wrong, or a panel was removed during revision without updating the text. This must be corrected.
At line 360, the text discusses SF stability for the A314P mutant and cites Fig. 5e, which shows M4-M2 distances. The relevant panel for SF stability is Fig. 5f. This is an error.
At line 487, "pays the way" should read "paves the way."
Lines 57-58 describe two FHEIG mutations in TRAAK, A244P and A172E. Later, lines 299-300 introduce A270P in TRAAK and its TREK-2 homologue A314P as the basis for the simulations. These are different mutations from different patients. The transition between them is never explicitly explained, and a reader who is not a specialist in this disease will be confused. Please clarify which mutation is being studied computationally and why, and verify the genomic and structural assignments at lines 57-58 against the primary sources.

II. Modelling choices that are either unjustified or misleading
The pCt was modelled computationally using PyMOL builder with a forced helical conformation (line 497). The legend of Fig. 1 labels these structures "X-ray extended," which strongly implies experimental determination. This is incorrect and misleading. The label should read something like "computationally modelled pCt" and the methods section should provide the structural or biochemical justification for imposing an α-helical conformation. Citing the authors' prior work is not sufficient — the original justification for this choice needs to appear somewhere.
The membrane is composed exclusively of POPC (line 504, 295 lipids). Given that the fenestration-based lipid-block mechanism is central to the discussion of TREK gating, and given the known effects of phosphoinositides and other anionic lipids on K2P channels, a POPC-only bilayer is a significant simplification. The authors cannot address lipid specificity with this setup, and should at minimum acknowledge this as a limitation.
The KCl concentration of 600 mM (line 505) is justified by reference to prior unbiased simulations where accelerating ion conduction was the goal. However, in the present study the sampling is already accelerated by OneOPES. The original justification no longer applies with the same force. Given that the pCt carries multiple charges and the phosphoserine is modelled at -2, a concentration three to four times above physiological is not trivially innocuous, while commonly used in MDs. The authors should discuss whether this choice affects the relative stability of up versus down states.
The equilibration validation is limited to RMSD and temperature (Supp. Fig. 4). No membrane area per lipid, bilayer thickness, or lateral pressure profile is reported. These are standard validators for membrane systems and their absence makes it impossible to judge whether the initial membrane density was appropriate, or whether the choice to restrict cell

fluctuations to the z-direction (line 516) was justified for this particular system.

III. The path CV: a coordinate is not a pathway, but its limitations must be addressed
The authors construct the path collective variable using 10 structures generated by PyMOL morphing between the up and down crystal structures, equally spaced in RMSD (lines 553-555). I want to be precise here, because this point is often misunderstood. The morph structures are reference points that define a progress coordinate s — they do not constrain the trajectory to follow this interpolated path. I understand this. Nevertheless, there are two problems that are not addressed. First, the Branduardi path CV also defines a distance-from-path variable z, which measures how far the instantaneous conformation is from the interpolated tube. This quantity is never shown, neither in the main figures nor in the supplement. Without it, the reader cannot know whether the system remains close to the morphed path or whether it explores conformations that are geometrically distant from all 10 reference models. If z is large, the s values reported in the FES are ambiguous projections. They are rather ambiguous as progress coordinates. The authors must show the z distribution, or provide an explicit argument for why this is not necessary.
Second, and more fundamentally: the path CV biases exploration along the predefined up↔down axis. Any conformation that lies off this axis — a third basin, a metastable intermediate not present in either crystal structure, a conformation accessible under physiological conditions but geometrically distant from the morph interpolation — will not be efficiently promoted, because the bias along s provides little driving force for off-path exploration. The multi-thermal replicas partially compensate for this, but not in a targeted way. This is a statement about what any path-based CV can and cannot do. The authors never acknowledge this limitation, and they should.
The practical consequence is the following. Several conclusions in the manuscript — that the system occupies two and only two relevant states, that the FES minima are genuine thermodynamic basins rather than artifacts of the coordinate definition, that the SF conformation observed in those minima is stable — cannot be fully verified from the biased simulation alone. The most direct and least expensive way to address this is to perform short unbiased MD simulations, of the order of 300-500 ns, initiated from the FES minimum structures for each condition. Here the target are stability/verification runs, three replicates allowing some basic variance assessment. If those structures are genuinely stable, the SF conformation, the M4 position, and the key CV values should be maintained without bias. If they are not, the FES minima do not correspond to physically meaningful states.

IV. Circular reasoning in the validation of auxiliary CVs
The correlation between the path CV and the M4-M2 auxiliary CVs (AdA, BdA) is presented as validation that the auxiliary CVs "faithfully track the structural rearrangements that accompany the global gating motion" (lines 193-195). This is not independent validation. The path CV was constructed from the RMSD of Cα atoms between the up and down crystal structures, and M4 undergoes the largest displacement between those two structures. Any coordinate that measures M4 position will correlate with s by geometric necessity. The paragraph at lines 190-208 does not go beyond what was already implied by the path construction. The authors should either provide genuinely independent validation — for example, using a structural observable that was not part of the path definition — or remove the claim of validation and rephrase this section as a description of what the auxiliary CVs capture.
The same logic applies to lines 231-233, where the correlation between path CV and ψ-F284 is described. The path CV encodes the global structural difference between up and down, which includes the SF environment. A correlation between s and ψ-F284 does not establish a mechanical link between M4 position and SF dihedral.

V. The allosteric coupling claim is not supported by the data
This is the most serious problem in the manuscript, and it recurs at lines 247-249, 262-263, 288-293, 451-453, 471-473, and 484-485. In each case, the authors observe that under a given condition (phosphorylation, stretch, mutation), both the M4-pCt region and the SF adopt a particular configuration in the FES, and from this they conclude that M4-pCt motion "allosterically controls" or "directly controls" the SF gate.
This reasoning is not sufficient to establish a casual link. The up and down crystal structures differ globally — not only in M4 position, but also in the environment of the SF, in inter-helix contacts, in pore geometry, and in many other coordinates. When the system is in the down-state region of the FES, the SF is in a particular conformation not because M4 has acted on it allosterically, but because the entire protein is in a different global state. To claim allostery, one needs to isolate the M4-SF coupling from the global structural change. This could be done, for example, by showing that constraining M4 in the up-position while the rest of the protein is in a down-like conformation still stabilizes the canonical SF — or by performing unbiased simulations starting from a down-state structure under stretch conditions and observing whether M4 moves before or after the SF changes. Neither analysis is present.
I would ask the authors to either remove the word "allosteric" throughout, replacing it with more conservative language about co-variation or thermodynamic association, or to provide the additional data needed to support a causal claim. The latter would substantially strengthen the paper.

VI. The selectivity filter characterization is insufficient
The SF state is characterized in this study almost entirely through the ψ-F284 dihedral angle. This is a very limited proxy. In the K2P literature, SF state is typically assessed using a combination of parameters: ion occupancy at all binding sites (S1-S4), inter-subunit distances between conserved tyrosines (Y175-Y175 in this case), carbonyl orientations at multiple filter positions, pore hydration and dewetting, and backbone flexibility. Of these, only K+ coordination at S1 (CV6) and ψ-Y175 are additionally reported, and the Y175 results are acknowledged to be unreliable (lines 252-259). The S2-S4 ion occupancies are not reported. The Y175-Y175 inter-subunit distance, a classical indicator of SF collapse in K2P channels, is absent. Pore hydration is absent.
Figure 6 presents a clear and appealing narrative in which up = conductive SF and down = non-conductive SF. But this narrative rests almost entirely on ψ-F284, and even there it is complicated: in Fig. 4b (apo), a metastable flipped-SF conformation is clearly visible within the up-state region of the FES, which the authors acknowledge at lines 236-237 but

then contradict at lines 417-418 where they write that "the SF remains in a stable, conductive configuration" in the up-state. These two statements cannot both be true. The authors should reconcile them and provide a more complete characterization of the SF in each FES minimum, using the additional parameters listed above.
The SF state is characterized in this study almost entirely through the ψ-F284 dihedral angle, with partial and acknowledged-unreliable contributions from ψ-Y175. This is a very limited proxy for a structure as functionally complex as the K2P selectivity filter. In the literature, SF state is typically assessed using a combination of parameters: ion occupancy at all binding sites (S1-S4), inter-subunit distances between conserved tyrosines — here Y175-Y175 — which report on SF lateral breathing and collapse, carbonyl orientations at multiple filter positions, pore hydration and dewetting transitions, and backbone flexibility. Of these, only K+ coordination at S1 (CV6) is additionally reported, and the Y175 dihedral results are themselves acknowledged to be unreliable due to missing CVs (lines 252-259). S2-S4 ion occupancies are absent. The Y175-Y175 inter-subunit distance, a classical indicator of SF state in K2P channels, is not shown. Pore hydration is not discussed.
This matters because Figure 6 presents a clean and visually compelling narrative in which up-state equals conductive SF and down-state equals non-conductive SF, and this figure is the central summary of the paper. But it rests almost entirely on ψ-F284, and even there the picture is not as clean as Fig. 6 suggests. In Fig. 4b (apo), a metastable flipped-SF conformation is clearly visible within the up-state region of the FES, at approximately 3 kcal/mol above the minimum. The authors acknowledge this at lines 236-237, but then at lines 417-418 they write that "within these conformations, the SF remains in a stable, conductive configuration as revealed in the crystal structure." These two statements are in direct contradiction and need to be reconciled.
More broadly, the reader is asked to accept that ψ-F284 is a sufficient reporter of conductive versus non-conductive SF states, but no justification for this choice is provided beyond the authors' prior work. Given that the central claim of the paper is an allosteric coupling between M4-pCt dynamics and SF gating, the SF side of this coupling deserves a much more complete characterization. The authors should provide, for each FES minimum identified in Figs. 4 and 5, at minimum the ion occupancy profile across S1-S4, the Y175-Y175 inter-subunit distance distribution, and the pore hydration state, extracted from the reweighted ensemble.
Here again, short unbiased MD simulations initiated from the FES minima would be of direct value. If a structure extracted from the conductive FES minimum is placed in an unbiased simulation and the SF remains conductive — meaning ψ-F284 stays canonical, ions occupy the filter, and the pore remains hydrated — this would constitute genuine evidence that the FES minimum corresponds to a functional conductive state. The same test applied to the non-conductive minimum would complete the argument. Without this, the functional interpretation of the FES in terms of ion conduction remains an inference, not a demonstration. The lengths and replicate numbers are very important here, given the SF dynamics in K2P channels, so that 3 times 300-500 ns would be a minimum, without asking the authors to redo everything, which is not my idea.

VII. The absence of transmembrane voltage affects more than Y175
At lines 429-430, the authors invoke the absence of transmembrane voltage as an explanation for the discrepancy in Y175 sampling between the current study and their prior unbiased simulations. This is reasonable, but the framing is too narrow. Transmembrane voltage affects the global electrostatic environment of the channel — ion occupancy profiles, pore hydration, and potentially the M4-pCt equilibrium itself. By mentioning voltage only in the context of the Y175 discrepancy, the authors implicitly suggest that all other results are unaffected, which is not justified. The limitations section of the discussion should acknowledge voltage as a general limitation of the current setup.

VIII. Missing statistical analyses
The 2D FES plots in Supplementary Figs. 2 and 3 are presented purely visually, with no statistical assessment of whether the distributions under different conditions are significantly different from each other. The standard deviation shown in Fig. 2b-d gives some sense of reproducibility, but this is not a test of whether, for example, the apo and phosphorylated landscapes differ significantly. The paper could benefit from quantitative statistical comparisons. Quantitative comparison of the FES minima across conditions — for example, overlap metrics or confidence intervals on the free energy differences between states — could be provided before drawing conclusions about which condition favors which conformation.
Furthermore, at line 424, the word "correlation" is used to describe a visual observation from 2D FES plots. Correlation has a specific statistical meaning. If the authors wish to claim a correlation between M4-pCt displacement and SF stability, they should quantify it.

IX. Claims without supporting data
Several statements in the discussion are not supported by any figure or analysis in the paper.
Lines 338-343 describe a kink in M4 introduced by the A314P substitution, with a specific orientation that "forces" the pCt-M4 interface toward the opposing M2 helix. The kink is plausible given the general structural effect of proline substitutions, but its orientation and the specific claim about M2 proximity are inferred entirely from the FES minimum position, not from any direct structural analysis. No representative structures from the A314P trajectory are shown overlaid on WT. No contact distances, kink angles, or helix geometry measurements are provided. The authors should show the structural basis for this claim.
Lines 431-434 claim that incorporating Y175 as a CV "proved critical for improving the overall description of SF flexibility and for facilitating the proper sampling of F284 rearrangements." There is no figure or control simulation supporting this claim. No comparison with a simulation lacking Y175 as a CV is shown. This statement should be either supported with data or removed.
Lines 411-412 state that "TREK-2 operates through two states of the lower helices corresponding to crystallographic up and down conformations." This is presented as a finding, but it is built into the approach by construction: the path CV was defined between those two states, and the FES will by definition show minima near those endpoints. It would be a genuine

finding if the authors had started from an unbiased ensemble and observed spontaneous partitioning into these two states. The framing should be adjusted accordingly.

About the abstract

The abstract makes three claims that deserve scrutiny in light of the detailed problems identified in the full manuscript.

The phrase "detailed energetic characterization of the TREK-2 conformational landscape" overstates what is achieved. The conformational landscape explored is, by construction, the one-dimensional axis between two known crystal structures, defined by a path CV built from their RMSD difference. A landscape explored along a predefined coordinate between two known endpoints is not the same as a general characterization of the conformational space available to the channel. An unknown third basin, or a conformation not present in either crystal structure, would not be detected by this approach. The word "detailed" should be replaced by something more accurate, such as "energetic characterization of the up-to-down conformational transition."

The phrase "our simulations uncover a unifying mechanism in which coupling between the lower transmembrane helices, the proximal C-terminal domain, and selectivity filter stability governs channel gating" is the most problematic sentence in the abstract, and arguably in the entire manuscript. As detailed in sections V and VI of this review, what the simulations show is a thermodynamic co-variation between M4-pCt position and SF dihedral state within a biased FES. This is not a mechanism. A mechanism requires demonstration of a causal pathway — which residues communicate the signal, in which order, through which interactions. None of this is provided. Furthermore, the SF characterization rests almost entirely on a single dihedral angle (ψ-F284), which is an insufficient proxy for the functional state of a K2P selectivity filter. The word "mechanism" should be removed from the abstract and replaced with "thermodynamic association" or "energetic coupling," pending the additional analyses requested in the full review.

The final sentence, claiming that the results "provide a foundation for structure-based drug discovery," is unsupported by anything in the manuscript. No ligand binding, no druggable site identification, no pharmacophore analysis is presented. The NFx experiment validates a conformational shift but is not a drug discovery study. This sentence should be removed or substantially toned down.

The one sentence that is well-supported is the description of the electrophysiological validation using NFx — this is the strongest part of the paper and accurately described in the abstract.

Summary

The biological question addressed in this manuscript is important, and the electrophysiological validation of the A314P mutant using norfluoxetine is genuinely informative and constitutes the strongest contribution of the work.

However, I have concerns about the computational part of the study that I believe need to be addressed before the manuscript can be considered for publication. The claims made in the abstract — a mechanistic characterization of allosteric coupling, a detailed exploration of the conformational landscape — are not fully supported by the data presented. What is actually delivered is an energetic description of the transition between two known crystal structures along a predefined coordinate, with thermodynamic associations between M4-pCt position and SF dihedral state that are, at least in part, geometrically implied by the way the path collective variable was constructed. This is a useful contribution and worth publishing, but it is not the same as a mechanism, and it is not a landscape exploration in any general sense.

The allosteric coupling between M4-pCt dynamics and SF gating — presented as the central mechanistic finding — is inferred from co-variation of observables within a biased simulation, without causal analysis. The SF characterization rests almost entirely on a single dihedral angle, which is an insufficient proxy for the functional state of a K2P filter. Several statements in the discussion refer to structural features or mechanistic pathways for which no supporting figure or analysis is provided. A small number of factual errors and figure cross-reference mistakes also need to be corrected.

The path to publication is clear, but it requires additional analyses and a recalibration of the language throughout, starting from the abstract, to bring the claims into closer correspondence with what the data show. I detail what is needed in the sections above and recommend major revision accordingly.

Reviewer #2

(Remarks to the Author)

The manuscript by Turkaydin et al. used MD simulation to understand the energetic landscape of the effects of S331 phosphorylation and a disease-causing gain-of-function mutation on the conformational landscape of TREK2. The work also highlights key experimental evidence supporting the findings. Although the findings on the effects of phosphorylation and mutation are not particularly novel, the quality of the computational work, the application of the method (OneOPES), and the rigour of the methodology prove that the work deserves its place in Nature Communications. This work has proven that rigorous computational work will push the ion channel community forward. Though revision is needed on the following points to strengthen the manuscript.

Major

Given that the finding on the effect of the mutation on the energetic landscape is predictable, I look forward to see how simulations can provide mechanistic insight. Could you explain, or propose why and how the phosphorylation and the mutation structurally affect the up-down conformation distribution?

The standard deviation between 3 repeats of OneOPES is quite large, given that each run has its own minima. Can you explain why that is happening and discuss it further in the discussion? I also find the convergence analysis in this manuscript (Figure 2e) quite difficult to interpret. Is the idea simply that all conformation (ranging from 2 to 9) is being sampled? If that is the case, is the colour bar describing the system's bias to leave the minimum? The range of the colourbar doesn’t seem to be covered - why did we have to go all the way to -50?

My main question will be how movement up and down the allosteric pathway communicates to the selectivity filter. Given

that you managed to sample the basin in both states, can you explore whether there is any allosteric communication between the Ctd to the SF. You also have cases where the mutation and phosphorylation show opposite effects, so it should be possible to describe the rearrangement.
I don't think the effect of the A314P mutation on the dihedral around F284 is that pronounced, as it is very close to the edge. Can you run longer, or quantitatively compare the depth of the energy basin between them to make the point clearer?

Minor

Please fix kj/mol to kJ/mol with capital J for the unit of energy throughout the manuscript.
If stated in the figure legend, such as Fig. 2, the standard deviation across 3 runs should be reported. Otherwise, could it be confused with the standard deviation due to data binning?
I think you can put Suppl. 2 together with Fig. 3 and Suppl. 3 with Fig. 4.
Figure 5D has no replica, and STD is shown. Maybe there is a version error? Can you show all the data, please?
Figures 5e/f have overlapping labels; that needs to be fixed.
Line 354 - Figure 5, what? Seems incomplete.
Line 152 - When compared to the experimental data, can you always justify whether it compares well quantitatively (just the trend), or quantitatively, where the IC50 matches the free energy of the basin?

Reviewer #3

(Remarks to the Author)
The authors use enhanced-sampling molecular dynamics to study the energetic landscape of TREK-2 channels during phosphorylation, stretch and in response to mutations. The energy landscapes are consistent with experimental data, which makes the model a useful tool. But the authors are not pushing the limits of their simulations, in my mind. Some more unexpected findings or novel mechanisms would enhance this manuscript.

Major comments
1. The authors use a morph to find intermediate in their enhanced sampling strategy. How can you know if this CV path is correct or the only one? They use some extra CVs, but are these sampling enough of the conformational space?
2. Line 248-249. "providing a mechanistic basis for allosteric control of SF gating." But what is the mechanism? You only see it happening, but that does not explain why it is happening. You have to either provide some mechanistic insight or tone down your claims.
3. Figure 4. The angle does not change so how can you claim coupling? Is it just the absence of red at -120 degree? Or barrier to flipped different? Maybe you can indicate in the figure where the important changes are that you claim and explain better how that leads to coupling?
4. Line 339. It is not surprising that a proline induces a kink in M4 which leads to GOF? Also, the experimental correlation that a GOF mutation will be less affected by a drug that binds closed state seems simple. Could you provide some more insightful mutational effect? Maybe a de novo mutation that you predict the effect of and then show experimental evidence for that mechanism?
5. Line 350-354. Can you show some actual structural overlays to further strengthen the similarities of your models to the structure?
6. Line 383. "capture the complex allosteric coupling between M4-pCt motions and the SF gating". Did you really show this? You see it happening, but you do not provide the mechanism why it is happening.

Minor comments.
1. Line 153. Explain in main text how stretch was applied. Now only explained in Methods.
2. Line 72-76. This sentence sounds strange to me: "Electron density…made accessible through fenestration". Maybe better to break the sentence up in two parts and explain everything better?
3. Line 186&188. AdA and BdA? Maybe explain better the nomenclature: "Between subunit A and A= AdA" etc.
4. Line 360. Is the figure referred to simulated under stretch or for the mutation? Probably for the mutation. And it is in 5f, not 5e.
5. Line 536. Is the value of stretch you provide consistent with physiological values of stretch? Some reference to this would be good to include.
6. Line 644. Equation is definitely wrong.

Version 1:

Reviewer comments:

Reviewer #1

(Remarks to the Author)
Second Review
The authors have put genuine effort into this revision and several improvements are real and accepted. The structural overlay for A314P with quantified kink angles and contact distances (Supp. Fig. 8, Table 4) is a solid addition. The membrane equilibration data (Supp. Figs. 12–13) address the earlier gap appropriately. The ΔΔG comparison with propagated uncertainties replaces a purely visual argument with something more quantitative, and the decision not to apply a formal statistical test at n=3 is reasonable and honestly explained. The mutation numbering discrepancy is now properly

resolved with the new supplementary table. The revised Fig. 6 legend, replacing "non-conductive" with "initial SF conformational changes," is a meaningful correction. These points require no further attention.

Three concerns remain.

The functional labeling of the FES minima is not independently supported in this manuscript.
The authors describe certain FES minima as corresponding to "conductive" and "non-conductive" SF states, primarily through the ψ-F284 dihedral angle. The new Supp. Fig. 9, which I asked for, shows that S2–S4 ion occupancies and Y175–Y175 inter-subunit distances do not vary substantially across conditions. The authors acknowledge this honestly in the rebuttal and explain that those parameters were not biased during the simulations. The problem is that this acknowledgment does not propagate into the main text. The functional assignment — which FES minimum is conductive and which is not — comes entirely from ref 17, where voltage-driven simulations with real permeation events and full ion occupancy profiles established that correspondence. In the current study there is no voltage, no permeation, and now the additional SF parameters are confirmed to be flat. I am not asking the authors to run permeation simulations — that would be a different study. I am asking them to state clearly in the main text that the functional identity of the FES states relies on the prior work and is not independently demonstrated here. The revised Fig. 6 legend is a step in this direction, but the same distinction needs to be applied consistently throughout results and discussion, wherever the language implies that the simulated states are known to be conductive or non-conductive.

The z-distribution confirms the limitation rather than addressing it.
Supp. Fig. 2 shows that z stays below approximately 0.2 nm^2 throughout all simulations. The authors present this as reassurance that the path CV is adequate. The interpretation is the opposite of what they claim. A system that remains tightly confined to the morphed tube for the entire simulation provides no information about whether that tube is the only relevant conformational pathway, or whether energetically accessible states exist outside it. The multi-thermal replicas did not drive the system off the path in any observable way. The discussion already contains one sentence noting this limitation, which I acknowledge. But the abstract and results still present the FES as a characterization of the TREK-2 conformational landscape, when what is characterized is the energetics along a predefined axis between two known crystal structures. These are not the same thing, and the text should say so.

The circular argument in Section IV persists in the main text.
The rebuttal correctly acknowledges that the co-variation between the path CV and the M4–M2 auxiliary CVs is "a geometric consequence of the path CV construction." This was the right response to my concern. However, the revised main text immediately follows that acknowledgment with the statement that "M4-pCt motion both shifts the thermodynamical equilibrium of the SF and modulates the local barrier associated with carbonyl flipping, thereby providing an energetic basis for the thermodynamic coupling." This sentence re-introduces the causal claim through the back door. A reader of the manuscript who has not read the rebuttal will encounter the acknowledgment and the causal claim in the same paragraph and will not see the contradiction. What the FES shows is a thermodynamic association between two coordinates that are partially linked by the geometric definition of the path. This is a useful result. It is not, however, evidence that M4 motion causes SF rearrangement. The language in the main text should consistently reflect the former rather than the latter.

One minor point.
The AlphaFold3 prediction used to justify the helical pCt conformation (Supp. Fig. 10) appears to fall in the pLDDT range of 50–70 for the relevant segment. This range is classified by AlphaFold itself as low confidence. The authors should state the pLDDT value explicitly and acknowledge that the helical conformation of the modelled pCt remains a working assumption, not a structurally validated feature.

Reviewer #2

(Remarks to the Author)
I thank the authors for their rigorous revision of the manuscript. The paper is now ready for publication.

Reviewer #3

(Remarks to the Author)
The authors have responded well to my comments.

Open Access This Peer Review File is licensed under a Creative Commons Attribution 4.0 International License, which permits use, sharing, adaptation, distribution and reproduction in any medium or format, as long as you give appropriate credit to the original author(s) and the source, provide a link to the Creative Commons license, and indicate if changes were made.
In cases where reviewers are anonymous, credit should be given to 'Anonymous Referee' and the source.
The images or other third party material in this Peer Review File are included in the article's Creative Commons license, unless indicated otherwise in a credit line to the material. If material is not included in the article's Creative Commons license and your intended use is not permitted by statutory regulation or exceeds the permitted use, you will need to obtain permission directly from the copyright holder.
To view a copy of this license, visit https://creativecommons.org/licenses/by/4.0/

We would also like to express our sincere gratitude to all three reviewers for their thorough and highly valuable feedback and suggestions, which we found essential for improving our manuscript. We have carefully addressed all comments and incorporated the suggested changes throughout the revised manuscript. Enclosed below are our detailed, point-by-point responses to the reviewers' feedback. All modifications in the manuscript have been highlighted.

REVIEWER COMMENTS

Reviewer #1 (Remarks to the Author):

Energetic cross-talk of filter gate and lower helices drives polymodal regulation and disease in TREK K2P channels

The manuscript addresses an important biological question — how phosphorylation, mechanical stretch, and a disease-associated mutation reshape the conformational landscape of TREK-2 — and the OneOPES enhanced sampling framework is in principle well-suited to this problem. The electrophysiological validation of the A314P mutant using NFx inhibition is a strength. However, after careful reading of the main text and supplementary material, I have identified a series of problems that range from factual inaccuracies and missing controls, to a systematic pattern of overclaiming that I will detail below. In my view, the manuscript requires major revision, with additional simulations and analyses, before it can be considered for publication. I will organize my remarks by category.

I. Factual errors and presentation problems

At line 84, the text refers to "Fig. 1b," which does not exist. Fig. 1 contains only panel (a). Either the reference is wrong, or a panel was removed during revision without updating the text. This must be corrected.

Response: We thank the reviewer for noticing this inconsistency. The reference to Fig. 1b originated from an earlier version of the figure that schematically illustrated the up-to-down conformational changes. In the last version, this panel was removed, but the in-text reference was mistakenly retained. We have now removed the reference to Fig. 1b throughout the manuscript, and Fig. 1 now contains a single panel.

At line 360, the text discusses SF stability for the A314P mutant and cites Fig. 5e, which shows M4-M2 distances. The relevant panel for SF stability is Fig. 5f. This is an error.

Response: Thanks for noting this mistake. We have corrected it now.

At line 487, "pays the way" should read "paves the way."

Response: We corrected it.

Lines 57-58 describe two FHEIG mutations in TRAAK, A244P and A172E. Later, lines 299-300 introduce A270P in TRAAK and its TREK-2 homologue A314P as the basis for the simulations. These are different mutations from different patients. The transition between them is never explicitly explained, and a reader who is not a specialist in this disease will be confused. Please clarify which mutation is being studied computationally and why, and verify the genomic and structural assignments at lines 57-58 against the primary sources.

Response: We agree with the reviewer. The discrepancy arises from the use of two different numbering systems in the literature. Bauer et al. (2018) report the FHEIG-associated mutations using the UniProt numbering of full-length human TRAAK (Q9NYG8), giving A244P and A172E. The crystallographic TRAAK structures (PDB IDs: 4WFE, Brohawn et al. 2014; 7LJ4, Rietmeijer et al. 2021) instead use the numbering of the crystallization construct, in which the equivalent residues are A270 and A198. For the present computational work, we modelled the TREK-2 homologue of TRAAK A270 (i.e. A244 in UniProt numbering), which corresponds to A314 in the TREK-2 sequence (PDB ID: 4BW5). We have now clarified this issue at its first occurrence in the main text and added a Supplementary Table summarizing the residue mapping between TRAAK UniProt (Q9NYG8), TRAAK 4WFE, and TREK-2 4BW5 numbering scheme.

Supplementary Table 1. Correspondence between TRAAK and TREK-2 residue numbering systems for the FHEIG mutations

Mutation	TRAAK UniProt (Q9NYG8)	TRAAK PDB 4WFE	TREK-2 PDB 4BW5	Source	Used in this study
FHEIG mutation 1	A244P	A270P	A314P	Bauer et al. 2018	Yes (modelled in TREK-2)
FHEIG mutation 2	A172E	A198E	T241	Bauer et al. 2018	No

II. Modelling choices that are either unjustified or misleading

The pCt was modelled computationally using PyMOL builder with a forced helical conformation (line 497). The legend of Fig. 1 labels these structures "X-ray extended," which strongly implies experimental determination. This is incorrect and misleading. The label should read something like "computationally modelled pCt" and the methods section should provide the structural or biochemical justification for imposing an α-helical conformation. Citing the authors' prior work is not sufficient — the original justification for this choice needs to appear somewhere.

Response: We agree with the reviewer and have revised the legend as: "*Fig. 1. Conformational states and constructs of TREK-2 K2P channels used in the present MD study. Crystal structures of TREK-2 in the up-state (PDB ID: 4BW5) and down-state (PDB ID: 4XDJ) with the 19-residue pCt modelled in an α-helical conformation (designated TREK-2*; see Methods), along with one*

representative final snapshot from our previous unbiased MD simulations17. The PKC phosphorylation site S331 within the pCt region and the investigated A314P mutant site are highlighted by yellow and orange boxes, respectively. Key conformational rearrangements of the SF residues F284 and Y175 suggested by our previous unbiased MD simulations are highlighted in cyan and purple, respectively."

In addition, we have expanded the corresponding paragraph of the Methods:" *The missing pCt region in each subunit was manually extended using the PyMOL builder tool. A total of 19 residues (333–351: TKEEVGEIKAHAAEWKANV), corresponding to the pCt extension of the TREK-2* construct used in our previous study, were modelled in an α-helical conformation. The choice of this initial α-helical starting conformation is supported by AlphaFold3 predictions of the full-length TREK-2 structure (Supplementary Fig. 10). Additionally, missing loop segments (residues 149–154 and 229–235) were modelled using SWISS-MODEL."*

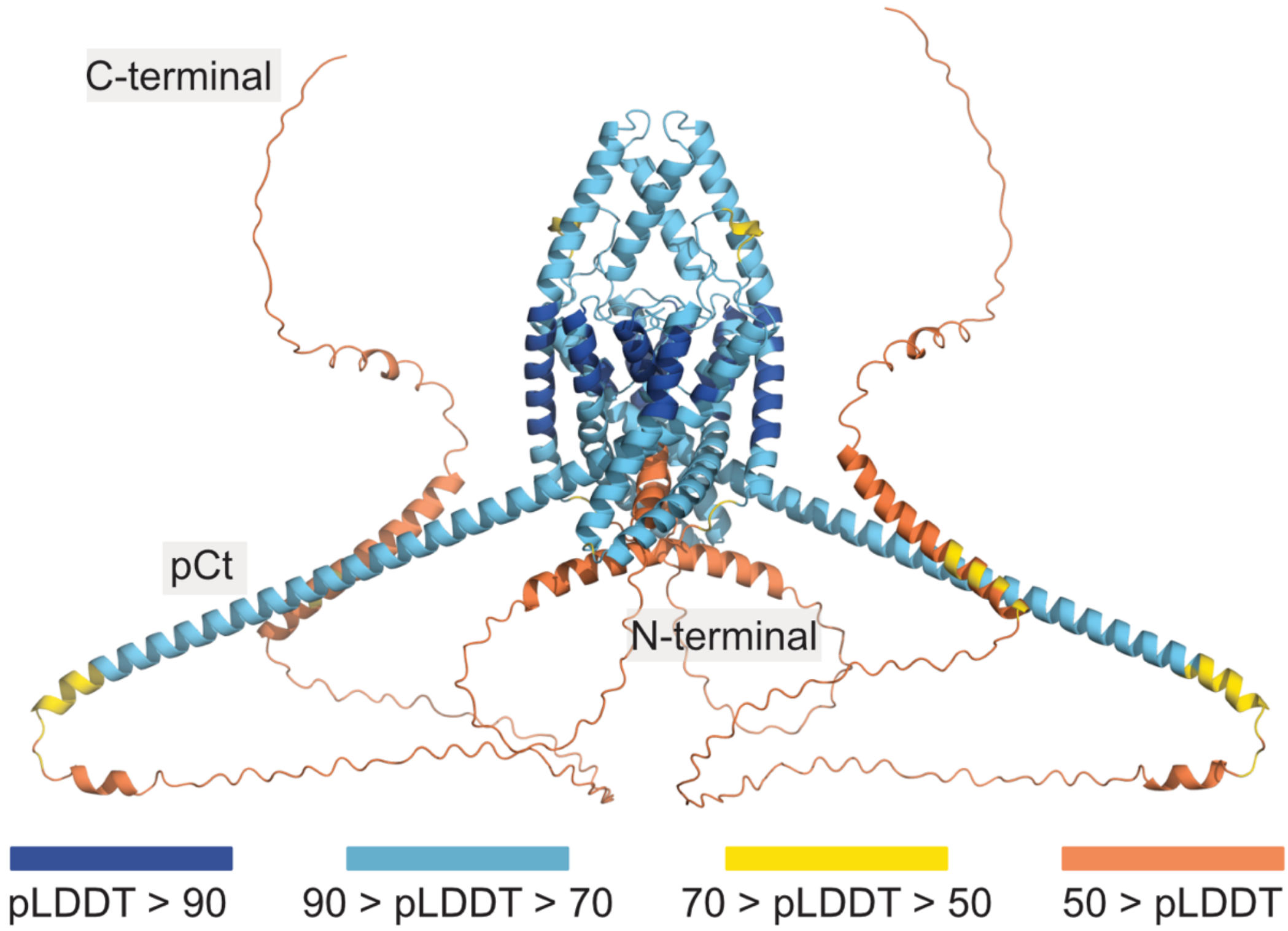

Supplementary Fig. 10. Predicted full-length structure of human TREK-2 channel using AlphaFold3.

The membrane is composed exclusively of POPC (line 504, 295 lipids). Given that the fenestration-based lipid-block mechanism is central to the discussion of TREK gating, and given the known effects of phosphoinositides and other anionic lipids on K2P channels, a POPC-only bilayer is a significant simplification. The authors cannot address lipid specificity with this setup, and should at minimum acknowledge this as a limitation.

Response: We thank the reviewer for this remark. We have added the following discussion now into the text: *"A further consideration concerns the lipid composition of our simulation systems, which consists exclusively of POPC. This simplification was intentionally adopted to isolate the intrinsic protein-driven energetics of the M4–pCt–SF coupling from the additional complexity induced by lipid-specific modulation. It has been previously shown that TREK channels are modulated by anionic lipids, most notably $PI(4,5)P_2$ and other phosphoinositides,*

as well as by polyunsaturated fatty acids such as arachidonic acid. A simple POPC bilayer cannot capture lipid-specific effects on the conformational equilibrium. The free-energy differences reported here should be therefore interpreted as the intrinsic response of the protein to phosphorylation, membrane stretch, and mutation within a simplified model membrane environment."

The KCl concentration of 600 mM (line 505) is justified by reference to prior unbiased simulations where accelerating ion conduction was the goal. However, in the present study the sampling is already accelerated by OneOPES. The original justification no longer applies with the same force. Given that the pCt carries multiple charges and the phosphoserine is modelled at -2, a concentration three to four times above physiological is not trivially innocuous, while commonly used in MDs. The authors should discuss whether this choice affects the relative stability of up versus down states.

Response: The 600 mM KCl concentration was retained to enable direct comparison with our previous unbiased MD simulations of TREK-2, where, as noted by the reviewer, elevated KCl concentrations were used to accelerate ion conduction under applied transmembrane voltage. We agree, however, that this original rationale does not apply with the same strength in the present OneOPES simulations, where no voltage was imposed. We have now clarified this point in the Discussion as follows: "*A further consideration concerns the ionic strength of our simulation systems, which was set to 600 mM KCl to maintain direct comparability with our previous unbiased MD simulations of TREK-2[17], where elevated K^+ concentrations were employed to accelerate ion conduction under transmembrane voltage. As the pCt contains multiple charged residues, including the phosphoserine modelled here in its doubly deprotonated state (−2 charge), elevated ionic strength could in principle influence the computed free-energy landscape through electrostatic screening effects. Nevertheless, the predicted conformational equilibria qualitatively agree with available experimental observations regarding the effects of phosphorylation, membrane tension, and state-dependent inhibitor binding in the mutant systems. Future work could systematically investigate the influence of ionic strength on the computed free-energy surfaces.*"

The equilibration validation is limited to RMSD and temperature (Supp. Fig. 4). No membrane area per lipid, bilayer thickness, or lateral pressure profile is reported. These are standard validators for membrane systems and their absence makes it impossible to judge whether the initial membrane density was appropriate, or whether the choice to restrict cell fluctuations to the z-direction (line 516) was justified for this particular system.

Response: We have now included in the Supplementary Information the time evolution of the area per lipid and bilayer thickness (P–P distance of the simulation box for the apo, phosphorylated, stretch-activated, and A314P systems (Supplementary Fig. 12, 13). The mean area per lipid stabilizes at 69 Å for apo, phosphorylated, and A314P systems, while it expands to 86 Å under applied lateral tension, as expected. The bilayer thickness is 39 Å without stretch and 32 Å under stretch. Together, these analyses indicate that the bilayers reached well-equilibrated states.

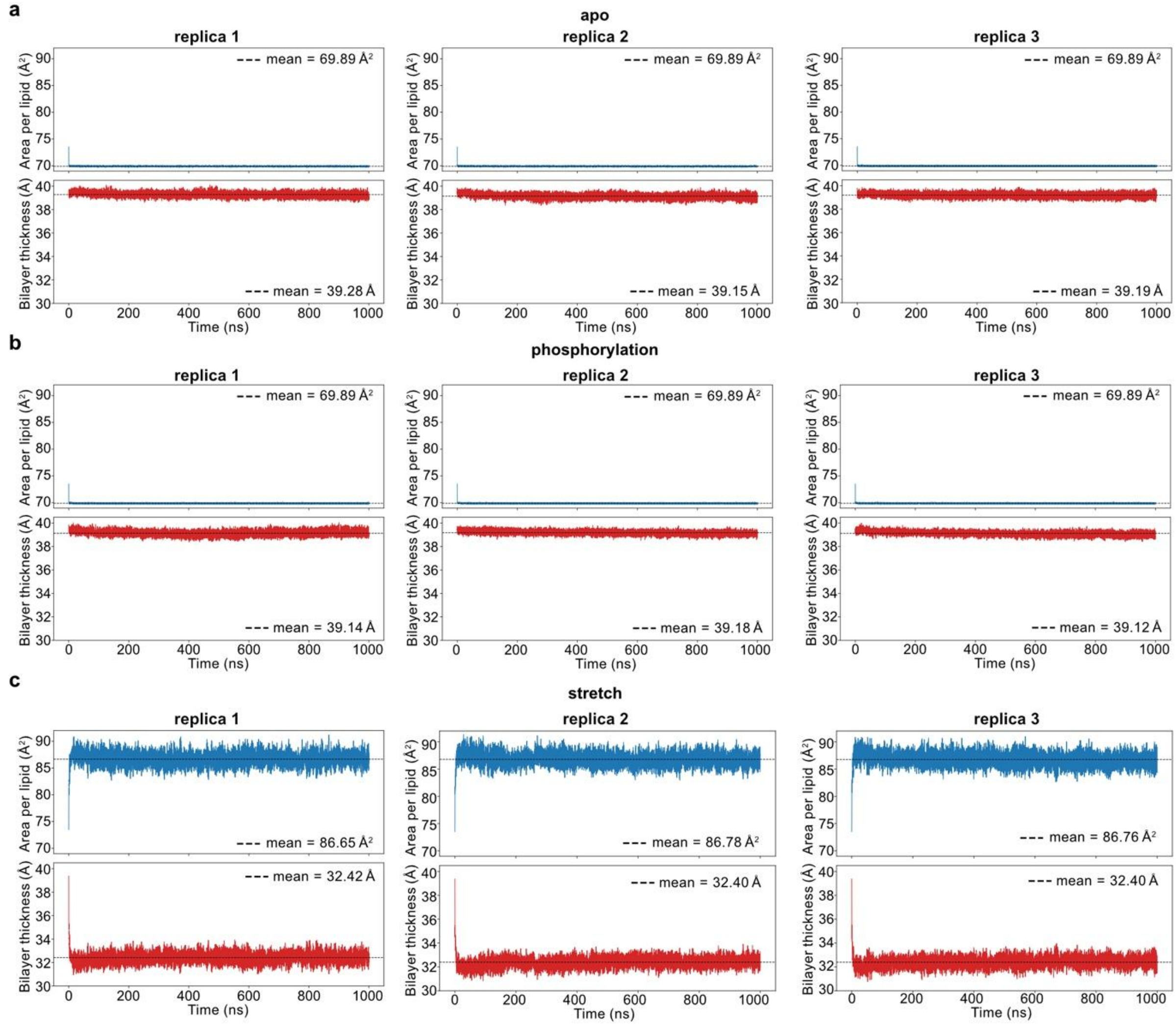

Supplementary Fig. 12. Bilayer integrity assessment for the apo, phosphorylated, and membrane-stretched TREK-2* OneOPES simulations. Area per lipid (APL, upper) and phosphate–phosphate (P–P) bilayer thickness (lower) computed for the **(a)** apo**, (b)** phosphorylated, and **(c)** membrane-stretched systems. For each system, three independent replicas are shown. APL was calculated by dividing the instantaneous bilayer (xy) area by the number of POPC molecules per leaflet, and P–P thickness was computed as the center-of-mass distance along the membrane normal between the phosphorus atoms of the two leaflets, both sampled every 100 ps. The apo and phosphorylated systems were simulated under standard semi-isotropic pressure coupling (+1 bar in the xy-plane, +1 bar along z); the membrane-stretched system was simulated with a lateral pressure of −50 bar in the xy-plane while the normal pressure was maintained at +1 bar. Under applied tension, the APL increases and the P–P thickness decreases relative to the unstretched apo and phosphorylated references, in agreement with the expected response of POPC bilayers to lateral stretch, and both observables stabilize around new mean values that remain constant across the remainder of the trajectories. No membrane rupture or pore formation was observed in any replica.

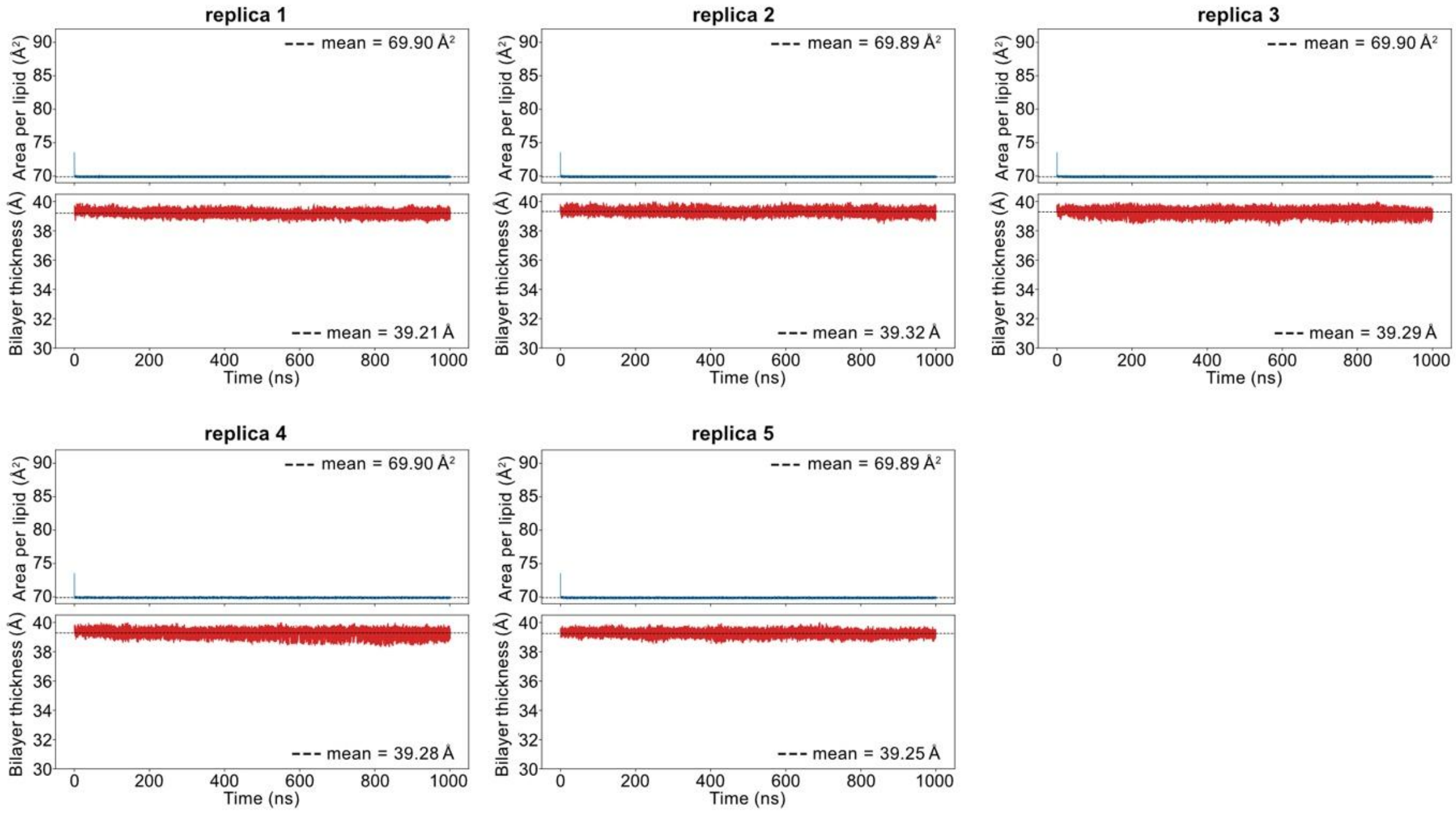

Supplementary Fig. 13. Bilayer integrity assessment for the A314P disease-mimicking TREK-2* OneOPES simulations. Area per lipid (APL, upper) and phosphate–phosphate (P–P) bilayer thickness (lower) computed for the five independent replicas of the A314P TREK-2* system. APL and P–P thickness were calculated as described in Supplementary Figure 12, with semi-isotropic pressure coupling at +1 bar in both the xy-plane and along z. Both observables remain stable around their respective means throughout the production phase across all five replicas, and the values are comparable to those obtained for the apo system (Supplementary Figure 12a), supporting that the bilayer is structurally intact under the conditions used to evaluate the disease-mimicking mutation.

III. The path CV: a coordinate is not a pathway, but its limitations must be addressed

The authors construct the path collective variable using 10 structures generated by PyMOL morphing between the up and down crystal structures, equally spaced in RMSD (lines 553-555). I want to be precise here, because this point is often misunderstood. The morph structures are reference points that define a progress coordinate s — they do not constrain the trajectory to follow this interpolated path. I understand this. Nevertheless, there are two problems that are not addressed.
First, the Branduardi path CV also defines a distance-from-path variable z, which measures how far the instantaneous conformation is from the interpolated tube. This quantity is never shown, neither in the main figures nor in the supplement. Without it, the reader cannot know whether the system remains close to the morphed path or whether it explores conformations that are geometrically distant from all 10 reference models. If z is large, the s values reported in the FES are ambiguous projections. They are rather ambiguous as progress coordinates. The authors must show the z distribution, or provide an explicit argument for why this is not necessary.

Response: We thank the reviewer for raising this important point. The distance-from-path variable z was monitored throughout all OneOPES simulations and is now reported in a new Supplementary Figure (Supplementary Fig. 2). Across all systems and replicas, the marginal distribution of z is sharply peaked, with the 95th percentile remaining below ~0.2 nm² (mean squared displacement units), demonstrating that the sampled conformations remain tightly confined to the interpolated tube defined by the ten morph structures. Within this regime,

the instantaneous values of s can be safely interpreted as well-defined progress coordinates along the path, rather than ambiguous projections onto a poorly resolved transition tube. We have additionally included both the time evolution and the one-dimensional distribution of z for all systems in Supplementary Figure 2. Further text has been added into the main text: "*Convergence analysis of the OneOPES bias potential, together with the time evolution of the path CV further supports the robustness of sampling along the path CV across all replicas (Fig. 2e and Supplementary Fig. 1, 2).*"

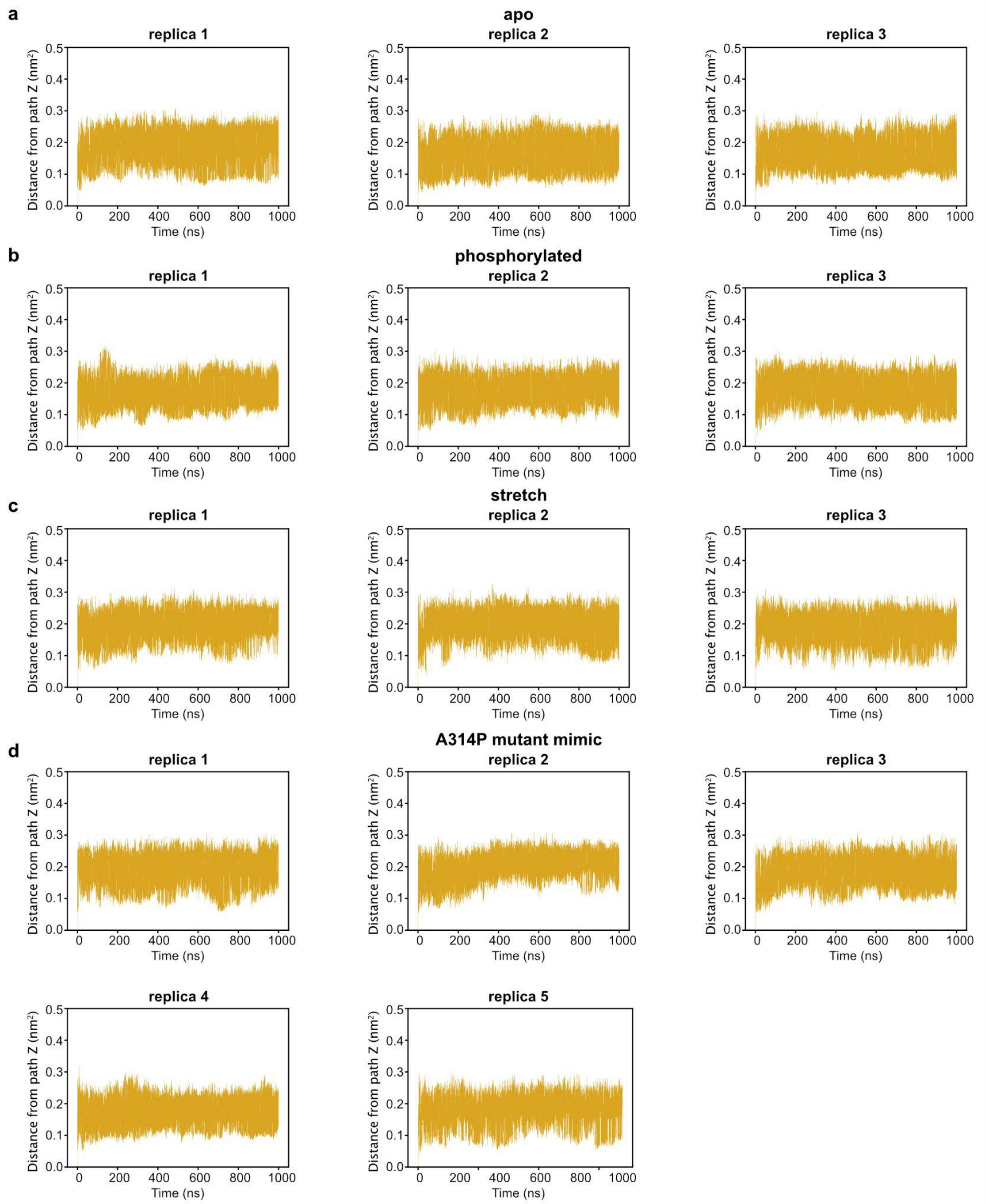

Supplementary Fig. 2. Time evolution of the distance-from-path variable z. Timewise plots for all replicas across the four systems: **(a)** apo, **(b)** phosphorylated**, (c)** membrane-stretch, and **(d)** disease-mimic mutant. The variable Z represents the RMSD distance from the predefined path collective variable S.

Second, and more fundamentally: the path CV biases exploration along the predefined up↔down axis. Any conformation that lies off this axis — a third basin, a metastable intermediate not present in either crystal structure, a conformation accessible under physiological conditions but geometrically distant from the morph interpolation — will not be efficiently promoted, because the bias along s provides little driving force for off-path exploration. The multi-thermal replicas partially compensate for this, but not in a targeted way. This is a statement about what any path-based CV can and cannot do. The authors never acknowledge this limitation, and they should.

Response: We agree that a general limitation inherent to path-based collective variables is that they bias sampling along a predefined axis between two reference conformations and do not, by themselves, provide a directed driving force for exploring regions of configurational space that are geometrically distant from the interpolated path. As the reviewer noted, this limitation is partially mitigated by the OneOPES architecture, in which the multi-thermal replicas facilitate barrier crossing along orthogonal degrees of freedom. In addition, the auxiliary CVs describing SF dihedrals and M4–M2 displacements enable a degree of independent sampling of these motions beyond the progress along the path CV itself. Furthermore, when projecting the unbiased apo trajectories onto different CVs (Supplementary Fig. 5), we see that the OneOPES simulations recapitulate highly similar regions of conformational space sampled in the unbiased MD. Nevertheless, we agree that the ensembles presented here should be interpreted as an energetic description of the up-to-down conformational transition, rather than a global exploration of the TREK-2 conformational space. We have included the following text in the results section: "*As with other path-based enhanced sampling approaches, the CV is designed to describe the essential dynamics connecting these predefined reference states and therefore does not efficiently explore conformations that lie far from the interpolated transition pathway.*"

The practical consequence is the following. Several conclusions in the manuscript — that the system occupies two and only two relevant states, that the FES minima are genuine thermodynamic basins rather than artifacts of the coordinate definition, that the SF conformation observed in those minima is stable — cannot be fully verified from the biased simulation alone. The most direct and least expensive way to address this is to perform short unbiased MD simulations, of the order of 300-500 ns, initiated from the FES minimum structures for each condition. Here the target are stability/verification runs, three replicates allowing some basic variance assessment. If those structures are genuinely stable, the SF conformation, the M4 position, and the key CV values should be maintained without bias. If they are not, the FES minima do not correspond to physically meaningful states.

Response: We agree that unbiased MD simulations provide a direct and independent assessment of whether the minima identified in the FES correspond to physically meaningful conformational states. In our previous work, we already performed unbiased MD simulations of the apo up- and down-states, consisting of five independent 1 µs replicas initiated from each crystallographic state. During the present revision, we projected these trajectories onto the OneOPES collective variables (Supplementary Fig. 5). This analysis shows that the unbiased trajectories remain predominantly confined to the up- and down-state regions that populate regions very similar to the corresponding basins resolved in the FES. In addition, limited

sampling of intermediate regions is observed, consistent with the relatively shallow one- and two-dimensional free-energy surfaces (Figs. 2b and 3b). Although none of the apo replicas undergo a complete up-to-down or down-to-up transition within the unbiased simulation timescale, one trajectory initiated from the down-state samples conformations approaching the up-state basin. This observation is consistent with our interpretation that both conformational states are thermally accessible in the apo system, likely on timescales somewhat beyond those reached in our unbiased microsecond simulations. We have now included this reanalysis of the unbiased MD simulations in the revised manuscript: "*Independent support for the physical relevance of the up- and down-state minima from OneOPES comes from our previous unbiased simulations, in which the up- and down-state ensembles, when projected onto the path CV, populate regions similar to the corresponding basins resolved in the FES (Supplementary Fig. 5 a,b).*"

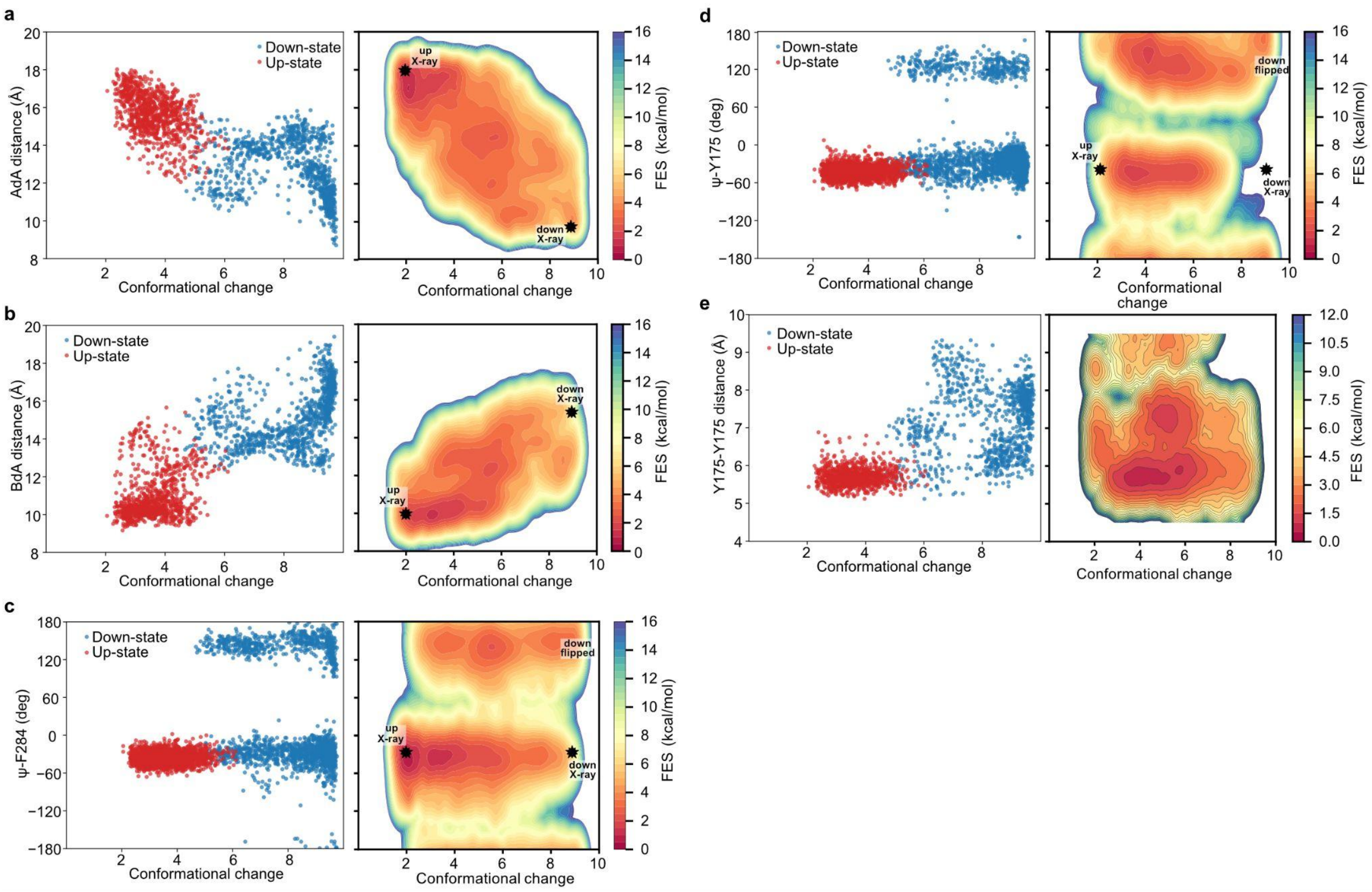

Supplementary Fig. 5. Projection of unbiased up- and down-state TREK-2* simulations onto the OneOPES collective-variable space, shown alongside the corresponding apo OneOPES free-energy surfaces. In each panel, the left plot shows conformations sampled in our previous unbiased MD simulations (ref. 17), five independent 1 μs replicas started from the up-state (red) and five from the down-state (blue), projected onto the collective variables used in the OneOPES setup and plotted against the path CV (s, "conformational change" on the x-axis), which describes the progression from the up-state (low s) to the down-state (high s). The right plot shows the two-dimensional free-energy surface (FES) reconstructed by reweighting the apo TREK-2* OneOPES ensemble (average of three independent replicas) projected along the path CV and the same variable, allowing a direct comparison between the regions spontaneously sampled in the unbiased ensemble and the energetic minima recovered by enhanced sampling. **(a)** Intra-chain M2–M4 distance (AdA, subunit A M2 to subunit A M4). **(b)** Inter-chain M4–M2 distance (BdA, subunit B M4 to subunit A M2). **(c)** Backbone ψ dihedral angle of F284 and **(d)** of Y175, both reporting on the SF conformation at the S1 K^+ binding site. **(e)** Inter-subunit Y175–Y175 backbone carbonyl distance, computed between the centres of mass of the backbone carbonyl atoms (C and O) of Y175 in the two opposing subunits. Black stars indicate the positions of the crystallographic TREK-2 up- and down-state structures mapped onto these coordinates; the "down flipped" label marks the flipped-carbonyl SF conformation accessible in the down-state region. In every panel, the unbiased up- and down-state ensembles partition into distinct regions of the path CV that coincide with the up- and down-state minima of the apo OneOPES FES. Free energies are given in kcal/mol.

IV. Circular reasoning in the validation of auxiliary CVs

The correlation between the path CV and the M4-M2 auxiliary CVs (AdA, BdA) is presented as validation that the auxiliary CVs "faithfully track the structural rearrangements that accompany the global gating motion" (lines 193-195). This is not independent validation. The path CV was constructed from the RMSD of Cα atoms between the up and down crystal structures, and M4 undergoes the largest displacement between those two structures. Any coordinate that measures M4 position will correlate with s by geometric necessity. The paragraph at lines 190-208 does not go beyond what was already implied by the path construction. The authors should either provide genuinely independent validation — for example, using a structural observable that was not part of the path definition — or remove the claim of validation and rephrase this section as a description of what the auxiliary CVs capture.
The same logic applies to lines 231-233, where the correlation between path CV and ψ-F284 is described. The path CV encodes the global structural difference between up and down, which includes the SF environment. A correlation between s and ψ-F284 does not establish a mechanical link between M4 position and SF dihedral.

Response: We agree with the reviewer that the observed correlation between the path CV and the auxiliary CVs largely reflects the geometric construction of the path construction and therefore should not be interpreted as an independent validation of the path CV itself. The primary purpose of the auxiliary CVs is to provide efficient driving forces along the specific slow modes that define the global transition. The argument to include them as auxiliary CVs becomes more evident when looking at the newly included Supplementary Fig. 5a,b from unbiased trajectories above. While AdA and BdA are clearly correlated to the path CV, and the up and down states separate cleanly in both cases (each occupy their own region in 2D space), the "path" the trajectories take are somewhat curved. For such cases, convergence will usually be improved when they are included as separate CVs in biasing.

This curvature is somewhat replicated in the 2D free energy surfaces, though we acknowledge it is difficult to estimate how significant it is in the transition region (Supplementary Fig. 5 a,b). The spread of the AdA and BdA values at fixed path CV in the unbiased data and the correspondence between the unbiased data and the FES suggests that the AdA and BdA CVs retain some genuine conformational freedom orthogonal to the path CV, and that this is also sampled in the biased simulations.

We have now revised the relevant paragraphs to clarify that the auxiliary CVs were introduced to improve sampling efficiency and convergence of the simulations, rather than to serve as an independent validation of the path CV:

"Across all systems, the 2D FES (Fig. 3) show a strong correspondence between the M4–M2 displacement coordinates and the path CV: low path CV values (up-state) coincide with high AdA and low BdA, whereas high path CV values (down-state) display the opposite configuration (Fig. 3b and Supplementary Fig. 4). This co-variation can be considered as a geometric consequence of the path CV construction, since the up-to-down transition is dominated by M4 motion. Accordingly, the AdA and BdA auxiliary CVs decompose the global progress coordinate s into the specific intra- and inter-subunit M4–M2 displacements that

drive the transition. Their role within the OneOPES setup is to provide additional driving forces along these slow, sub-unit-coupled motions."

"*Together, these results show that the path CV, decomposed by the M4-related auxiliary CVs, captures the global structural rearrangements of TREK-2 across different regulatory conditions and reveals phosphorylation and membrane stretch as opposing modulators along a common energetic axis*."

"*Here, M4-pCt motion both shifts the thermodynamical equilibrium of the SF and modulates the local barrier associated with carbonyl flipping, thereby providing an energetic basis for the thermodynamic coupling of M4–pCt dynamics to SF gating. We acknowledge that part of the observed co-variation between the path CV and the SF dihedral observables arises from the geometric consequence of the path CV construction itself, as the up- and down-state crystal structures used to define the path may slightly differ in their SF environments, even though both crystallographic states adopt nearly the same conductive SF conformation.*"

V. The allosteric coupling claim is not supported by the data

This is the most serious problem in the manuscript, and it recurs at lines 247-249, 262-263, 288-293, 451-453, 471-473, and 484-485. In each case, the authors observe that under a given condition (phosphorylation, stretch, mutation), both the M4-pCt region and the SF adopt a particular configuration in the FES, and from this they conclude that M4-pCt motion "allosterically controls" or "directly controls" the SF gate.
This reasoning is not sufficient to establish a casual link. The up and down crystal structures differ globally — not only in M4 position, but also in the environment of the SF, in inter-helix contacts, in pore geometry, and in many other coordinates. When the system is in the down-state region of the FES, the SF is in a particular conformation not because M4 has acted on it allosterically, but because the entire protein is in a different global state. To claim allostery, one needs to isolate the M4-SF coupling from the global structural change. This could be done, for example, by showing that constraining M4 in the up-position while the rest of the protein is in a down-like conformation still stabilizes the canonical SF — or by performing unbiased simulations starting from a down-state structure under stretch conditions and observing whether M4 moves before or after the SF changes. Neither analysis is present.
I would ask the authors to either remove the word "allosteric" throughout, replacing it with more conservative language about co-variation or thermodynamic association, or to provide the additional data needed to support a causal claim. The latter would substantially strengthen the paper.

Response: We thank the reviewer for this thoughtful suggestion and would like to clarify our interpretation of this point. Although phosphorylation occurs at a PKC site within the pCt, spatially distant from the SF, our previous unbiased simulations showed that phosphorylation reproducibly induces carbonyl flipping at S1 binding site of the filter when starting from the up-state, whereas the corresponding non-phosphorylated simulations remain stable in the conductive SF configuration. This interpretation is now further supported by the OneOPES free-energy calculations, in which the phosphorylated system exhibits the strongest thermodynamic preference for the flipped F284 conformation among all simulated conditions. We consider this data together as good evidence for a causal coupling between the conformational state of the M4/pCt region and the SF. We fully agree that the underlying

process is not mediated by motion of M4 alone, but instead involves concerted rearrangements across multiple regions of the protein. Nevertheless, because the phosphorylation site is located directly adjacent to the M4 helix, we consider changes in M4/pCt orientation to be the most immediate structural consequence of phosphorylation. These rearrangements are then propagated through the protein and ultimately alter the conformational equilibrium of the SF. We acknowledge that this represents a complex and highly dynamic coupling process. In the revised version, we have minimized the use of the term "allosteric" throughout the manuscript and added a short section emphasizing that the proposed coupling between the upper part of the filter and the M4 is supported by combined insights from both unbiased and enhanced sampling simulations, while acknowledging that each approach has its own strengths and limitations when used independently to dissect the underlying mechanism: "T*ogether with our previous unbiased MD simulations, the free-energy landscapes obtained from OneOPES provide a more coherent energetic framework for understanding how chemical, mechanical, and mutational perturbations modulate TREK-2 function through shared structural pathways and define the channel's gating landscape. These findings are consistent with a very recent study from some of us, in which electrophysiology measurements suggested that diverse physiological stimuli activate TREK/TRAAK channels through a common mechanistic pathway. Importantly, the unbiased MD simulations were essential for identifying the complex interaction network coupling the pCt/M4 region to the SF and for defining the key structural descriptors that best characterize the essential conformational transition. In contrast, the state-of-the-art enhanced sampling approach applied here was crucial for quantifying the free-energy changes associated with these transitions and for determining how they are reshaped by different physiological perturbations. Together, these results highlight the complementary strengths of unbiased and enhanced sampling MD approaches in dissecting regulatory mechanism*s *in complex biological systems and underscore the importance of their integration.*"

VI. The selectivity filter characterization is insufficient

The SF state is characterized in this study almost entirely through the ψ-F284 dihedral angle. This is a very limited proxy. In the K2P literature, SF state is typically assessed using a combination of parameters: ion occupancy at all binding sites (S1-S4), inter-subunit distances between conserved tyrosines (Y175-Y175 in this case), carbonyl orientations at multiple filter positions, pore hydration and dewetting, and backbone flexibility. Of these, only K+ coordination at S1 (CV6) and ψ-Y175 are additionally reported, and the Y175 results are acknowledged to be unreliable (lines 252-259). The S2-S4 ion occupancies are not reported. The Y175-Y175 inter-subunit distance, a classical indicator of SF collapse in K2P channels, is absent. Pore hydration is absent.

Response: We agree that the SF requires a more comprehensive characterization than ψ-F284 alone in a posteriori calculations on the reweighted ensemble. In addition to ψ-F284, ψ-Y175, as well as the K^+ and water coordination at the S1, we have now analyzed several additional SF-related descriptors, including the Y175-Y175 distance and S2-S4 ion occupancies (Supplementary Figure 9). Across the different systems, however, these observables did not exhibit substantial changes associated with the global conformational transition. Most likely, this reflects the fact that these additional SF descriptors were not explicitly biased during the simulations, while biasing the local rearrangements at the S1 binding site is insufficient to drive the full range of SF dynamics. A complete characterization of the SF gating process is

beyond the scope of the current OneOPES setup and will likely require the incorporation of additional carefully selected CVs, guided either by long-timescale unbiased MD simulations and/or experimental observables. We have now included these new analyses together with a corresponding discussion in the revised manuscript: "*Nonetheless, our OneOPES simulation results were unable to resolve the complete SF gating procedure. A posteriori analysis of the reweighted ensembles (Supplementary Fig. 9) showed that S2-S4 ion occupancies do not vary substantially across the different simulation conditions. This most likely reflects the fact that these additional SF descriptors were not explicitly biased during the simulations, whereas biasing only the local rearrangements at the S1 binding site is insufficient to drive the full range of SF dynamics at the current timescales.*"

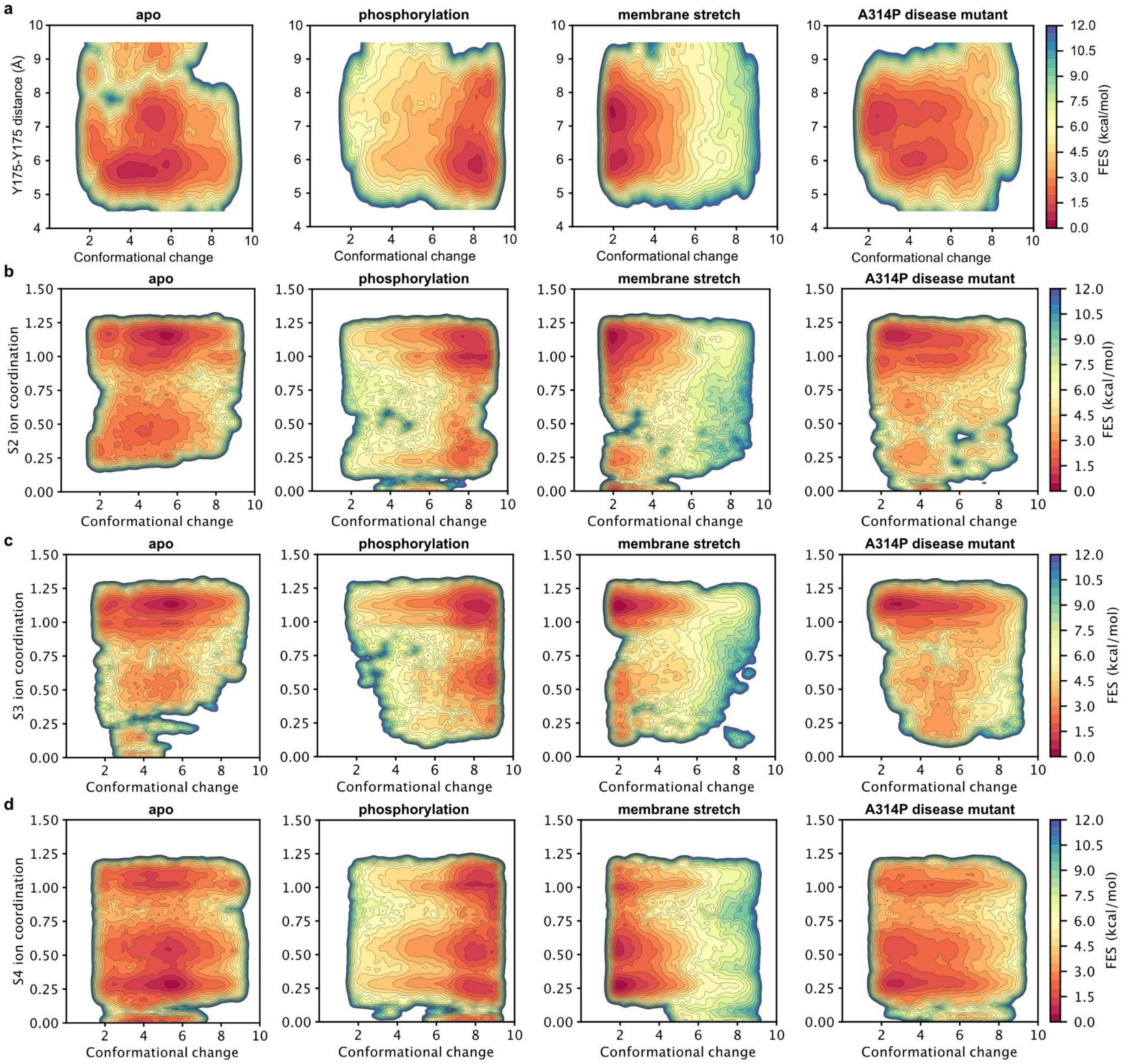

Supplementary Fig. 9. Multi-parameter characterization of the selectivity filter from the OneOPES simulations. Two-dimensional free-energy surfaces projected along the path CV ("conformational change"; up-state ≈ 2, down-state ≈ 8) and four additional selectivity-filter descriptors, reweighted from the OneOPES ensembles. **(a)** Inter-subunit Y175–Y175 Cα–Cα distance (Å), reporting on lateral breathing of the filter. **(b–d)** K^+ coordination number at the (b) S2, (c) S3 and (d) S4 binding sites, each defined as the coordination number between K^+ and the center of mass of the Cα atoms of the four residues lining the corresponding site. In each row, columns correspond, from left to right, to the apo, phosphorylated, membrane-stretch and A314P systems; each surface

is the average of three independent OneOPES runs (five for A314P). The colour scale denotes the free energy in kcal mol^{-1}.

Figure 6 presents a clear and appealing narrative in which up = conductive SF and down = non-conductive SF. But this narrative rests almost entirely on ψ-F284, and even there it is complicated: in Fig. 4b (apo), a metastable flipped-SF conformation is clearly visible within the up-state region of the FES, which the authors acknowledge at lines 236-237 but then contradict at lines 417-418 where they write that "the SF remains in a stable, conductive configuration" in the up-state. These two statements cannot both be true. The authors should reconcile them and provide a more complete characterization of the SF in each FES minimum, using the additional parameters listed above.

Response: We thank the reviewer for flagging this inconsistency. The sentence '*within these conformations, the SF remains in a stable, conductive configuration as revealed in the crystal structure*' has been replaced by: '*Within the up-state basin, the conductive crystallographic SF configuration represents the dominant thermodynamic minimum, whereas a secondary carbonyl-flipped conformer of F284 is separated by a substantial free-energy barrier. As a result, this flipped state was not sampled in our previous unbiased simulations of up-state on the μs timescale, but became resolved in the present OneOPES simulations.*' We have also revised Figure 6 and changed "non-conductive" to "initial SF conformational changes".

The SF state is characterized in this study almost entirely through the ψ-F284 dihedral angle, with partial and acknowledged-unreliable contributions from ψ-Y175. This is a very limited proxy for a structure as functionally complex as the K2P selectivity filter. In the literature, SF state is typically assessed using a combination of parameters: ion occupancy at all binding sites (S1-S4), inter-subunit distances between conserved tyrosines — here Y175-Y175 — which report on SF lateral breathing and collapse, carbonyl orientations at multiple filter positions, pore hydration and dewetting transitions, and backbone flexibility. Of these, only K+ coordination at S1 (CV6) is additionally reported, and the Y175 dihedral results are themselves acknowledged to be unreliable due to missing CVs (lines 252-259). S2-S4 ion occupancies are absent. The Y175-Y175 inter-subunit distance, a classical indicator of SF state in K2P channels, is not shown. Pore hydration is not discussed.

This matters because Figure 6 presents a clean and visually compelling narrative in which up-state equals conductive SF and down-state equals non-conductive SF, and this figure is the central summary of the paper. But it rests almost entirely on ψ-F284, and even there the picture is not as clean as Fig. 6 suggests. In Fig. 4b (apo), a metastable flipped-SF conformation is clearly visible within the up-state region of the FES, at approximately 3 kcal/mol above the minimum. The authors acknowledge this at lines 236-237, but then at lines 417-418 they write that "within these conformations, the SF remains in a stable, conductive configuration as revealed in the crystal structure." These two statements are in direct contradiction and need to be reconciled.

More broadly, the reader is asked to accept that ψ-F284 is a sufficient reporter of conductive versus non-conductive SF states, but no justification for this choice is provided beyond the authors' prior work. Given that the central claim of the paper is an allosteric coupling between M4-pCt dynamics and SF gating, the SF side of this coupling deserves a much more complete characterization. The authors should provide, for each FES minimum identified in Figs. 4 and 5, at minimum the ion occupancy profile across S1-S4, the Y175-Y175 inter-subunit distance distribution, and the pore hydration state, extracted from the reweighted ensemble.

Response: We agree that the previous version of Figure 6 was potentially misleading, and we have therefore changed the label from "*non-conductive*" to "*initial SF conformational changes*". As discussed above, we have now additionally computed the ion occupancy profiles across the S2–S4 binding sites, as well as the inter-subunit Y175–Y175 distance distributions, and included these analyses in the SI. We also discussed that the conformational changes at the S1 binding site correspond to the initial SF rearrangement during the gating process, in the discussion section as follows:" *We noted that the specific SF rearrangements reported in these studies differ. While the MD study by J. T. Brennecke et. al proposed conformational changes at the S3 site, P. Aryal and we independently observed up- and down-state-dependent rearrangements at the S0 and S1 sites. We do not view these results as contradictory, as our previous MD study on non-canonical voltage gating suggested that conformational changes at the S1 and S3 sites in TREK channels are coupled to each other during the gating, while we considered the conformational changes at the extracellular side represents the initial SF gating event. Nonetheless, our OneOPES simulation results were unable to resolve the full SF gating procedure. A posteriori analysis of the reweighted ensembles showed that S2-S4 ion occupancies do not vary substantially across the different simulation conditions (Supplementary Fig. 9). This most likely reflects the fact that these additional SF descriptors were not explicitly biased during the simulations, whereas biasing only the local rearrangements at the S1 binding site is insufficient to drive the full range of SF dynamics at the current simulation timescales*."

Here again, short unbiased MD simulations initiated from the FES minima would be of direct value. If a structure extracted from the conductive FES minimum is placed in an unbiased simulation and the SF remains conductive — meaning ψ-F284 stays canonical, ions occupy the filter, and the pore remains hydrated — this would constitute genuine evidence that the FES minimum corresponds to a functional conductive state. The same test applied to the non-conductive minimum would complete the argument. Without this, the functional interpretation of the FES in terms of ion conduction remains an inference, not a demonstration. The lengths and replicate numbers are very important here, given the SF dynamics in K2P channels, so that 3 times 300-500 ns would be a minimum, without asking the authors to redo everything, which is not my idea.

Response: Similar to the analyses discussed above, we projected our previous unbiased MD trajectories onto the auxiliary CVs of ψ-F284 and ψ-Y175 (Supplementary Fig. 5). The unbiased trajectories populate regions that closely overlap with the minima in the reweighted FESs. The functional interpretation of these FES minima is therefore supported by unbiased simulation data rather than being inferred solely from the biased free-energy landscape.

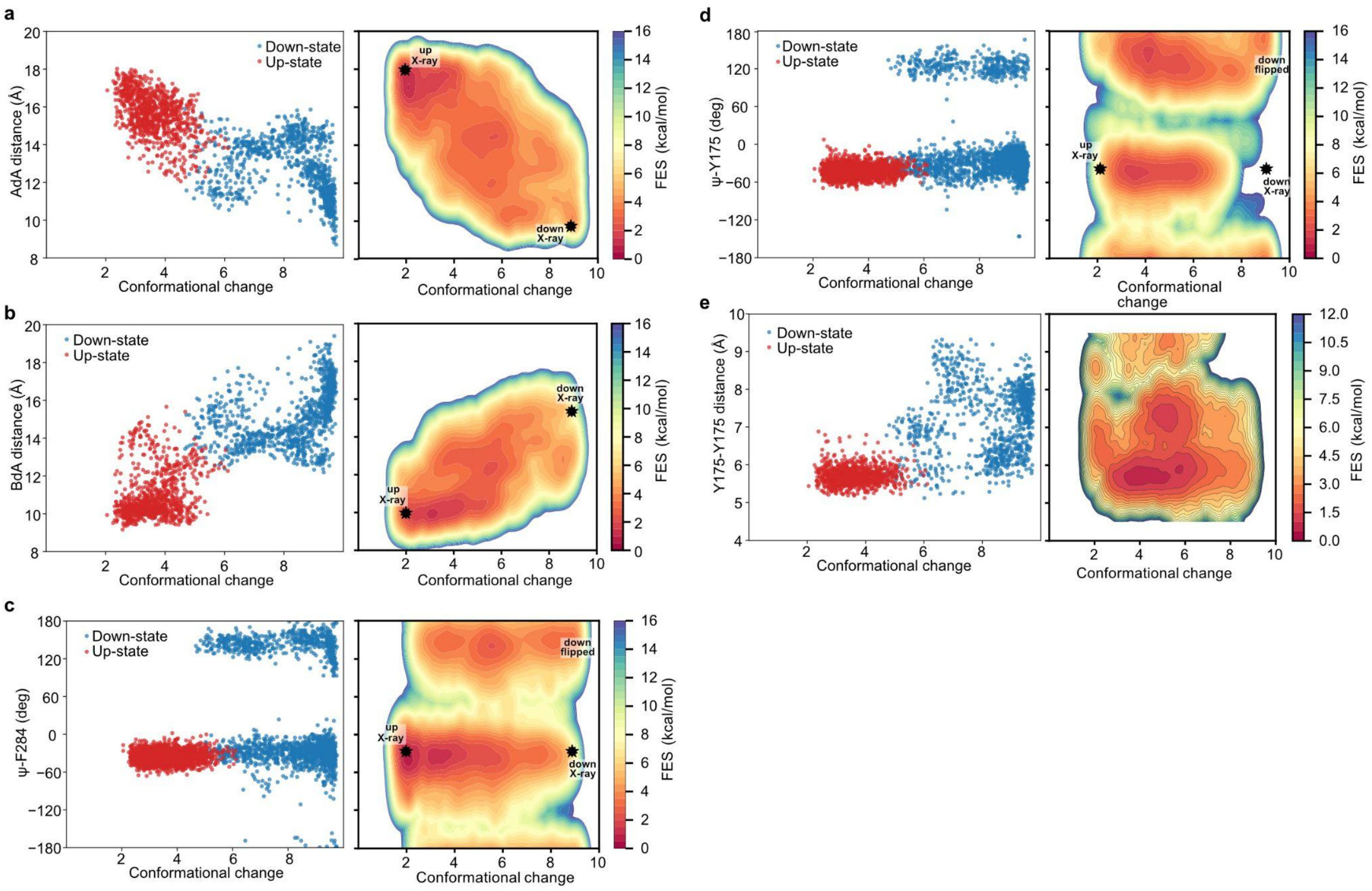

Supplementary Fig. 5. Projection of unbiased up- and down-state TREK-2* simulations onto the OneOPES collective-variable space, shown alongside the corresponding apo OneOPES free-energy surfaces. In each panel, the left plot shows conformations sampled in our previous unbiased MD simulations (ref. 17), five independent 1 µs replicas started from the up-state (red) and five from the down-state (blue), projected onto the collective variables used in the OneOPES setup and plotted against the path CV (s, "conformational change" on the x-axis), which describes the progression from the up-state (low s) to the down-state (high s). The right plot shows the two-dimensional free-energy surface (FES) reconstructed by reweighting the apo TREK-2* OneOPES ensemble (average of three independent replicas) projected along the path CV and the same variable, allowing a direct comparison between the regions spontaneously sampled in the unbiased ensemble and the energetic minima recovered by enhanced sampling. **(a)** Intra-chain M2–M4 distance (AdA, subunit A M2 to subunit A M4). **(b)** Inter-chain M4–M2 distance (BdA, subunit B M4 to subunit A M2). **(c)** Backbone ψ dihedral angle of F284 and **(d)** of Y175, both reporting on the SF conformation at the S1 K^+ binding site. **(e)** Inter-subunit Y175–Y175 backbone carbonyl distance, computed between the centres of mass of the backbone carbonyl atoms (C and O) of Y175 in the two opposing subunits. Black stars indicate the positions of the crystallographic TREK-2 up- and down-state structures mapped onto these coordinates; the "down flipped" label marks the flipped-carbonyl SF conformation accessible in the down-state region. In every panel, the unbiased up- and down-state ensembles partition into distinct regions of the path CV that coincide with the up- and down-state minima of the apo OneOPES FES. Free energies are given in kcal/mol.

VII. The absence of transmembrane voltage affects more than Y175

At lines 429-430, the authors invoke the absence of transmembrane voltage as an explanation for the discrepancy in Y175 sampling between the current study and their prior unbiased simulations. This is reasonable, but the framing is too narrow. Transmembrane voltage affects the global electrostatic environment of the channel — ion occupancy profiles, pore hydration, and potentially the M4-pCt equilibrium itself. By mentioning voltage only in the context of the Y175 discrepancy, the authors implicitly suggest that all other results are unaffected, which is not justified. The limitations section of the discussion should acknowledge voltage as a general limitation of the current setup.

Response: We agree with the reviewer that the absence of transmembrane voltage in our OneOPES setup affects more than the Y175 sampling alone. We have now added the following

section in the Discussion: "*Furthermore, unlike our unbiased simulations performed using the Computational Electrophysiology framework, all free-energy results presented here correspond to the zero-voltage ensemble. While this difference may in addition affect the sampling of Y175 conformational transitions, we acknowledge that absence of transmembrane voltage could, in principle, alter the electrostatic environment of the whole channel and thereby influence channel properties such as K^+ occupancy within the SF, hydration of the inner cavity, and the dynamics of highly charged pCt region. However, incorporating an explicit membrane voltage into a replica-exchange enhanced-sampling framework is methodologically challenging, as it violates the equilibrium conditions required for rigorous bias reweighting. For this reason, the OneOPES simulations were performed here in the absence of an applied voltage, consistent with standard enhanced-sampling protocols that rely on equilibrium sampling and reweighting. Extending these approaches to systematically characterize voltage-dependent modulation of the conformational observables discussed here, for example using voltage-coupled enhanced-sampling schemes, represents an important direction for future methodological and mechanistic studies.*"

VIII. Missing statistical analyses

The 2D FES plots in Supplementary Figs. 2 and 3 are presented purely visually, with no statistical assessment of whether the distributions under different conditions are significantly different from each other. The standard deviation shown in Fig. 2b-d gives some sense of reproducibility, but this is not a test of whether, for example, the apo and phosphorylated landscapes differ significantly. The paper could benefit from quantitative statistical comparisons. Quantitative comparison of the FES minima across conditions — for example, overlap metrics or confidence intervals on the free energy differences between states — could be provided before drawing conclusions about which condition favors which conformation.

Response: We thank the reviewer for raising this point and agree that run-to-run reproducibility and a statistical comparison between conditions are distinct questions. The standard-deviation envelopes in Fig. 2b–d and Fig. 5d address only the former. To quantify the differences between conditions, we have computed the free-energy difference between the up- and down-state basins, ΔG(down–up), for each condition. For each independent run, we integrated the reweighted populations within fixed up- and down-state basins, defined identically across all four systems and anchored to the projected crystallographic up- and down-state positions on the two-dimensional surfaces spanned by the path CV and either the M4–M2 (AdA) distance or the ψ-F284 dihedral. The mean and standard error across independent runs then provide a confidence interval on ΔG(down–up) for each condition, and the difference between any two conditions (ΔΔG) is reported with its propagated uncertainty (new Supplementary Fig. 7 and Supplementary Table 3). The phosphorylated landscape is the only one in which the down-state is favored, and the inter-condition differences are large relative to the run-to-run uncertainty (e.g. apo vs phosphorylated 31.8 ± 3.2 kJ mol^{-1}, ~10σ; stretch vs phosphorylated 54.8 ± 6.5 kJ mol^{-1}, ~8σ). We note that these basin-integrated values reproduce the free-energy differences reported in the main text, now with explicit confidence intervals. We refrained from applying formal significance tests, as the statistical power would remain limited for n = 3 (or 5) independent enhanced-sampling runs. Instead, we consider the magnitude of each ΔΔG relative to its propagated uncertainty to provide a more conservative statement of the robustness of the observed energetic differences. We have added following sentences to the main text:

"To place these condition-dependent shifts on a quantitative footing, rather than relying solely on visual inspection of the FES, we integrated the reweighted populations within fixed up- and down-state basins, defined identically for all systems on the two-dimensional FES spanned by the path CV and either the M4–M2 (AdA) distance or the ψ-F284 dihedral (Details see Methods). From these populations, we calculated the free-energy difference between the two states, ΔG(down–up), for each independent run (Supplementary Fig. 7a), with the same ordering of conformational equilibria obtained for ψ-F284 (Supplementary Fig. 7b). This analysis shows that the phosphorylated system is the only condition in which the down-state is thermodynamically favored, whereas membrane stretch and A314P both shift the equilibrium strongly toward the up-state. The inter-condition free energy differences ΔΔG(down–up) (apo vs phosphorylated: 31.8 ± 3.2 kJ mol^{-1}; apo vs stretch: -23.0 ± 6.1 kJ mol^{-1}) substantially exceed the run-to-run uncertainty, thereby supporting a quantitative distinction between conditions that extends beyond the qualitative visual comparison of the free-energy landscapes (Supplementary Table 3)."

a

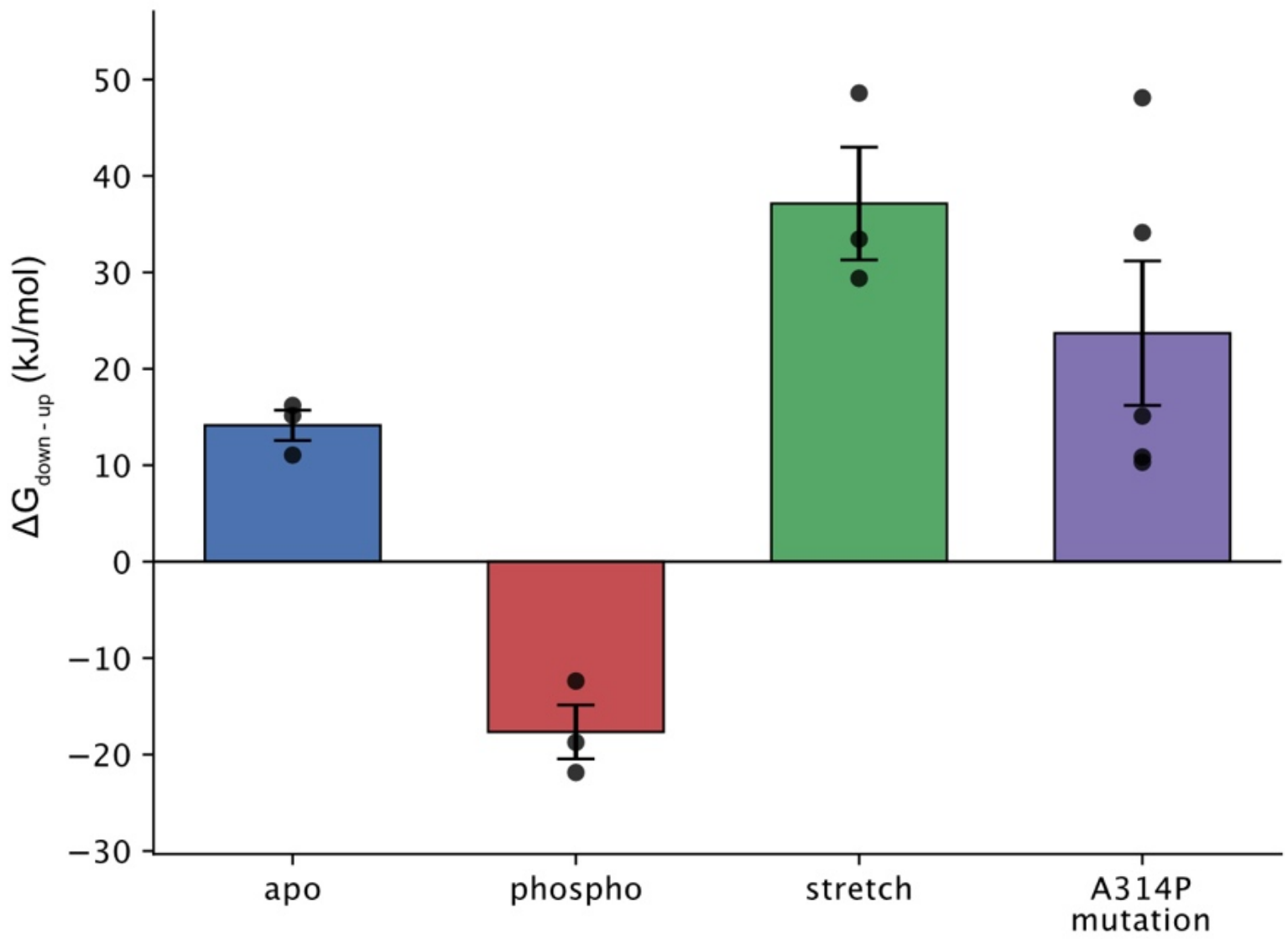

b

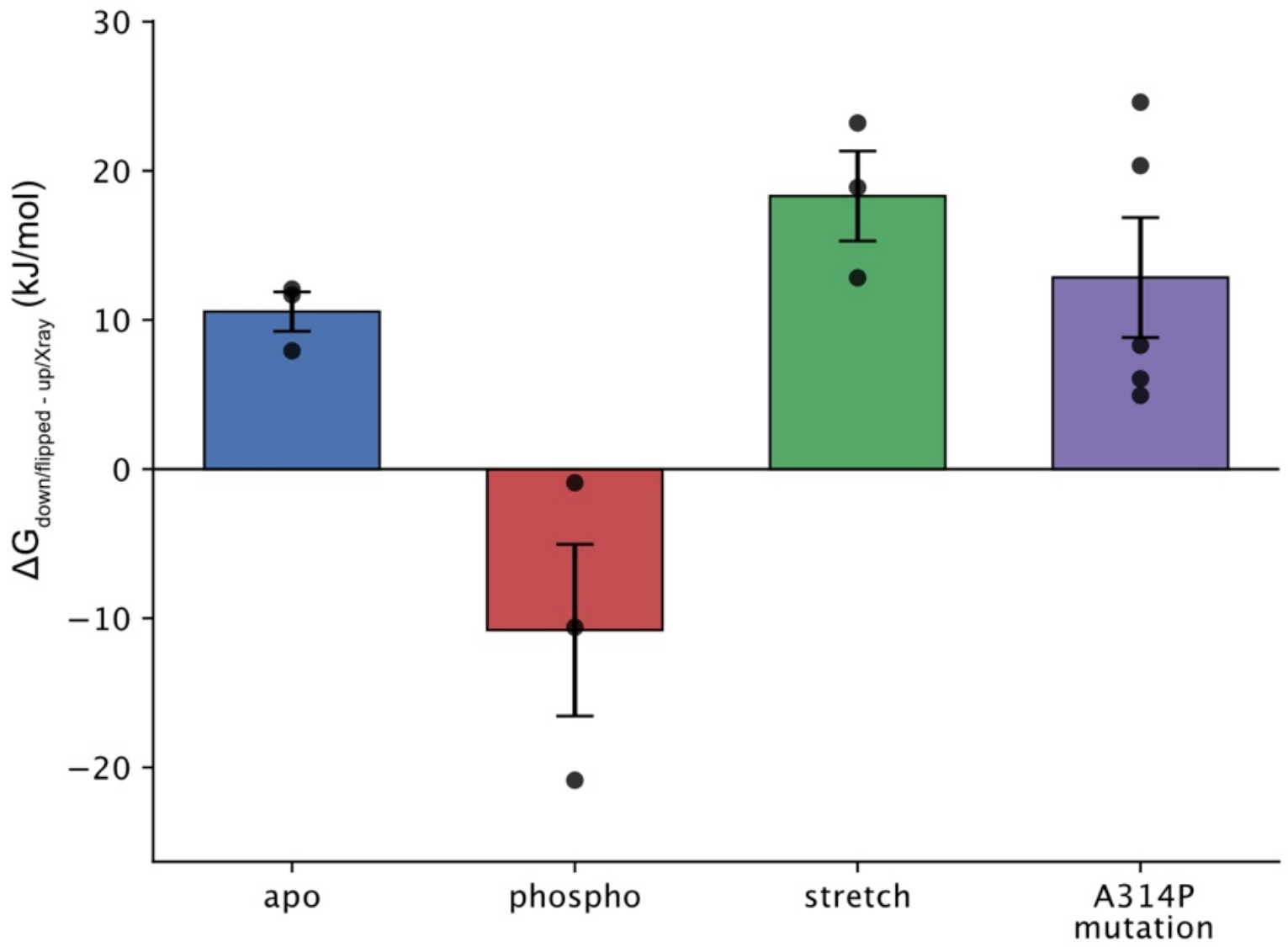

Supplementary Fig. 7. Quantitative comparison of up- and down-state free energies across conditions. Free-energy difference between the down- and up-state basins, for the apo, phosphorylated, stretch-activated and A314P (disease-mimicking) systems, obtained by Boltzmann integration of the reweighted populations within fixed basins defined on the two-dimensional free-energy surfaces. Positive values indicate that the up-state is thermodynamically favored; negative values indicate that the down-state is favored. Full basin definitions and the integration procedure are given in Methods (Basin-integrated free-energy differences). **(a)** ΔΔG calculations were performed by selecting basins from the path CV / M4–M2 (AdA) 2D FES, describing the global up–down conformational change of the M4/pCt module: the up-state basin combines low path-CV values with high AdA, and the down-state basin combines high path CV values with low AdA. **(b)** ΔΔG calculations were performed by selecting basins from the path CV / ψ-F284 2D FES: the up-state basin combines low path-CV values with the X-ray-like ψ-F284 conformation (−60° to −10°), and the down-state basin combines high path-CV values with the flipped ψ-F284 conformation (120° to 180°). Bars are the mean over independent OneOPES runs ($n = 3$ for apo, phosphorylated and stretch; $n = 5$ for A314P), error bars are the standard error of the mean, and black circles show the individual per-run values. Identical basin definitions were applied to all systems.

Supplementary Table 3. Pairwise free-energy comparisons (ΔΔG) between apo and the other conditions. Differences in the basin-integrated free-energy difference ΔG(down–up) between apo and each other condition, evaluated on the two-dimensional reweighted free-energy surfaces along the M4/pCt (AdA) and selectivity-filter (ψ-F284) coordinates. ΔΔG is defined as ΔG(apo) − ΔG(other); a positive value indicates that the up-state is more strongly favored in apo than in the comparison condition, and a negative value the opposite. Uncertainties are propagated from the per-condition standard errors as $\sigma(\Delta\Delta G) = \sqrt{[SE(apo)^2 + SE(other)^2]}$, and the separation is reported as $|\Delta\Delta G|/\sigma$. Per-condition ΔG(down–up) values are means over independent OneOPES runs ($n = 3$ for apo, phosphorylated and stretch-activated; $n = 5$ for A314P). The same ordering of conditions is recovered on both coordinates. All values in kJ mol^{-1}.

comparison	ΔΔG	SE	σ
**M4/pCt (AdA) projection**			
apo − phospho	31.80	3.20	9.9
apo − stretch	−23.00	6.05	3.8
apo − disease	−9.56	7.65	1.2
**SF (ψ-F284) projection**			
apo − phospho	21.35	5.91	3.6
apo − stretch	−7.75	3.29	2.4
apo − disease	−2.29	4.23	0.5

Furthermore, at line 424, the word "correlation" is used to describe a visual observation from 2D FES plots. Correlation has a specific statistical meaning. If the authors wish to claim a correlation between M4-pCt displacement and SF stability, they should quantify it.

Response: We have removed this sentence.

IX. Claims without supporting data

Several statements in the discussion are not supported by any figure or analysis in the paper. Lines 338-343 describe a kink in M4 introduced by the A314P substitution, with a specific orientation that "forces" the pCt-M4 interface toward the opposing M2 helix. The kink is plausible given the general structural effect of proline substitutions, but its orientation and the specific claim about M2 proximity are inferred entirely from the FES minimum position, not from any direct structural analysis. No representative structures from the A314P

trajectory are shown overlaid on WT. No contact distances, kink angles, or helix geometry measurements are provided. The authors should show the structural basis for this claim.

Response: We thank the reviewer for noting this caveat. We have now added a direct structural overlay as a new Supplementary Figure (Supplementary Fig. 8), together with the corresponding geometric measurements in a new Supplementary Table (Supplementary Table 4). A representative structure from the A314P TREK-2* up-state free-energy minimum was superimposed, based on the conserved pore domain, onto the experimentally determined TRAAK A270P structure (PDB 7LJ4) and, for comparison, the down-state TRAAK structure (PDB 4WFF). The overlay shows that the simulated TREK A314P model reproduces the experimental TRAAK A270P conformation: M4 adopts the very similar kinked, up-state geometry, and the two structures superimpose closely, whereas the WT down-state M4 is displaced downward and away from the pore. This agreement is further quantified by two measurements, the local M4 kink angle and the inter-subunit M4–M2 contact distance between L320 (M4) and P198 (M2) of the opposing subunit. A corresponding description has been added to the Results as "*Direct structural comparison provides further insight into the up-state stabilization in the mutant. A representative structure extracted from the A314P up-state minimum shows that the proline substitution introduces a kink in M4 (~66°; Supplementary Fig. 8 and Supplementary Table 4), which laterally displaces the pCt–M4 interface toward the opposing M2 helix and brings L320 (M4) into close contact with P198 (M2) of the neighbouring subunit (3.7–4.1 Å). This geometry is very similar to the experimentally determined TRAAK A270P mutant structure[44] and contrasts with the TRAAK WT down-state, in which the corresponding contact is longer (4.6–7.1 Å).*"

Following addition as made to methods part:
"*A representative structure was extracted from the A314P up-state free-energy minimum. The M4 kink angle was defined as the angle between the vector from the Cα centre of mass of the five residues preceding the substituted position to its Cα, and the vector from that Cα to the Cα centre of mass of the five following residues. The M4–M2 interface distance was the minimum side-chain heavy-atom distance between L320 (M4) and P198 (M2) of the opposing subunit. The same definitions were applied to the TRAAK A270P (PDB 7LJ4) and down-state TRAAK (PDB 4WFF) structures using the equivalent residues (A270, L276, P155), after superposition on the conserved pore domain.*"

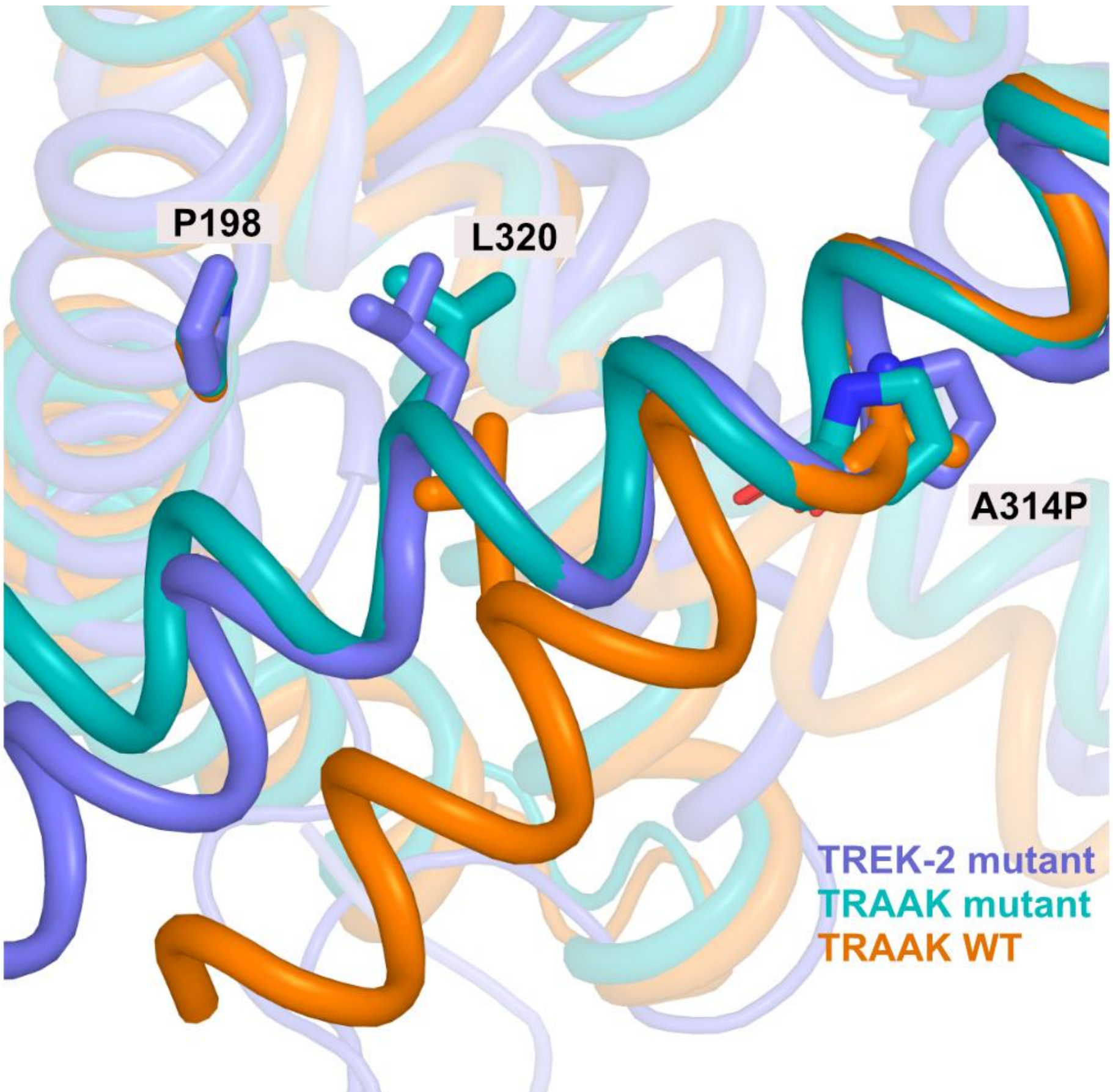

Supplementary Fig. 8. Structural comparison of the A314P-induced M4 kink in TREK-2. Overlay of a representative structure from the A314P TREK-2* up-state free-energy minimum (purple) with the experimentally determined TRAAK A270P structure (teal; PDB: 7LJ4) and the down-state TRAAK structure (orange; PDB: 4WFF). Structures were superimposed on the conserved pore domain so that the position of M4 relative to the rest of the channel is directly comparable; the view is centred on the M4 helix and the intracellular pCt–M4 interface. The substituted residue (A314P in TREK-2*; A270P in TRAAK), the M4 leucine (L320 in TREK-2*; L276 in TRAAK) and the M2 proline of the opposing subunit (P198 in TREK-2*; P155 in TRAAK) are shown as sticks. In both the TREK-2* A314P model and the TRAAK A270P structure, M4 adopts the kinked, upward-displaced up-state conformation and the two structures overlay closely, whereas in the TRAAK WT structure M4 is in the downward (down-state) position. The L320–P198 (L276–P155) side chains are in close contact in the two mutant structures, indicating that the kinked M4 conformation positions the pCt–M4 interface adjacent to the opposing M2 helix.

Supplementary Table 4. M4 kink angle and inter-subunit M4–M2 contact distance for the A314P TREK-2* and reference WT TRAAK structures. Values are reported for a representative structure selected from the A314P TREK-2* up-state free-energy minimum, the experimentally determined TRAAK A270P structure (PDB: 7LJ4), and the down-state TRAAK structure (PDB: 4WFF). The M4 kink angle quantifies the local bend of M4 at the substituted position. The M4-M2 distance corresponds to the minimum side-chain heavy-atom distance between the M4 leucine (L320 in TREK-2*; L276 in TRAAK) and the M2 proline of the opposing subunit (P198 in TREK-2*; P155 in TRAAK).. Both proline-mutated structures (TREK-2* A314P, TRAAK A270P) show a larger M4 kink and a shorter intersubunit M4-M2 contact than the TRAAK WT down-state.

Structure	Kink angle chain A (°)	Kink angle chain B (°)	M4–M2 distance pair 1 (Å)	M4–M2 distance pair 2 (Å)
A314P (model)	67.3	65.4	4.1	3.7

TRAAK A270P (PDB 7LJ4)	62.9	63.7	4.2	3.6
TRAAK WT (PDB 4WFF)	58.7	55.7	7.1	4.6

Lines 431-434 claim that incorporating Y175 as a CV "proved critical for improving the overall description of SF flexibility and for facilitating the proper sampling of F284 rearrangements." There is no figure or control simulation supporting this claim. No comparison with a simulation lacking Y175 as a CV is shown. This statement should be either supported with data or removed.

Response: We have removed this statement.

Lines 411-412 state that "TREK-2 operates through two states of the lower helices corresponding to crystallographic up and down conformations." This is presented as a finding, but it is built into the approach by construction: the path CV was defined between those two states, and the FES will by definition show minima near those endpoints. It would be a genuine finding if the authors had started from an unbiased ensemble and observed spontaneous partitioning into these two states. The framing should be adjusted accordingly.

Response: We agree with the reviewer that the definition of the path CV not only influences the enhanced sampling (as discussed in previous points above) but also to a large extent dictates the location of the metastable states along s. However, we do not agree that a path CV defined between two states will by definition show minima near the endpoints in all systems. This is clear, for example, in the 2D FES (path CV vs AdA, Fig. 3c) of the phosphorylated system, where no metastable state is seen for the up-state. If one of the states is unstable, this will be reflected in the final FES.

From a broader point of view, we acknowledge that, when the CV is constructed from the up and down conformations, observing minima associated with those conformations is not by itself definitive evidence that these are the two fundamental operating states of the channel. This is more an assumption of our setup rather than a finding, informed by previous structural, experimental and computational work. The choice is supported by the following observations from previous unbiased simulations:

- As addressed above, unbiased sampling data for the apo system from previous work shows separation of up and down states in all 2D projections (Supplementary Fig. 5) in good agreement with the corresponding 2D FES, with one trajectory of the less stable down-state venturing towards the up-state basin but seemingly without finding new metastable states.
- For the phosphorylated system, previous unbiased simulations started in the up-state made a full transition to the down-state, indicating that, when the up-state becomes unstable (as seen in the FES), the trajectory moves to the down-state without visiting other large metastable states.

Together, these observations indicate that the two-state behavior is consistent with both independent unbiased and biased simulations and is therefore unlikely to arise solely from the path-CV construction.

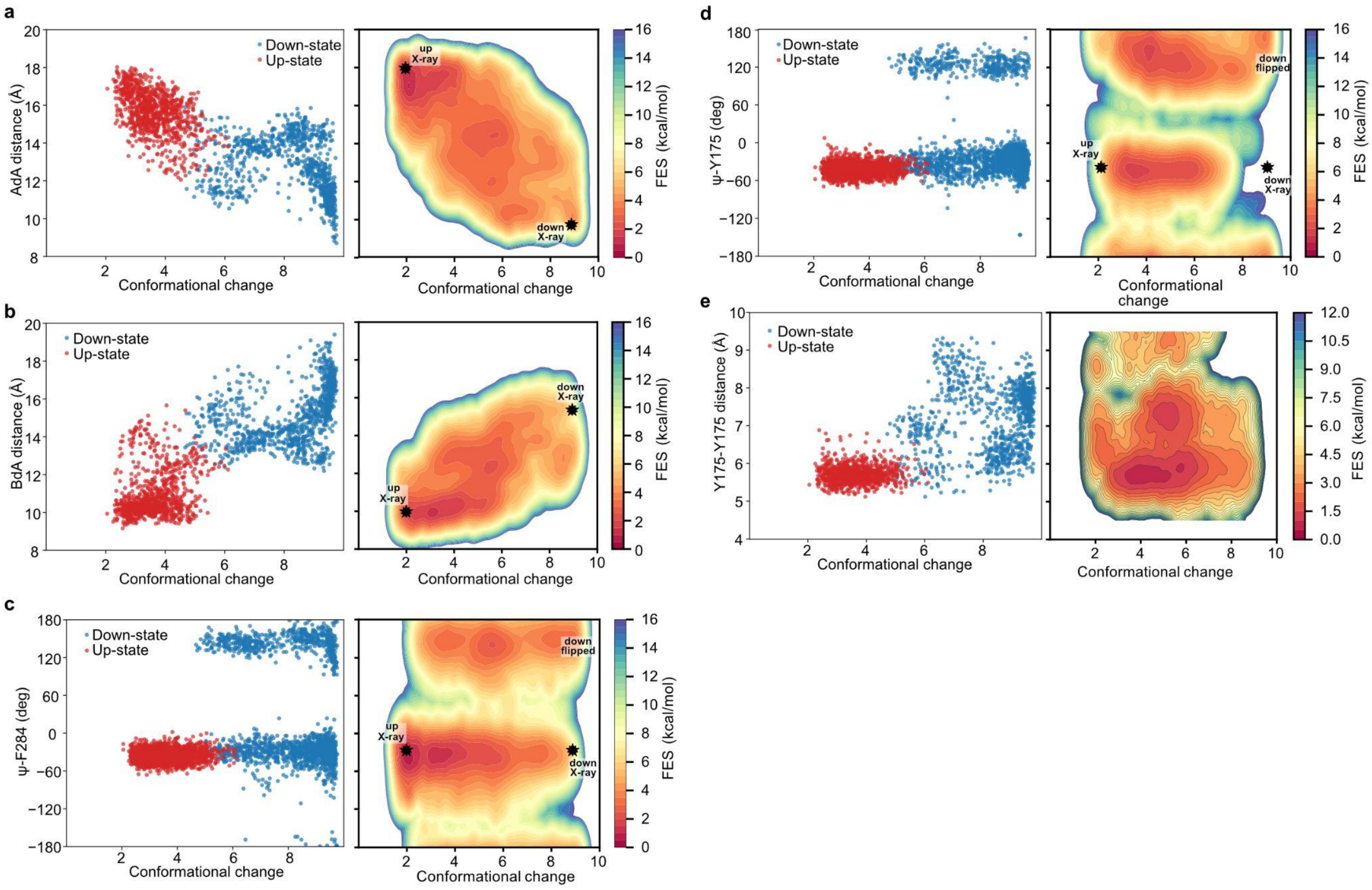

Supplementary Fig. 5. Projection of unbiased up- and down-state TREK-2* simulations onto the OneOPES collective-variable space, shown alongside the corresponding apo OneOPES free-energy surfaces. In each panel, the left plot shows conformations sampled in our previous unbiased MD simulations (ref. 17), five independent 1 μs replicas started from the up-state (red) and five from the down-state (blue), projected onto the collective variables used in the OneOPES setup and plotted against the path CV (s, "conformational change" on the x-axis), which describes the progression from the up-state (low s) to the down-state (high s). The right plot shows the two-dimensional free-energy surface (FES) reconstructed by reweighting the apo TREK-2* OneOPES ensemble (average of three independent replicas) projected along the path CV and the same variable, allowing a direct comparison between the regions spontaneously sampled in the unbiased ensemble and the energetic minima recovered by enhanced sampling. **(a)** Intra-chain M2–M4 distance (AdA, subunit A M2 to subunit A M4). **(b)** Inter-chain M4–M2 distance (BdA, subunit B M4 to subunit A M2). **(c)** Backbone ψ dihedral angle of F284 and **(d)** of Y175, both reporting on the SF conformation at the S1 K^+ binding site. **(e)** Inter-subunit Y175–Y175 backbone carbonyl distance, computed between the centres of mass of the backbone carbonyl atoms (C and O) of Y175 in the two opposing subunits. Black stars indicate the positions of the crystallographic TREK-2 up- and down-state structures mapped onto these coordinates; the "down flipped" label marks the flipped-carbonyl SF conformation accessible in the down-state region. In every panel, the unbiased up- and down-state ensembles partition into distinct regions of the path CV that coincide with the up- and down-state minima of the apo OneOPES FES. Free energies are given in kcal/mol.

About the abstract

The abstract makes three claims that deserve scrutiny in light of the detailed problems identified in the full manuscript.

The phrase "detailed energetic characterization of the TREK-2 conformational landscape" overstates what is achieved. The conformational landscape explored is, by construction, the one-dimensional axis between two known crystal structures, defined by a path CV built from their RMSD difference. A landscape explored along a predefined coordinate between two known endpoints is not the same as a general characterization of the conformational space available to the channel. An unknown third basin, or a conformation not present in either crystal structure, would not be detected by this approach. The word "detailed" should be replaced by something more accurate, such as "energetic characterization of the up-to-down conformational transition."

The phrase "our simulations uncover a unifying mechanism in which coupling between the lower transmembrane helices, the proximal C-terminal domain, and selectivity filter stability governs channel gating" is the most problematic sentence in the abstract, and arguably in the entire manuscript. As detailed in sections V and VI of this review, what the simulations show is a thermodynamic co-variation between M4-pCt position and SF dihedral state within a biased FES. This is not a mechanism. A mechanism requires demonstration of a causal pathway — which residues communicate the signal, in which order, through which interactions. None of this is provided. Furthermore, the SF characterization rests almost entirely on a single dihedral angle (ψ-F284), which is an insufficient proxy for the functional state of a K2P selectivity filter. The word "mechanism" should be removed from the abstract and replaced with "thermodynamic association" or "energetic coupling," pending the additional analyses requested in the full review.

The final sentence, claiming that the results "provide a foundation for structure-based drug discovery," is unsupported by anything in the manuscript. No ligand binding, no druggable site identification, no pharmacophore analysis is presented. The NFx experiment validates a conformational shift but is not a drug discovery study. This sentence should be removed or substantially toned down.

The one sentence that is well-supported is the description of the electrophysiological validation using NFx — this is the strongest part of the paper and accurately described in the abstract.

Response: We thank the reviewer for these suggestions and have revised the abstract as the following: "*The TREK subfamily of two-pore domain potassium (K2P) channels are essential regulators of membrane excitability, and their activity is modulated by a wide range of physiological stimuli, including phosphorylation and membrane stretch. Single-site mutations in this subfamily have been identified in patients with FHEIG symptom (facial dysmorphism, hypertrichosis, epilepsy, intellectual disability/developmental delay, and gingival overgrowth), where they cause pathological channel hyperactivation. Using OneOPES framework, which unifies multiple enhanced-sampling molecular dynamics strategies, we provide an energetic characterization of the up-to-down conformational transition of TREK-2 under physiological stimuli and disease-mimicking conditions. These simulations reveal a shared energetic axis along which coupling between the lower transmembrane helices, the proximal C-terminal domain, and the selectivity filter governs channel gating. The predicted conformational effects of an FHEIG syndrome-associated mutation were further validated by electrophysiological measurements using a conformation-sensitive TREK-2 inhibitor. Together, these results highlight both the power and the methodological challenges of advanced enhanced-sampling approaches for resolving the complex conformational landscapes of ion channels. At the same time, they establish an energetic framework for TREK-2 regulation and dysfunction, together with our previous unbiased MD simulations, complements prior structural and mechanistic studies of this channel family.*"

Summary

The biological question addressed in this manuscript is important, and the electrophysiological validation of the A314P mutant using norfluoxetine is genuinely informative and constitutes the strongest contribution of the work.

However, I have concerns about the computational part of the study that I believe need to be addressed before the manuscript can be considered for publication. The claims made in the abstract — a mechanistic characterization of allosteric coupling, a detailed exploration of the conformational landscape — are not fully supported by the data presented. What is actually delivered is an energetic description of the transition between two known crystal structures along a predefined coordinate, with thermodynamic associations between M4-pCt position and SF dihedral state that are, at least in part, geometrically implied by the way the path collective variable was constructed. This is a useful contribution and worth publishing, but it is not the same as a mechanism, and it is not a landscape exploration in any general sense. The allosteric coupling between M4-pCt dynamics and SF gating — presented as the central mechanistic finding — is inferred from co-variation of observables within a biased simulation, without causal analysis. The SF characterization rests almost entirely on a single dihedral angle, which is an insufficient proxy for the functional state of a K2P filter. Several statements in the discussion refer to structural features or mechanistic pathways for which no supporting figure or analysis is provided. A small number of factual errors and figure cross-reference mistakes also need to be corrected.
The path to publication is clear, but it requires additional analyses and a recalibration of the language throughout, starting from the abstract, to bring the claims into closer correspondence with what the data show. I detail what is needed in the sections above and recommend major revision accordingly

Reviewer #2 (Remarks to the Author)

The manuscript by Turkaydin et al. used MD simulation to understand the energetic landscape of the effects of S331 phosphorylation and a disease-causing gain-of-function mutation on the conformational landscape of TREK2. The work also highlights key experimental evidence supporting the findings. Although the findings on the effects of phosphorylation and mutation are not particularly novel, the quality of the computational work, the application of the method (OneOPES), and the rigour of the methodology prove that the work deserves its place in Nature Communications. This work has proven that rigorous computational work will push the ion channel community forward. Though revision is needed on the following points to strengthen the manuscript.

Major

Given that the finding on the effect of the mutation on the energetic landscape is predictable, I look forward to see how simulations can provide mechanistic insight. Could you explain, or propose why and how the phosphorylation and the mutation structurally affect the up-down conformation distribution?

Response: We thank the reviewer for raising this important point and acknowledge that we failed to make this aspect clear enough in the previous version of the manuscript. We have now performed a more detailed structural comparison of the TREK-2 A314P mutant with both the previously determined corresponding mutant structure of the TRAAK channel and the WT channel structure. This comparison is presented in the revised manuscript as Supplementary Figure. 8 and Supplementary Table 4. These analyses revealed that the A314P substitution introduces a kink in M4, which brings pCt/M4 helices in closer proximity to the opposing M2

helix. This effect contrasts with the effect of phosphorylation described in our previous work (PMID: 38821927), where phosphorylation disrupted the hydrophobic interaction between S331 in the pCt and F202 in the M2 helix of the opposing subunit. We have now incorporated and clarified this mechanistic comparison in the revised manuscript: "*Direct structural comparison provides further insight into the up-state stabilization in the mutant. A representative structure extracted from the A314P up-state minimum shows that the proline substitution introduces a kink in M4 (~66°; Supplementary Fig. 8 and Supplementary Table 4), which laterally displaces the pCt–M4 interface toward the opposing M2 helix and brings L320 (M4) into close contact with P198 (M2) of the neighbouring subunit (3.7–4.1 Å). This geometry is very similar to the experimentally determined TRAAK A270P mutant structure[44] and contrasts with the TRAAK WT down-state, in which the corresponding contact is longer (4.6–7.1 Å). Notably, this structural effect is opposite to the mechanism we previously proposed for phosphorylation-induced inhibition, in which phosphorylation at S331 disrupts the intersubunit hydrophobic interactions between S331 and F202 at M2.*"

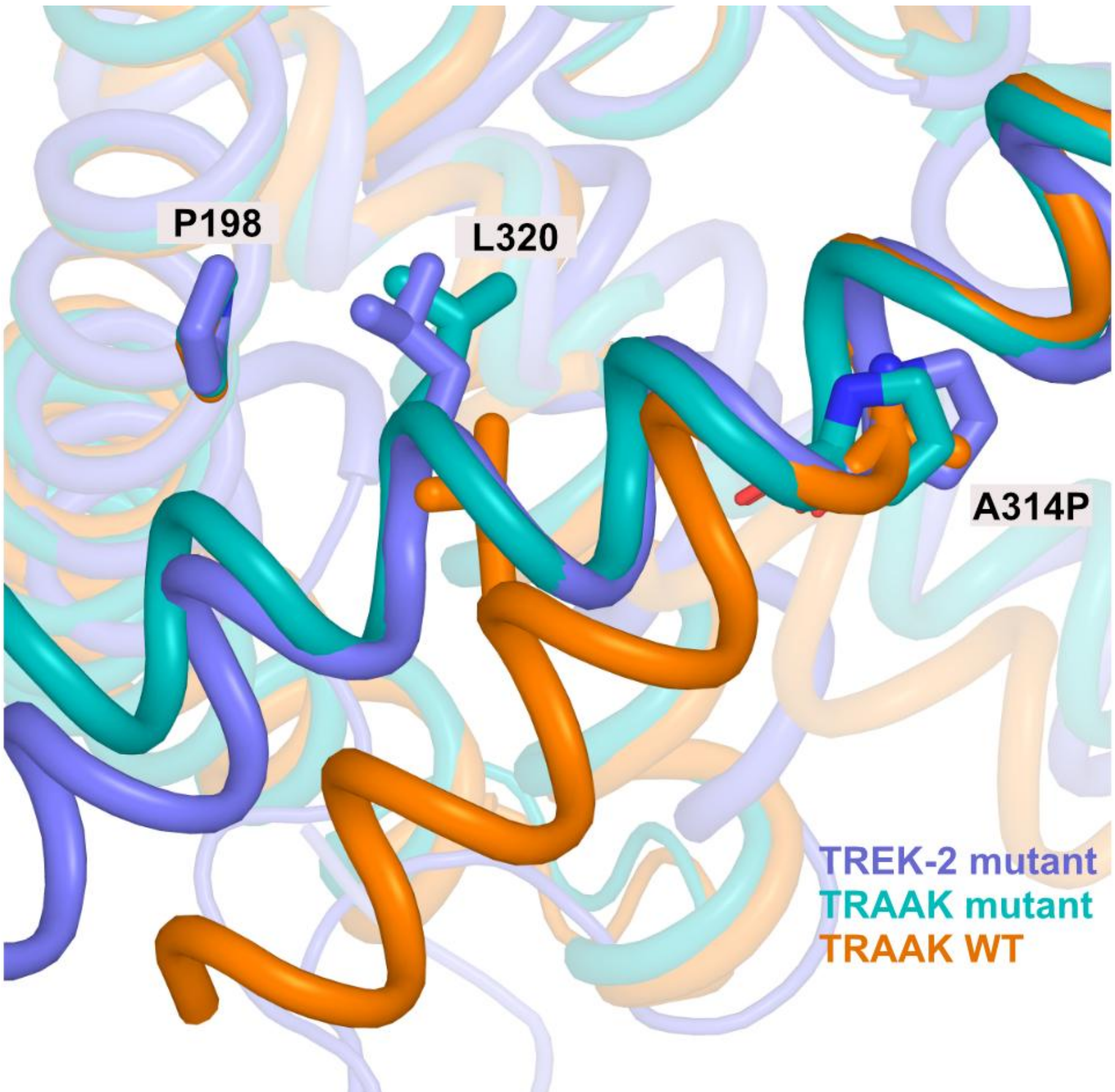

Supplementary Fig. 8. Structural comparison of the A314P-induced M4 kink in TREK-2. Overlay of a representative structure from the A314P TREK-2* up-state free-energy minimum (purple) with the experimentally determined TRAAK A270P structure (teal; PDB: 7LJ4) and the down-state TRAAK structure (orange; PDB: 4WFF). Structures were superimposed on the conserved pore domain so that the position of M4 relative to the rest of the channel is directly comparable; the view is centred on the M4 helix and the intracellular pCt–M4 interface. The substituted residue (A314P in TREK-2*; A270P in TRAAK), the M4 leucine (L320 in TREK-2*; L276 in TRAAK) and the M2 proline of the opposing subunit (P198 in TREK-2*; P155 in TRAAK) are shown as sticks. In both the TREK-2* A314P model and the TRAAK A270P structure, M4 adopts the kinked, upward-displaced up-state conformation and the two structures overlay closely, whereas in the TRAAK WT structure M4 is in the downward (down-state) position. The L320–P198 (L276–P155) side chains are in close contact in the two mutant structures, indicating that the kinked M4 conformation positions the pCt–M4 interface adjacent to the opposing M2 helix.

Supplementary Table 4. M4 kink angle and inter-subunit M4–M2 contact distance for the A314P TREK-2* and reference WT TRAAK structures. Values are reported for a representative structure selected from the A314P TREK-2* up-state free-energy minimum, the experimentally determined TRAAK A270P structure (PDB: 7LJ4), and the down-state TRAAK structure (PDB: 4WFF). The M4 kink angle quantifies the local bend of M4 at the substituted position. The M4-M2 distance corresponds to the minimum side-chain heavy-atom distance between the M4 leucine (L320 in TREK-2*; L276 in TRAAK) and the M2 proline of the opposing subunit (P198 in TREK-2*; P155 in TRAAK).. Both proline-mutated structures (TREK-2* A314P, TRAAK A270P) show a larger M4 kink and a shorter intersubunit M4-M2 contact than the TRAAK WT down-state.

Structure	Kink angle chain A (°)	Kink angle chain B (°)	M4–M2 distance pair 1 (Å)	M4–M2 distance pair 2 (Å)
A314P (model)	67.3	65.4	4.1	3.7
TRAAK A270P (PDB 7LJ4)	62.9	63.7	4.2	3.6
TRAAK WT (PDB 4WFF)	58.7	55.7	7.1	4.6

The standard deviation between 3 repeats of OneOPES is quite large, given that each run has its own minima. Can you explain why that is happening and discuss it further in the discussion?

Response: We thank the reviewer for this important observation. The relatively large STD between independent OneOPES repeats arises from a combination of methodological and physical factors intrinsic to enhanced sampling of high-dimensional free-energy landscapes.

First, the OneOPES framework employed here integrates replica-exchange sampling with multiple biased ensembles, allowing configurations to be exchanged across replicas that explore different regions of phase space. While this significantly enhances sampling efficiency, it also promotes exploration of multiple competing minima within each independent run. As a result, different repeats may transiently favor slightly different regions of the conformational landscape, contributing to variability in the reconstructed free-energy surfaces. Second, the observed variability reflects the inherent complexity of the system and the choice of CVs. Although we believe that our CV set is able to largely capture the dominant slow degrees of freedom (path CV, M4-M2 displacements, and SF descriptors), no low-dimensional CV representation can perfectly describe all orthogonal degrees of freedom in a system of this size and complexity. Residual non-accelerated degrees of freedom can therefore lead to differences in how individual runs explore metastable states, which is a well-known source of variance in enhanced sampling simulations. As always in enhanced sampling, a more refined CV set would eventually bring the error bars down, but such a set is far from trivial to figure out and often comes at the price of building an ad-hoc pipeline. We think that, given the complexity of our system, our CV set and the OneOPES strategy strike a good balance between CV complexity and intuitiveness. Third, we performed three fully independent OneOPES simulations, each consisting of multiple replicas. This strategy provides a more rigorous estimate of the intrinsic variability and statistical uncertainty of the calculated free-energy landscapes. In finite-length enhanced sampling simulations, the quasi-equilibrium

regime used for free-energy estimation inevitably retains some memory of the preceding non-equilibrium exploration and bias construction. Independent simulations therefore provide a more reliable framework for error estimation, as each simulation undergoes an independent exploration and bias-generation process rather than sharing the same non-equilibrium history. In the enhanced sampling field, convergence is often assessed primarily from a single simulation or from internally correlated replicas, which can underestimate the true uncertainty of the calculated free-energy surface. In contrast, we explicitly evaluated reproducibility across fully independent simulations. Although the standard deviations across the three independent simulations remain substantial, we believe it is important to report these values transparently, as they reflect the genuine difficulty of achieving full convergence in large, complex membrane-protein systems with rugged conformational landscapes.

To make this point more clear, we have expanded the following Discussion section to explicitly address the origin of this variability and to emphasize that: (i) variability is expected for replica-exchange enhanced sampling in complex systems and (ii) it is amplified by independent repeats and imperfect CV representations: "*A practical aspect of the OneOPES results worth commenting on is the run-to-run variability of the reconstructed free-energy profiles, visible as the standard-deviation envelopes in Fig. 2b–d and Fig. 5d. Some level of variability is expected for replica-exchange enhanced sampling of high-dimensional systems: each independent run explores a slightly different sequence of replica exchanges and accumulates a slightly different bias history, and any low-dimensional CV set, however carefully chosen, leaves residual hidden degrees of freedom that contribute additional dispersion. Reporting variance across three (or, for the A314P system, five) independent runs is therefore a more conservative estimate of reproducibility than block analysis of a single trajectory, but it also produces a larger apparent standard deviation.*"

I also find the convergence analysis in this manuscript (Figure 2e) quite difficult to interpret. Is the idea simply that all conformation (ranging from 2 to 9) is being sampled?
If that is the case, is the colour bar describing the system's bias to leave the minimum? The range of the colourbar doesn't seem to be covered - why did we have to go all the way to -50?

Response: The primary purpose of Fig. 2e is indeed to demonstrate that the system efficiently samples the full conformational space along the path CV, spanning the range between the up- and down-state. The figure shows that transitions across this coordinate occur multiple times during the simulation, indicating good exploration of the relevant configurational space.

In OPES, the bias is constructed from an on-the-fly estimate of the probability density along the CV, and is designed to enforce a chosen target distribution. More concretely, the bias corresponds to a logarithmic reweighting (hence can be positive and negative) of the sampled distribution (from KDE) towards the target distribution (well-tempered, i.e. shallower), effectively counteracting the underlying true free energy surface and promoting exploration. Importantly, the relatively wide range of the color scale (from ~−50 kJ/mol to ~50 kJ/mol) originates from the initial stages of the simulation, during which OPES operates in an exploratory regime. In this phase, the probability density estimate is still inaccurate and rapidly evolving. This transiently results in large positive and negative bias values that help the system quickly access previously unexplored regions of the CV. All of this is expected for OPES and OneOPES simulations.

As the simulation progresses and the probability density estimates improve, the bias stabilizes and eventually reaches quasi-equilibrium becoming effectively time-independent, corresponding to a well-tempered compensation of the underlying free energy surface and yielding stationary sampling consistent with the target distribution. The final free energy surfaces are obtained through proper reweighting where the initial 100 ns were skipped, and thus are not affected by these early large out-of-equilibrium bias fluctuations.

To improve clarity, we have revised both the legends of Fig. 2e and the corresponding Results section to explicitly state that (i) the figure demonstrates full sampling along the path CV, (ii) the color scale represents the OPES Explore bias potential applied along the path CV at each frame, and (iii) the large bias amplitudes observed at early simulation times correspond to the initial exploration and bias-construction phase of the OPES algorithm, rather than to the converged sampling regime. We added the following description to the figure legends and some discussions in the main text: "*Fig2 (e) Sampling of the path CV as a function of time in replica 0 of an apo TREK-2 OneOPES simulation. Data points are colored according to the accumulated OPES Explore bias potential applied along the path CV at each frame. The broad bias range observed during the early phase of the trajectory (~|50| kJ/mol) reflects the initial exploratory regime of OPES, during which the on-the-fly kernel density estimation (KDE) of the probability distribution is still rapidly evolving. The bias stabilizes as the simulation progresses (Supplementary Fig. 3), and the final FES is obtained by reweighting after discarding the first 100 ns of each replica.*"

"*Fig. 2e shows that replica 0 repeatedly traverses the full range of the path CV, demonstrating efficient sampling of the up- and down-state basins, as well as the intermediate conformational region. The color scale represents the accumulated OPES Explore bias applied along the path CV at each frame. Large positive and negative bias excursions are observed during the early phase of the trajectory and reflect the rapidly evolving probability-density estimate characteristic of the initial exploratory phase of OPES. As the simulation progresses, the bias potential progressively stabilizes (Supplementary Fig. 3). The final FES is obtained by reweighting after discarding the first 100 ns of each replica, thereby preventing early-stage bias fluctuations from contributing to the converged free-energy profiles*."

My main question will be how movement up and down the allosteric pathway communicates to the selectivity filter. Given that you managed to sample the basin in both states, can you explore whether there is any allosteric communication between the Ctd to the SF. You also have cases where the mutation and phosphorylation show opposite effects, so it should be possible to describe the rearrangement.

Response: We thank the reviewer for raising this important point and acknowledge that we failed to make this aspect clear enough in the previous version of the manuscript. In our earlier work (PMID: 38821927), unbiased MD simulations combined with network analysis suggested two potential allosteric pathways coupling of the pCt/M4 region to the SF. These proposed pathways were also consistent with previous mutagenesis studies discussed in that work. However, an important limitation of the previous study was that these conclusions were based mainly on unbiased MD simulations, in which only a few spontaneous transitions between the up- and down-states were observed. Consequently, the sampling was insufficient to quantitatively characterize the underlying free-energy landscape or to determine how external stimuli shift the conformational equilibrium.

We previously attempted to address this issue using several enhanced sampling approaches, but were unable to achieve satisfactory sampling convergence for this large-scale conformational transition. This limitation became a major motivation for the current study. Here, we applied the more advanced OneOPES enhanced sampling framework to quantitatively characterize the free-energy surfaces associated with the up/down transition and SF dynamics, and to investigate how phosphorylation, membrane stretch, and disease-associated mutations reshape this energetic landscape. In this way, the present work provides a more quantitative and robust assessment of the allosteric coupling mechanisms proposed in our earlier study.

To make these points clear, we have included the following text at the beginning of the Discussion section: "*In our previous work, we showed that the crystallographic up- and down-state conformations of TREK-2 channel display markedly different ion conductivities in the simulations. These differences were associated with persistent conformational rearrangements at the extracellular side of the SF, particularly involving residues that stabilize the S1 ion binding site. Furthermore, network analysis of the MD trajectories, based on pairwise residue interactions that differed between two states, suggested two potential allosteric coupling pathways linking the pCt/M4 region to the SF: (i) M4 → M4 loop/EC loop → SF; (ii) M4/M2 → P2 → SF. However, as only a few spontaneous transitions between the up- and down-states were observed in these unbiased simulations, the sampling was insufficient to quantitatively characterize the conformational equilibrium or determine how external stimuli quantitatively reshape the underlying free energy landscape.*
In the present work, we revisited this problem using the enhanced sampling framework OneOPES to calculate the FESs associated with conformational changes in both the pCt/M4 region and the upper part of the SF. "

I don't think the effect of the A314P mutation on the dihedral around F284 is that pronounced, as it is very close to the edge. Can you run longer, or quantitatively compare the depth of the energy basin between them to make the point clearer?

We agree with the reviewer that the effect of the A314P mutation on the stabilization effect of the conductive F284 conformation is less pronounced than the effects of membrane stretch or the opposing destabilization effect induced by phosphorylation. For this mutant, we have already increased the number of independent simulation runs from three to five. Despite the additional sampling, we observed substantial variation of the relative population of the SF conformations across independent simulation replicas This suggests that the effect of the mutation on the free-energy landscape is comparatively subtle. We therefore think that much longer simulations will be required to achieve full convergence and to quantify the energetic consequences of the mutation with greater confidence. We have now included this discussion in the main text as: "*We next examined whether this stabilization of the up-state affected the energetics of the SF. Projections of the FESs along the path CV and the auxiliary CV describing the ψ dihedral angle of F284 revealed, on average, a comparable population distribution between the X-ray-like and backbone-flipped conformations in the A314P mutant and WT channel (Fig. 5f, Supplementary Fig. 7). However, we observe substantially larger variation in the relative populations across independent simulation replicas in the mutant system, indicating reduced convergence of the free-energy estimates (Supplementary Fig. 7). This*

variability limits our ability to draw conclusions regarding the precise energetic impact of the mutation of the equilibrium between the two SF conformations."

Minor

Please fix kj/mol to kJ/mol with capital J for the unit of energy throughout the manuscript.

Response: This has been corrected now throughout the manuscript

If stated in the figure legend, such as Fig. 2, the standard deviation across 3 runs should be reported. Otherwise, could it be confused with the standard deviation due to data binning? I think you can put Suppl. 2 together with Fig. 3 and Suppl. 3 with Fig. 4.

Response: Explanation added to Figure captions: The solid line represents the mean of three independent simulations. The shaded region corresponds to the standard deviation computed across the three kernel-reweighted free energy profiles at each point. This reflects run-to-run variability and does not include statistical uncertainty from binning.

Figure 5D has no replica, and STD is shown. Maybe there is a version error? Can you show all the data, please?

Response: There was an error during the export of the Word file to PDF format. This issue has now been corrected, and the updated figure includes both the replica data and the corresponding STD values.

Figures 5e/f have overlapping labels; that needs to be fixed.

Response: Labels have been corrected now.

Line 354 - Figure 5, what? Seems incomplete.

Response: We have corrected this mistake and indicated now the corresponding Figure part (Fig. 5e,f).

Line 152 - When compared to the experimental data, can you always justify whether it compares well quantitatively (just the trend), or quantitatively, where the IC50 matches the free energy of the basin?

Response: As a back-of-the-envelope cross-check, the A314P/WT NFx IC_{50} ratio can be converted into an approximate conformational free energy shift via $\Delta\Delta G \approx RT\ln(\mathrm{IC}_{50}^{apo}/\mathrm{IC}_{50}^{mut}) = RT\ln(3.4/256.7) \approx -10.8$ kJ/mol, favoring the up-state in the mutant. This relies on a two-state model in which NFx binds exclusively to a down-state with unchanged intrinsic affinity in the mutant, and is valid in the limit where the up-state is strongly dominant in both WT and A314P (justified here, since apo simulations show the up-state already favored by ~3 kcal/mol, i.e. $P_{up}^{WT} \approx 0.997$, and mutant even higher). This estimate is in good agreement with the independent OneOPES free energy estimate of ΔΔG=-9.6±7.7 kJ/mol (apo vs disease-mimic simulations, Supplementary Table 3). Nevertheless, we decided not to include it in the

current version as we did not feel confident a single mutation is sufficient to validate the model. We have revised one sentence in the introduction: "*Our predictions of how GoF mutations in TREK-2 homologue to the pathogenic FEIGH syndrome mutations in TRAAK alter the energetic landscape were further qualitatively supported by state-dependent norfluoxetine (NFx) inhibition experiments via inside-out patch-clamp measurements.*

Reviewer #3 (Remarks to the Author):

The authors use enhanced-sampling molecular dynamics to study the energetic landscape of TREK-2 channels during phosphorylation, stretch and in response to mutations. The energy landscapes are consistent with experimental data, which makes the model a useful tool. But the authors are not pushing the limits of their simulations, in my mind. Some more unexpected findings or novel mechanisms would enhance this manuscript.

Major comments

1. The authors use a morph to find intermediate in their enhanced sampling strategy. How can you know if this CV path is correct or the only one? They use some extra CVs, but are these sampling enough of the conformational space?

Response: We thank the reviewer for raising this important point. The distance-from-path variable z was monitored throughout all OneOPES simulations and is now reported in a new Supplementary Figure (Supplementary Fig. 2). Across all systems and replicas, the marginal distribution of z is sharply peaked, with the 95th percentile remaining below ~0.2 nm^2 (mean squared displacement units), demonstrating that the sampled conformations remain tightly confined to the interpolated tube defined by the ten morph structures. Within this regime, the instantaneous values of s can be safely interpreted as well-defined progress coordinates along the path, rather than ambiguous projections onto a poorly resolved transition tube. We have additionally included both the time evolution and the one-dimensional distribution of z for all systems in Supplementary Figure 2. Convergence analysis of the OneOPES bias potential and the time evolution of the path CV, further supports robust and reproducible sampling across all replicas (Fig. 2e and Supplementary Fig. 1, 2). Importantly, projection of our unbiased apo trajectories onto different CVs (now shown in Supplementary Fig. 5) revealed no conformational regions that were sampled outside the configurational space explored by the OneOPES simulations. These results collectively support the adequacy of the chosen CVs and the stability of the sampled transition ensembles.

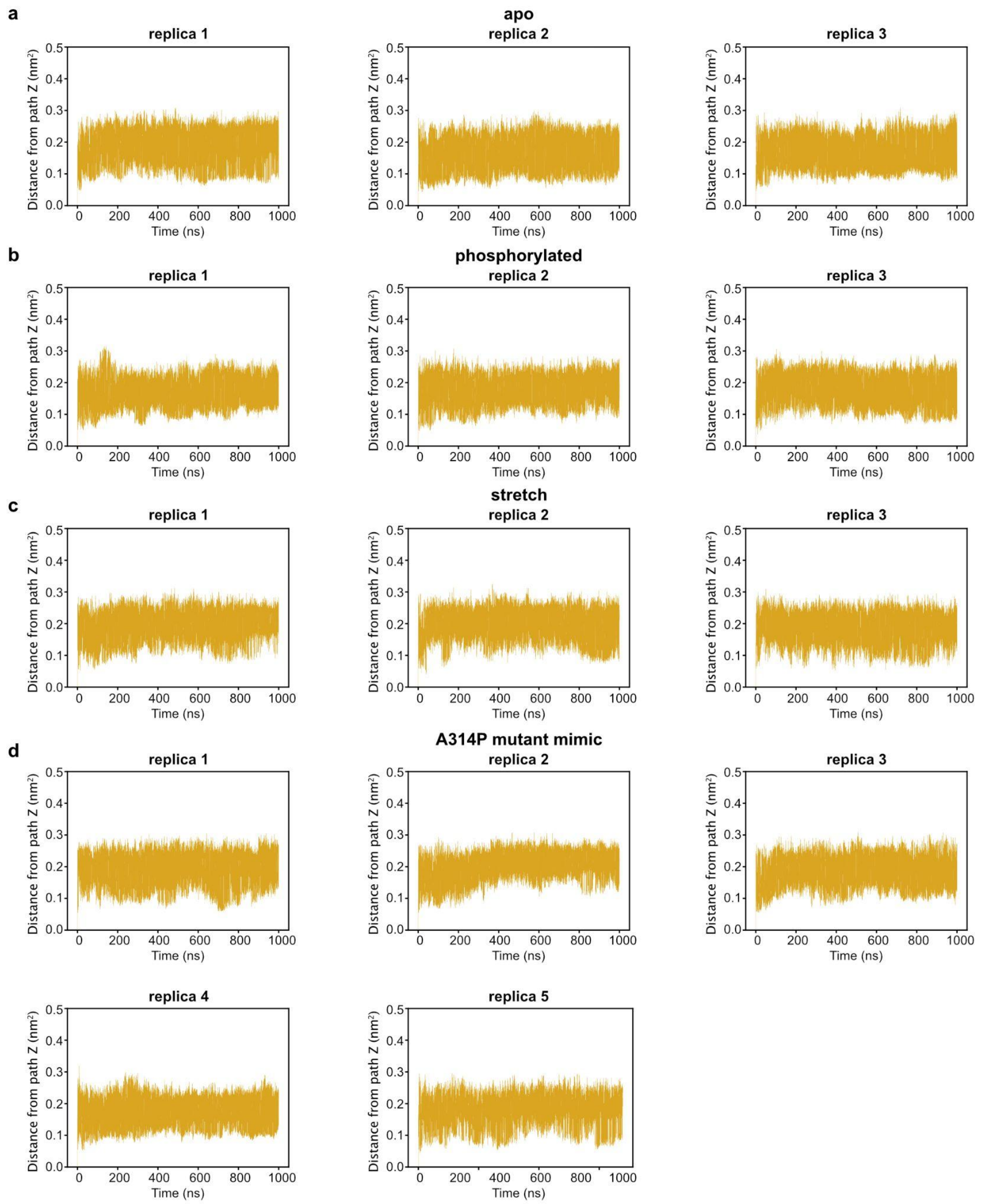

Supplementary Fig. 2. Time evolution of the distance-from-path variable Z. Timewise plots for all replicas across the four systems: **(a)** apo, **(b)** phosphorylated**, (c)** membrane-stretch, and **(d)** disease-mimic mutant. The variable Z represents the RMSD distance from the predefined path collective variable S.

2. Line 248-249. "providing a mechanistic basis for allosteric control of SF gating." But what is the mechanism? You only see it happening, but that does not explain why it is happening. You have to either provide some mechanistic insight or tone down your claims.

Response: We thank the reviewer for raising this important point and acknowledge that we failed to make this aspect clear enough in the previous version of the manuscript. In our earlier work (PMID: 38821927), unbiased MD simulations combined with network analysis suggested

two potential allosteric pathways coupling of the pCt/M4 region to the SF. These proposed pathways were also consistent with previous mutagenesis studies discussed in that work. However, an important limitation of the previous study was that these conclusions were based mainly on unbiased MD simulations, in which only a few spontaneous transitions between the up- and down-states were observed. Consequently, the sampling was insufficient to quantitatively characterize the underlying free-energy landscape or to determine how external stimuli shift the conformational equilibrium.

We previously attempted to address this issue using several enhanced sampling approaches, but were unable to achieve satisfactory sampling convergence for this large-scale conformational transition. This limitation became a major motivation for the current study. Here, we applied the more advanced OneOPES enhanced sampling framework to quantitatively characterize the free-energy surfaces associated with the up/down transition and SF dynamics, and to investigate how phosphorylation, membrane stretch, and disease-associated mutations reshape this energetic landscape. In this way, the present work provides a more quantitative and robust assessment of the allosteric coupling mechanisms proposed in our earlier study.

We have included the following text at the beginning of the Discussion section to make these points clear: "*In our previous work, we showed that the crystallographic up- and down-state conformations of TREK-2 channel display markedly different ion conductivities in the simulations. These differences were associated with persistent conformational rearrangements at the extracellular side of the SF, particularly involving residues that stabilize the S1 ion binding site. Furthermore, network analysis of the MD trajectories, based on pairwise residue interactions that differed between two states, suggested two potential allosteric coupling pathways linking the pCt/M4 region to the SF: (i) M4 → M4 loop/EC loop → SF; (ii) M4/M2 → P2 → SF. However, as only a few spontaneous transitions between the up- and down-states were observed in these unbiased simulations, the sampling was insufficient to quantitatively characterize the conformational equilibrium or determine how external stimuli quantitatively reshape the underlying free energy landscape.*
In the present work, we revisited this problem using the enhanced sampling framework OneOPES to calculate the FESs associated with conformational changes in both the pCt/M4 region and the upper part of the SF. "

3. Figure 4. The angle does not change so how can you claim coupling? Is it just the absence of red at -120 degree? Or barrier to flipped different? Maybe you can indicate in the figure where the important changes are that you claim and explain better how that leads to coupling?

Response: The dihedral angle values themselves do not change, but, as the reviewer correctly noted, the equilibrium between the X-ray-like conductive conformation (ψ-F284=−36°) and the flipped, non-conductive conformations (ψ-F284=+149°) shifts across the different conditions. In parallel, the free-energy barrier separating these two states also changes. Thus the relevant observables are (i) the relative free energy depths of the two basins and (ii) the barrier height between them. To make the structural interpretation of the two basins more explicit, we have annotated panel Figure 4a with the characteristic ψ-F284 values corresponding to the conductive and flipped conformations. The systematic modulation of both the basin populations and barrier heights across the apo, phosphorylated, stretch-activated, and A314P systems forms the quantitative basis for the proposed coupling. We have

correspondingly expanded the legend of Fig. 4 to highlight these features more clearly. In addition, during revision, we projected our previously generated unbiased MD ensemble onto the ψ-F284 coordinate in order to better compare the conductive and non-conductive assignments obtained from ion permeation simulations with the FES minima obtained from the OneOPES (Supplementary Fig. 5).

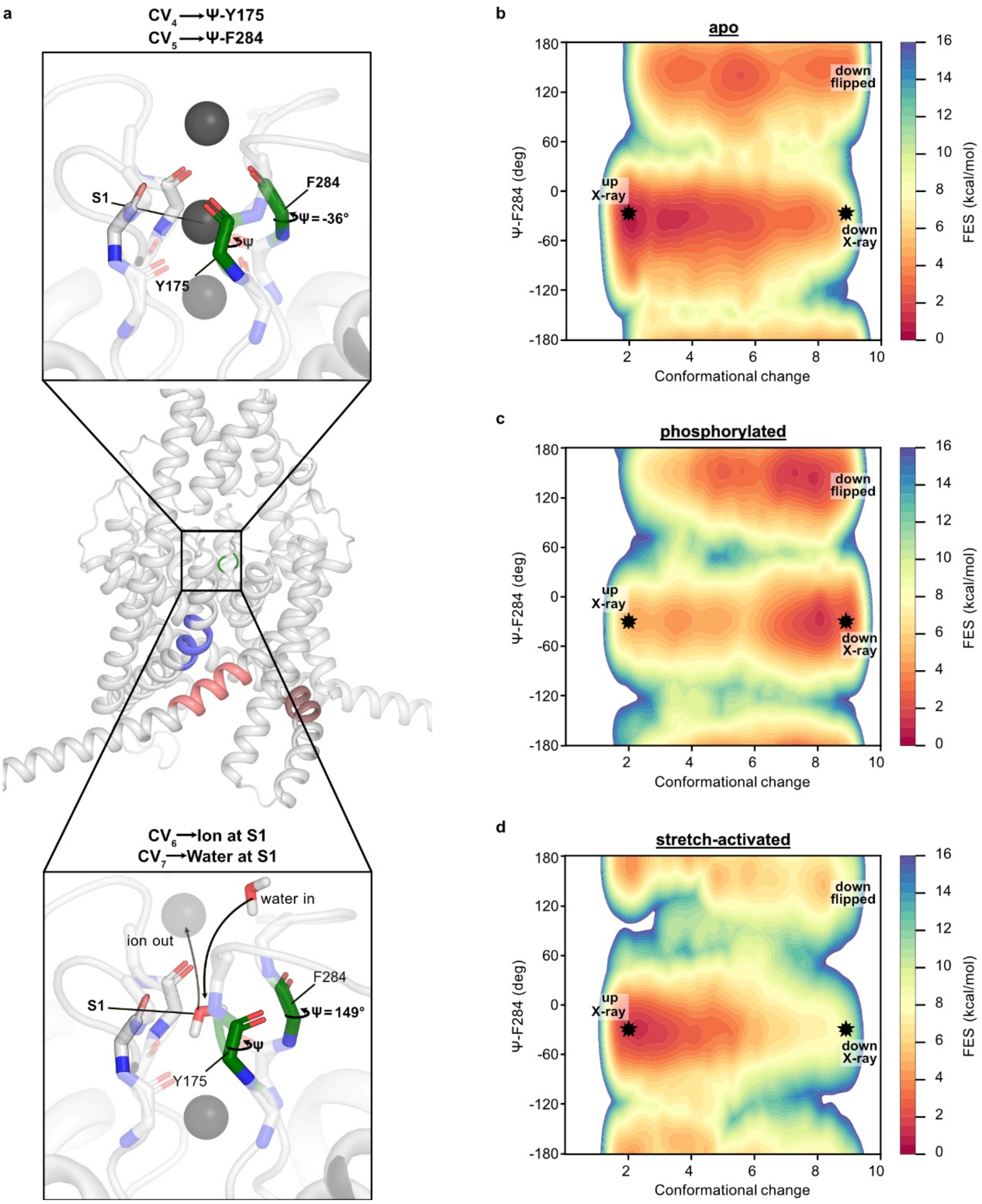

Fig. 4. Auxiliary CVs describing SF dynamics suggest the energetic coupling between pCt/M4 movement and gating-associated SF changes in the TREK-2* channel. **(a)** Schematic representation of the two auxiliary CVs used to describe the SF stability in conductive and carbonyl-flipped conformations. The conductive conformation is defined by the ψ dihedral of F284 adopting its ion-coordinating crystallographic geometry (ψ ≈ −36°), whereas the initial SF rearrangement is characterized by a flipped orientation of the F284 backbone carbonyl away from the S1 ion-binding site (ψ ≈ +149°). Two additional auxiliary CVs describing K^+ and water occupancy

at the S1 ion-binding site were further included to facilitate sampling of SF transitions and the associated rearrangements of SF residues. **(b)** 2D FES projected along the path CV and the auxiliary CV describing SF stability for apo, **(c)** phosphorylated and **(d)** membrane-stretch simulations. The average of three independent replicas for each system were calculated for final 2D FES. Black stars indicate the positions of the crystallographic TREK-2 up- and down-state structures mapped onto these coordinates, based on their measured path CV values and AdA distances. Comparison of panels (b)–(d) highlights two features that quantify the coupling between M4–pCt motion and SF gating: the relative free-energy depth of the X-ray (–30°) and flipped (+145°) ψ-F284 conformational basins, and the barrier height between them, both of which vary substantially across the apo, phosphorylated, and membrane-stretch conditions.

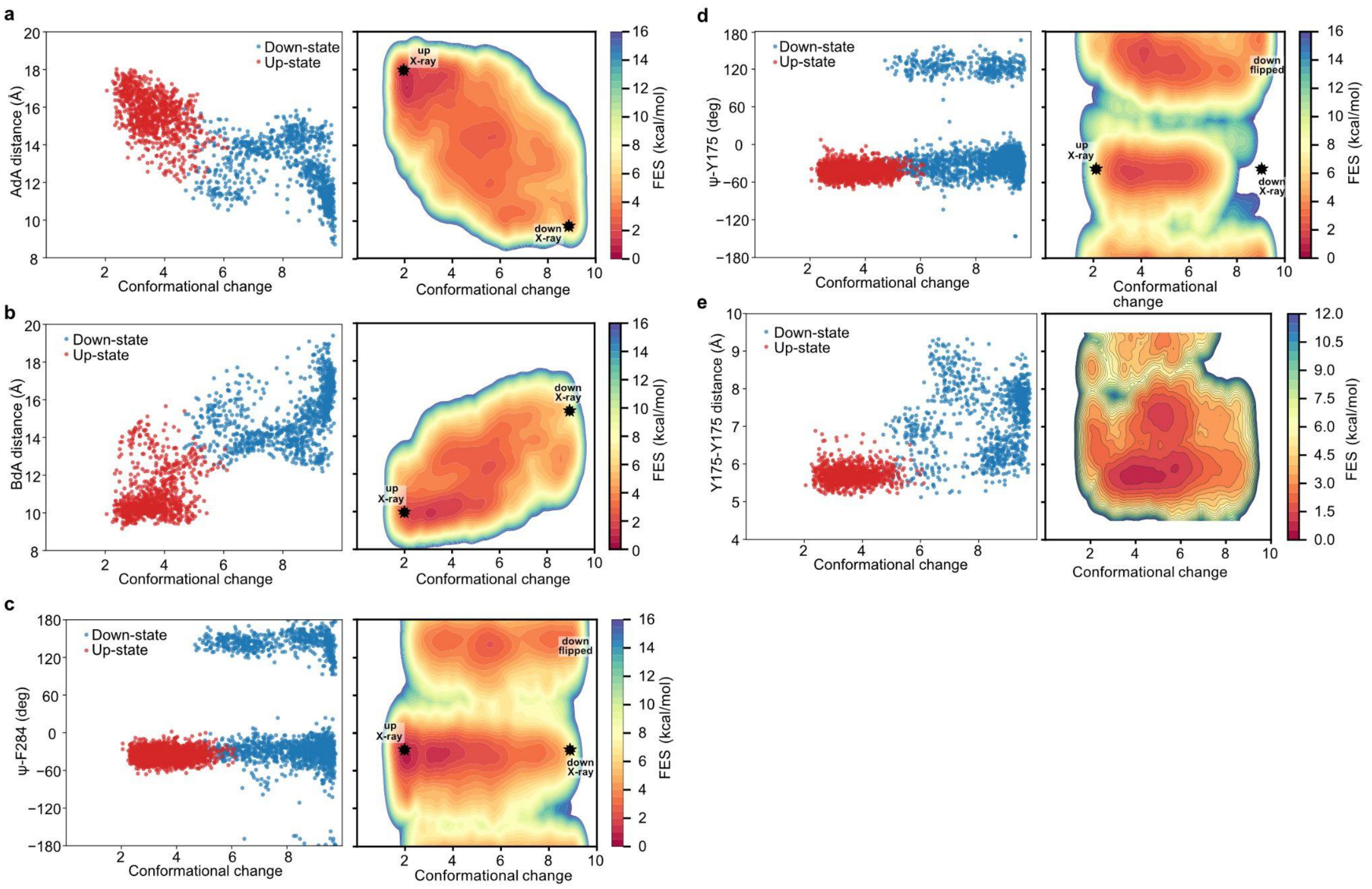

Supplementary Fig. 5. Projection of unbiased up- and down-state TREK-2* simulations onto the OneOPES collective-variable space, shown alongside the corresponding apo OneOPES free-energy surfaces. In each panel, the left plot shows conformations sampled in our previous unbiased MD simulations (ref. 17), five independent 1 μs replicas started from the up-state (red) and five from the down-state (blue), projected onto the collective variables used in the OneOPES setup and plotted against the path CV (s, "conformational change" on the x-axis), which describes the progression from the up-state (low s) to the down-state (high s). The right plot shows the two-dimensional free-energy surface (FES) reconstructed by reweighting the apo TREK-2* OneOPES ensemble (average of three independent replicas) projected along the path CV and the same variable, allowing a direct comparison between the regions spontaneously sampled in the unbiased ensemble and the energetic minima recovered by enhanced sampling. **(a)** Intra-chain M2–M4 distance (AdA, subunit A M2 to subunit A M4). **(b)** Inter-chain M4–M2 distance (BdA, subunit B M4 to subunit A M2). **(c)** Backbone ψ dihedral angle of F284 and **(d)** of Y175, both reporting on the SF conformation at the S1 K^+ binding site. **(e)** Inter-subunit Y175–Y175 backbone carbonyl distance, computed between the centres of mass of the backbone carbonyl atoms (C and O) of Y175 in the two opposing subunits. Black stars indicate the positions of the crystallographic TREK-2 up- and down-state structures mapped onto these coordinates; the "down flipped" label marks the flipped-carbonyl SF conformation accessible in the down-state region. In every panel, the unbiased up- and down-state ensembles partition into distinct regions of the path CV that coincide with the up- and down-state minima of the apo OneOPES FES. Free energies are given in kcal/mol.

4. Line 339. It is not surprising that a proline induces a kink in M4 which leads to GOF? Also, the experimental correlation that a GOF mutation will be less affected by a drug that binds closed state seems simple. Could you provide some more insightful mutational effect? Maybe

a de novo mutation that you predict the effect of and then show experimental evidence for that mechanism?

Response: We thank the reviewer for this important perspective. Our aim with the A314P simulations was to use an experimentally characterized GOF mutation as a benchmark to test whether the OneOPES framework correctly captures the direction and magnitude of the conformational shift associated with the lower-helix transition, and to quantitatively relate this shift to the NFx-based electrophysiological readout. The key value of the computational analysis lies in the quantitative agreement between the computed energetic redistribution and the experimental phenotype. Specifically, the simulations predict an almost complete collapse of the down-state free-energy basin, consistent with the experimentally observed loss of NFx sensitivity in A314P (IC_{50} shift from 3.4 to 257 µM). This agreement indicates that the OneOPES-derived free-energy surfaces are quantitatively predictive for this conformational regime. At the same time, we agree that systematic prediction and experimental validation of de novo mutations represent an important next step. However, achieving this goal will require substantial computing resources and extensive sampling efforts. We have included this discussion now in the revised version as: "*However, due to the high computational cost and the substantial variability observed across individual simulation runs of the A314P mutant, which complicates both convergence and interpretation, we are currently limited in systematically assessing how these different mutations influence the up-to-down transition of M4 and the associated conformational changes in the SF. More systematic investigations, involving substantially longer sampling times and/or the inclusion of additional CVs will therefore be required in future studies*."

5. Line 350-354. Can you show some actual structural overlays to further strengthen the similarities of your models to the structure?

Response: We thank the reviewer for this valuable suggestion. To strengthen the comparison beyond projecting the TRAAK A270P structure onto the 2D FES (Fig. 5e,f), we have added a direct structural overlay as a new Supplementary Figure (Supplementary Fig. 8), together with the corresponding geometric measurements in a new Supplementary Table (Supplementary Table 4). A representative structure from the A314P TREK-2* up-state free-energy minimum was superimposed, based on the conserved pore domain, onto the experimentally determined TRAAK A270P structure (PDB 7LJ4) and, for comparison, the down-state TRAAK structure (PDB 4WFF). The overlay shows that the simulated TREK A314P model reproduces the experimental TRAAK A270P conformation: M4 adopts the very similar kinked, up-state geometry, and the two structures superimpose closely, whereas the WT down-state M4 is displaced downward and away from the pore. This agreement is further quantified by two measurements, the local M4 kink angle and the inter-subunit M4–M2 contact distance between L320 (M4) and P198 (M2) of the opposing subunit. A corresponding description has been added to the Results as "*Direct structural comparison provides further insight into the up-state stabilization in the mutant. A representative structure extracted from the A314P up-state minimum shows that the proline substitution introduces a kink in M4 (~66°; Supplementary Fig. 8 and Supplementary Table 4), which laterally displaces the pCt–M4 interface toward the opposing M2 helix and brings L320 (M4) into close contact with P198 (M2) of the neighbouring subunit (3.7–4.1 Å). This geometry is very similar to the experimentally determined TRAAK A270P mutant structure[44] and contrasts with the TRAAK WT down-state, in which the corresponding contact is longer (4.6–7.1 Å).*"

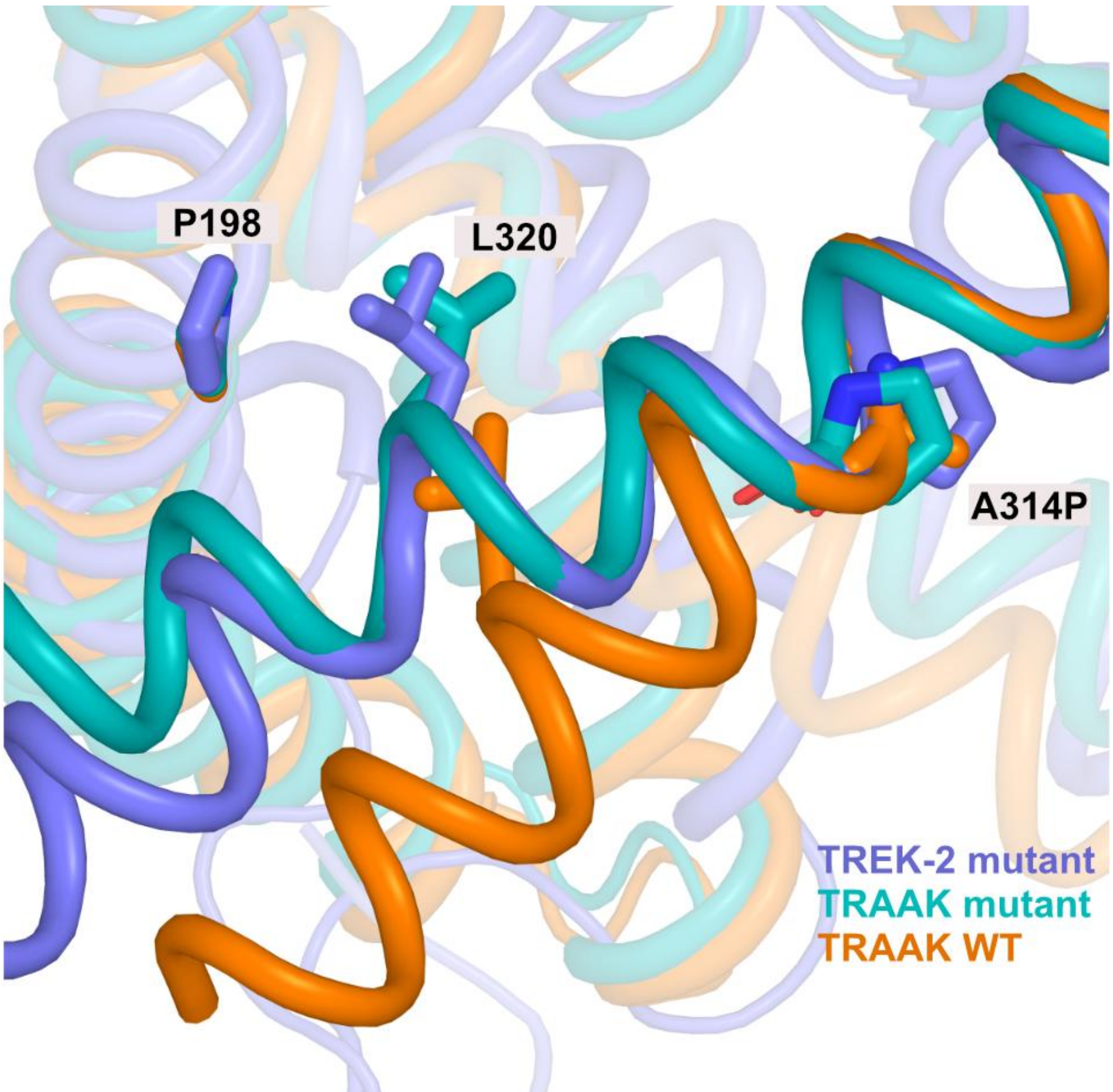

Supplementary Fig. 8. Structural comparison of the A314P-induced M4 kink in TREK-2. Overlay of a representative structure from the A314P TREK-2* up-state free-energy minimum (purple) with the experimentally determined TRAAK A270P structure (teal; PDB: 7LJ4) and the down-state TRAAK structure (orange; PDB: 4WFF). Structures were superimposed on the conserved pore domain so that the position of M4 relative to the rest of the channel is directly comparable; the view is centred on the M4 helix and the intracellular pCt–M4 interface. The substituted residue (A314P in TREK-2*; A270P in TRAAK), the M4 leucine (L320 in TREK-2*; L276 in TRAAK) and the M2 proline of the opposing subunit (P198 in TREK-2*; P155 in TRAAK) are shown as sticks. In both the TREK-2* A314P model and the TRAAK A270P structure, M4 adopts the kinked, upward-displaced up-state conformation and the two structures overlay closely, whereas in the TRAAK WT structure M4 is in the downward (down-state) position. The L320–P198 (L276–P155) side chains are in close contact in the two mutant structures, indicating that the kinked M4 conformation positions the pCt–M4 interface adjacent to the opposing M2 helix.

Supplementary Table 4. M4 kink angle and inter-subunit M4–M2 contact distance for the A314P TREK-2* and reference WT TRAAK structures. Values are reported for a representative structure selected from the A314P TREK-2* up-state free-energy minimum, the experimentally determined TRAAK A270P structure (PDB: 7LJ4), and the down-state TRAAK structure (PDB: 4WFF). The M4 kink angle quantifies the local bend of M4 at the substituted position. The M4-M2 distance corresponds to the minimum side-chain heavy-atom distance between the M4 leucine (L320 in TREK-2*; L276 in TRAAK) and the M2 proline of the opposing subunit (P198 in TREK-2*; P155 in TRAAK).. Both proline-mutated structures (TREK-2* A314P, TRAAK A270P) show a larger M4 kink and a shorter intersubunit M4-M2 contact than the TRAAK WT down-state.

Structure	Kink angle chain A (°)	Kink angle chain B (°)	M4–M2 distance pair 1 (Å)	M4–M2 distance pair 2 (Å)

A314P (model)	67.3	65.4	4.1	3.7
TRAAK A270P (PDB 7LJ4)	62.9	63.7	4.2	3.6
TRAAK WT (PDB 4WFF)	58.7	55.7	7.1	4.6

6. Line 383. "capture the complex allosteric coupling between M4-pCt motions and the SF gating". Did you really show this? You see it happening, but you do not provide the mechanism why it is happening.

Response: As discussed before, this interpretation is based on our previous unbiased MD simulations, which showed that the up- and down-state exhibit distinct conductive properties and that phosphorylation promotes both the up-to-down conformational transition and the SF inactivation. The present OneOPES free-energy calculations further substantiate these observations by providing a quantitative energetic characterization of the coupling between these processes. We acknowledge that this mechanistic connection was discussed sufficiently clearly in the previous version. We have removed this original sentence in the revised version and clarified this discussion in the main text as follows: "*In summary, together with our previous unbiased MD simulations, the free-energy landscapes obtained from OneOPES provide a more coherent energetic framework for understanding how chemical, mechanical, and mutational perturbations modulate TREK-2 function through shared structural pathways and define the channel's gating landscape. These findings are consistent with a very recent study from some of us, in which electrophysiology measurements suggested that diverse physiological stimuli activate TREK/TRAAK channels through a common mechanistic pathway. Importantly, the unbiased MD simulations were essential for identifying the complex interaction network coupling the pCt/M4 region to the SF and for defining the key structural descriptors that best characterize the essential conformational transition. In contrast, the state-of-the-art enhanced sampling approach applied here was crucial for quantifying the free-energy changes associated with these transitions and for determining how they are reshaped by different physiological perturbations. Together, these results highlight the complementary strengths of unbiased and enhanced sampling MD approaches in dissecting regulatory mechanisms in complex biological systems and underscore the importance of their integration.*"

Minor comments.

1. Line 153. Explain in main text how stretch was applied. Now only explained in Methods.

Response: We have included the following description into the main text: "mechanical stretch which was simulated by applying a negative lateral pressure of −50 bar in the bilayer plane while maintaining +1 bar in the bilayer normal direction, at 300 K (see Methods for detailed setup)"

2. Line 72-76. This sentence sounds strange to me: "Electron density…made accessible through fenestration". Maybe better to break the sentence up in two parts and explain everything better?

Response: We agree with the reviewer and reformulated this sentence as: "*Notably, the up- and down-state crystal structures of TREK channels exhibit nearly identical SF conformations, which argues against a canonical gate-switching mechanism at the filter. In the down-state, however, lateral fenestrations open and expose the region beneath the SF to the membrane, where additional electron density has been attributed to lipid acyl-chains. This observation led to the proposal of a lipid-block mechanism to explain the reduced conductance of the down-state. In contrast, some electrophysiological and computational studies, including our own, supported a model in which the SF itself serves as the primary gate in K2P channels*."

3. Line 186&188. AdA and BdA? Maybe explain better the nomenclature: "Between subunit A and A= AdA" etc.

Response: We agree and have revised the description of the auxiliary CVs to explain the nomenclature more clearly. "*AdA denotes the M4–M2 distance within the same subunit (subunit A to subunit A, i.e. intra-chain), and BdA denotes the M4–M2 distance between opposite subunits (subunit B M4 to subunit A M2, i.e. inter-chain).*" These definitions have now been added in the legend of Fig. 3 and in the Results section, following explanation has been included: "*One auxiliary CV (AdA, denoting the M2–M4 distance from subunit A to subunit A, i.e. intra-chain) tracks the distance between the M4 and M2 helix within the same subunit, which decreases as the channel moves towards the down-state. The second CV (BdA, denoting the M2 of subunit B to the M4 of subunit A, i.e. inter-chain) monitors the distance between the M4 and M2 helix of opposite subunits, which instead decreases towards the up-state.* "

4. Line 360. Is the figure referred to simulated under stretch or for the mutation? Probably for the mutation. And it is in 5f, not 5e.

Response: Thank you for pointing out this mistake. We have corrected it correctly.

5. Line 536. Is the value of stretch you provide consistent with physiological values of stretch? Some reference to this would be good to include.

Response: We thank the reviewer for raising this important point. Under semi-isotropic pressure coupling, the surface tension γ of a planar bilayer is given by $\gamma = d \cdot (P_N - P_L)$, where d is the bilayer thickness (P–P inter-leaflet distance), P_N is the pressure normal to the bilayer, and $P_L = P_{XX} = P_{YY}$ is the lateral pressure in the bilayer plane. For our POPC bilayer at 300 K with $d \approx 4$ nm, $P_N = +1$ bar, and $P_L = -50$ bar, this corresponds to an effective surface tension of $\gamma = 20$ mN m^{-1}. For comparison, physiological membranes are generally close to a relaxed state, whereas excised membrane patches exhibit a basal tension of ~0.5–4 mN m^{-1}, and patch pressurization can reach lytic tensions of ~12 mN m^{-1} (PMID: 24550493). Mechanosensitive K2P channels such as TRAAK and TREK are activated across this range, from near-baseal activity at low tension to saturation around ~12 mN m^{-1} (PMID: 24550493). Our simulated tension of ~20 mN m^{-1} exceeds the experimental saturation/lytic limit, but remains within the same order of magnitude as the experimentally relevant activation regime. Similar elevated tensions have also been applied in previous all-atom MD simulations of mechanosensitive K2P channels to accelerate conformational transition (PMID: 28392258; 29590591). Importantly, bilayer integrity remained stable throughout the simulations (as indicated in the new SI Figure 12,13). We assume that the state captured in our stretch-activated simulations corresponds

to a fully stretch-activated channel rather than to a resting or partially activated condition. We have included now following explanation to the Method section: “*Across a ~4 nm-thick POPC bilayer, the applied lateral pressure corresponds to an effective surface tension of* $\gamma = d \times (P_N - P_L) \approx 4\ nm \times 51\ bar \approx 20\ mN\ m^{-1}$*, consistent with values used in previous all-atom MD studies of mechanosensitive K2P channels*[10],[43]*. For comparison, excised-patch experiments exhibit basal tensions of ~0.5–4* $mN\ m^{-1}$ *and reach lytic tensions of ~12* $mN\ m^{-1}$ *upon pressurization, with TRAAK and TREK channels activating across this range and saturating near 12* $mN\ m^{-1}$ *(PMID: 24550493). The simulated tension is supra-saturating but represents the in-silico activation regime commonly used for this family, to drive the conformational transition within the μs timescales accessible to atomistic MD simulation.*”

6. Line 644. Equation is definitely wrong.

Response: We thank the reviewer for noting this mistake and have revised the equation as:

$$\frac{I}{I_0} = a + \frac{1 - a}{1 + \left(\frac{x}{IC_{50}}\right)^h}$$

where I and I0 is the current with and without NFx/TPenA, a is the fraction of initial or residual current, x is the NFx/TPenA concentration, IC50 is the concentration at half maximal inhibition, and h is the Hill coefficient.

REVIEWER COMMENTS

Reviewer #1 (Remarks to the Author):

The authors have put genuine effort into this revision and several improvements are real and accepted. The structural overlay for A314P with quantified kink angles and contact distances (Supp. Fig. 8, Table 4) is a solid addition. The membrane equilibration data (Supp. Figs. 12–13) address the earlier gap appropriately. The ΔΔG comparison with propagated uncertainties replaces a purely visual argument with something more quantitative, and the decision not to apply a formal statistical test at n=3 is reasonable and honestly explained. The mutation numbering discrepancy is now properly resolved with the new supplementary table. The revised Fig. 6 legend, replacing "non-conductive" with "initial SF conformational changes," is a meaningful correction. These points require no further attention.

Answer: We thank the reviewer for the careful assessment and for the positive feedback.

Three concerns remain.

The functional labeling of the FES minima is not independently supported in this manuscript. The authors describe certain FES minima as corresponding to "conductive" and "non-conductive" SF states, primarily through the ψ-F284 dihedral angle. The new Supp. Fig. 9, which I asked for, shows that S2–S4 ion occupancies and Y175–Y175 inter-subunit distances do not vary substantially across conditions. The authors acknowledge this honestly in the rebuttal and explain that those parameters were not biased during the simulations. The problem is that this acknowledgment does not propagate into the main text. The functional assignment — which FES minimum is conductive and which is not — comes entirely from ref 17, where voltage-driven simulations with real permeation events and full ion occupancy profiles established that correspondence. In the current study there is no voltage, no permeation, and now the additional SF parameters are confirmed to be flat. I am not asking the authors to run permeation simulations — that would be a different study. I am asking them to state clearly in the main text that the functional identity of the FES states relies on the prior work and is not independently demonstrated here. The revised Fig. 6 legend is a step in this direction, but the same distinction needs to be applied consistently throughout results and discussion, wherever the language implies that the simulated states are known to be conductive or non-conductive.

Answer: We thank the reviewer for this important comment and have added the following statement in the Results section on page 11: "*Furthermore, it should be noted that the functional assignment of the conductance property of the SF is based solely on our previous ion permeation simulations and is not a direct outcome of the present study.*"

Additionally, we revised the corresponding part in the Discussion section on page 18 as follows "*Within the up-state basin, the previously assigned conductive crystallographic SF configuration*[17] *represents the dominant thermodynamic minimum, whereas a secondary carbonyl-flipped conformer of F284 is separated by a substantial free-energy barrier.*"

Further changes are made on the caption of the figure 4: "*Schematic representation of the two auxiliary CVs used to describe the SF stability in canonical and carbonyl-flipped conformations. The canonical conformation is defined by the ψ dihedral of F284 adopting its ion-coordinating crystallographic geometry (ψ ≈ −36°), whereas the initial SF rearrangement is characterized by a flipped orientation of the F284 backbone carbonyl away from the S1 ion-binding site (ψ ≈ +149°).*"

The z-distribution confirms the limitation rather than addressing it.
Supp. Fig. 2 shows that z stays below approximately 0.2 nm^2 throughout all simulations. The authors present this as reassurance that the path CV is adequate. The interpretation is the opposite of what they claim. A system that remains tightly confined to the morphed tube for the entire simulation provides no information about whether that tube is the only relevant conformational pathway, or whether energetically accessible states exist outside it. The multi-thermal replicas did not drive the system off the path in any observable way. The discussion already contains one sentence noting this limitation, which I acknowledge. But the abstract and results still present the FES as a characterization of the TREK-2 conformational landscape, when what is characterized is the energetics along a predefined axis between two known crystal structures. These are not the same thing, and the text should say so.

Answer: We thank the reviewer for this helpful comment. To clarify this point, we have revised the corresponding paragraph in the Results section (page 7) as follows:" *As with other path-based enhanced sampling approaches, the CV is designed to describe the essential dynamics connecting these predefined reference states and are therefore less effective at sampling conformations that lie far from the interpolated transition pathway, as also reflected in Supplementary Fig. 1.*" And "*Convergence analysis of the OneOPES bias potential together with the time evolution of the auxiliary CVs, further supports the robustness of sampling along the predefined pathway across all replicas (Fig. 2e and Supplementary Fig. 2,3)..*"

Furthermore, we also stated this more explicitly now in the abstract: "*Here, using the OneOPES framework, which unifies multiple enhanced-sampling molecular dynamics strategies, we provide an energetic characterization of the conformational transition along a predefined pathway connecting the up and down states of TREK-2, together with initial conformational rearrangements of the selectivity filter of TREK-2 under physiological stimuli and disease-mimicking conditions.*"

The circular argument in Section IV persists in the main text.

The rebuttal correctly acknowledges that the co-variation between the path CV and the M4–M2 auxiliary CVs is "a geometric consequence of the path CV construction." This was the right response to my concern. However, the revised main text immediately follows that acknowledgment with the statement that "M4-pCt motion both shifts the thermodynamical equilibrium of the SF and modulates the local barrier associated with carbonyl flipping, thereby providing an energetic basis for the thermodynamic coupling." This sentence re-introduces the causal claim through the back door. A reader of the manuscript who has not read the rebuttal will encounter the acknowledgment and the causal claim in the same paragraph and will not see the contradiction. What the FES shows is a thermodynamic association between two coordinates that are partially linked by the geometric definition of the path. This is a useful result. It is not, however, evidence that M4 motion causes SF rearrangement. The language in the main text should consistently reflect the former rather than the latter.

Answer: We agree with the reviewer that the corresponding Results section contained statements that were not fully consistent. We have therefore revised the relevant text on page 11, and in particular, removed the sentence “M4-pCt motion both shifts the thermodynamical equilibrium of the SF and modulates the local barrier associated with carbonyl flipping, thereby providing an energetic basis for the thermodynamic coupling."

Now these two sections read: “*After establishing the energetic correspondence between the path CV and dynamics of the M4 helices, we next examined whether the conformational transitions at the M4-pCt are causally linked to the conformational behavior of the SF.*”
“*Together these results support the view that, under basal conditions, TREK-2* dynamically exchanges between conductive X-ray-like and F284 carbonyl-flipped states. We acknowledge that part of the observed co-variation between the path CV and the SF dihedral observables again arises from the geometric consequence of the path CV construction itself, as the up- and down-state crystal structures used to define the path may slightly differ in their SF environment, even though both crystallographic states adopt the same conductive SF conformation.*”

One minor point.
The AlphaFold3 prediction used to justify the helical pCt conformation (Supp. Fig. 10) appears to fall in the pLDDT range of 50–70 for the relevant segment. This range is classified by AlphaFold itself as low confidence. The authors should state the pLDDT value explicitly and acknowledge that the helical conformation of the modelled pCt remains a working assumption, not a structurally validated feature.

Answer: The pLDDT scores for the pCt region range from 70 to 90, which generally indicates that the backbone conformation can be predicted with relatively high confidence. Nevertheless, we agree that this point should be stated more explicitly in the Method section. We have now added the following sentence on page 23: “*The choice of this initial starting conformation is supported by AlphaFold3*[56] *predictions of the full-length TREK-2 structure*

(Supplementary Fig. 10), in which the pCt region is predicted to adopt a helical structure with pLDDT scores ranging from 70 to 90."

Reviewer #2 (Remarks to the Author):
I thank the authors for their rigorous revision of the manuscript. The paper is now ready for publication.

Reviewer #3 (Remarks to the Author):

The authors have responded well to my comments.
